# Palmitoylation acts as a checkpoint for MAVS aggregation to promote antiviral innate immune responses

**DOI:** 10.1172/JCI177924

**Published:** 2024-12-02

**Authors:** Liqiu Wang, Mengqiu Li, Guangyu Lian, Shuai Yang, Jing Cai, Zhe Cai, Yaoxing Wu, Jun Cui

**Affiliations:** 1 MOE Key Laboratory of Gene Function and Regulation, Guangdong Province Key Laboratory of Pharmaceutical Functional Genes, State Key Laboratory of Biocontrol, Innovation Center of the Sixth Affiliated Hospital, School of Life Sciences of Sun Yat-sen University, Guangzhou, Guangdong, China.; 2 Guangzhou Institute of Pediatrics, Guangzhou Women and Children’s Medical Center, Guangzhou, Guangdong, China.; 3 Department of Critical Care Medicine, The First Affiliated Hospital of Sun Yat-sen University, Guangzhou, Guangdong, China.

**Keywords:** Cell biology, Immunology, Innate immunity, Molecular biology

## Abstract

Upon RNA virus infection, the signaling adaptor MAVS forms functional prion-like aggregates on the mitochondrial outer membrane, which serve as a central hub that links virus recognition to downstream antiviral innate immune responses. Multiple mechanisms regulating MAVS activation have been revealed; however, the checkpoint governing MAVS aggregation remains elusive. Here, we demonstrated that the palmitoylation of MAVS at cysteine 79 (C79), which is catalyzed mainly by the palmitoyl *S*-acyltransferase ZDHHC12, was essential for MAVS aggregation and antiviral innate immunity upon viral infection in macrophages. Notably, the systemic lupus erythematosus–associated mutation MAVS C79F was associated with defective palmitoylation, resulting in low type I interferon (IFN) production. Accordingly, *Zdhhc12* deficiency apparently impaired RNA virus–induced type I IFN responses, and *Zdhhc12*-deficient mice were highly susceptible to lethal viral infection. These findings reveal a previously unknown mechanism by which the palmitoylation of MAVS is a checkpoint for its aggregation during viral infection to ensure timely activation of antiviral defense.

## Abstract 2

Palmitoylation of MAVS is a checkpoint for its aggregation during viral infection to ensure timely activation of antiviral defense

## Introduction

Innate immunity recognizes pathogen-associated molecular patterns (PAMPs) through pattern recognition receptors (PRRs), such as Toll-like receptors (TLRs), retinoic acid–inducible gene I–like (RIG-I–like) receptors (RLRs), nucleotide-binding oligomerization domain–like (NOD-like) receptors (NLRs), and several DNA sensors, to provide the first line of host defense against invading pathogens (1, 2). Upon viral infection, PRRs detect virus-derived nucleic acids and recruit adaptor proteins, including TIR domain–containing adaptor molecule 1 (TRIF), mitochondrial antiviral-signaling protein (MAVS), and stimulator of interferon genes protein (STING), which further activate the nuclear factor κ-light-chain enhancer of activated B cells (NF-κB), interferon regulatory 3/7 (IRF3/7), and inflammasome signaling pathways to trigger the production of type I interferons (IFNs) and proinflammatory cytokines (3–6). Secreted IFNs induce expression of IFN-stimulated genes (ISGs), leading to the establishment of an innate immune state against invading infections (7, 8).

Upon RNA virus infection, the signaling adaptor MAVS undergoes conformational changes and prion-like aggregation through its CARD domain, which is pivotal for propagating antiviral innate immune responses (9, 10). Previous studies have revealed multiple mechanisms that play critical roles in triggering antiviral innate immunity through MAVS (11, 12). For example, the K63-linked ubiquitination of MAVS, along with the interaction between MAVS and unanchored K63-linked polyubiquitin chains with RIG-I, facilitates the aggregation of MAVS (9, 13). However, the checkpoint governing MAVS aggregation remains to be elucidated.

In this study, through cell-based screening, we demonstrated that ZDHHC12-mediated palmitoylation of MAVS at C79 is essential for its aggregation in macrophages. Consistently, *Zdhhc12-*deficient mice produce fewer type I IFNs and are more susceptible to RNA virus infection. Interestingly, the K63-linked ubiquitination of MAVS serves as a critical signal to recruit palmitoyl *S*-acyltransferase for its subsequent palmitoylation. Taken together, our findings provide valuable insights into the mechanism by which MAVS palmitoylation acts as a checkpoint for MAVS aggregation and subsequent activation of antiviral immune responses.

## Results

### Palmitoylation of MAVS at C79 is essential for the induction of the type I IFN response.

**Figure 1:**
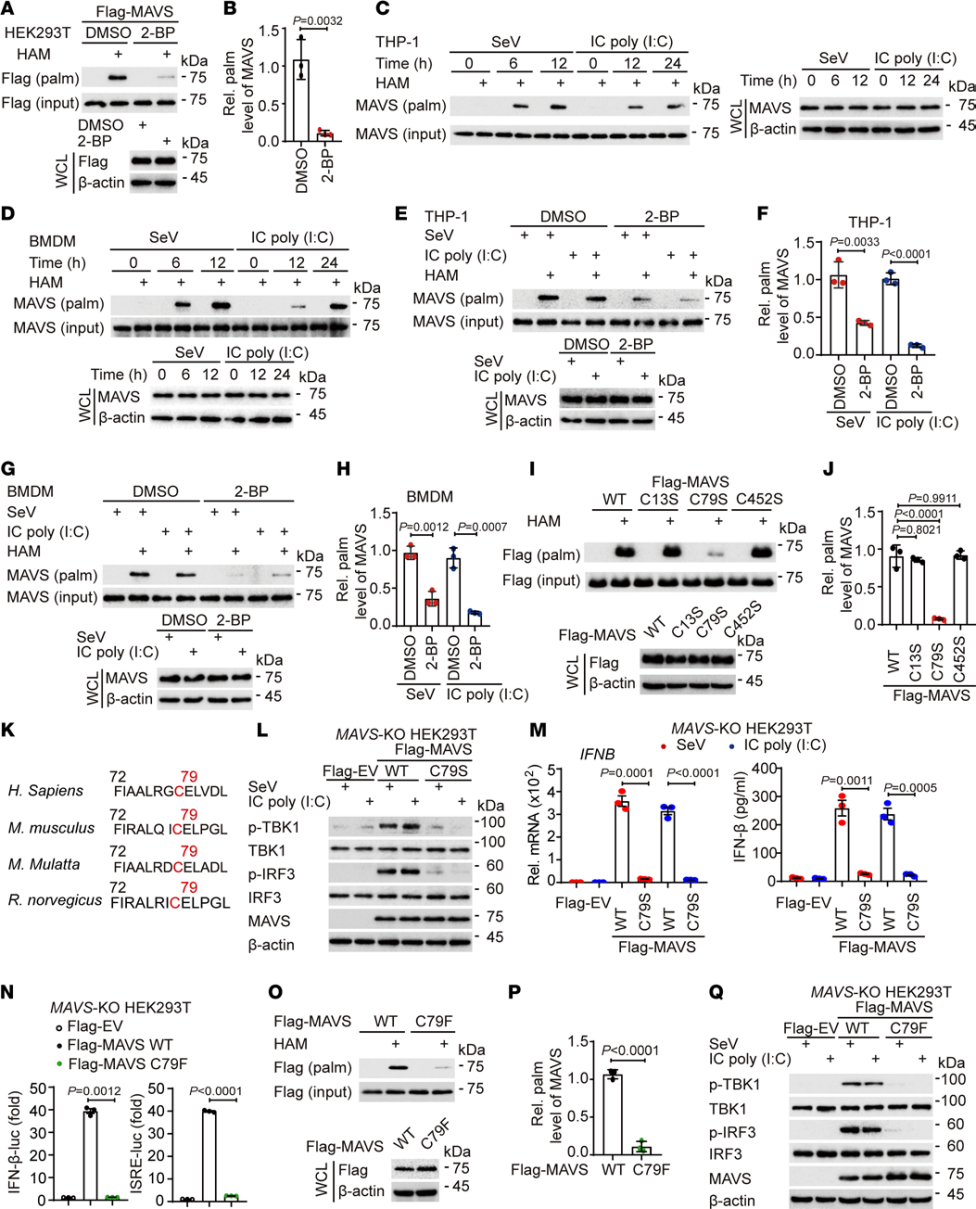
Palmitoylation of MAVS at C79 is critical for RLR signaling activation. (**A** and **B**) HEK293T cells transfected with FLAG-MAVS were treated with DMSO or 2-bromopalmitate (2-BP; 50 μM/12 h) to assess MAVS palmitoylation via acyl-biotin exchange (ABE) and immunoblot analysis. WCL, whole-cell lysates. (**C** and **D**) THP-1–derived macrophages (**C**) or BMDMs (**D**) were treated with SeV (MOI = 1) or intracellular (IC) poly(I:C) (5 μg/mL) to measure MAVS palmitoylation levels. (**E**–**H**) Cells pretreated with DMSO or 2-BP followed by SeV or IC poly(I:C) treatment showed altered MAVS palmitoylation levels in THP-1–derived macrophages (**E** and **F**) or BMDMs (**G** and **H**). (**I** and **J**) HEK293T cells transfected with wild-type (WT) FLAG-MAVS or MAVS mutants were evaluated for their palmitoylation levels using ABE. (**K**) Alignment of MAVS sequences from various species highlighting the palmitoylation site. (**L** and **M**) *MAVS*-knockout (*MAVS*-KO) HEK293T cells were transfected with indicated plasmids, followed by SeV or IC poly(I:C) treatment. Cell lysates and supernatants were collected for immunoblot, real-time qPCR analysis, and ELISA. (**N**) Luciferase reporter assays in *MAVS*-KO HEK293T cells indicated functional differences between WT and C79F MAVS. (**O** and **P**) ABE assay and immunoblot analysis were performed in HEK293T cells transfected with WT or C79F MAVS. (**Q**) *MAVS*-KO HEK293T cells were transfected with indicated plasmids, followed by SeV or IC poly(I:C) treatment. Cell lysates and supernatants were collected for immunoblot analysis. In **A**, **C**–**E**, **G**, **I**, **L**, **O**, and **Q**, similar results were obtained for 3 independent experiments. In **B**, **F**, **H**, **J**, and **P**, data are presented as mean values ± SD. In **M** and **N**, data are presented as mean values ± SEM. Statistical analysis was performed using 2-tailed Student’s *t* test in **B**, **F**, **H**, **M**, and **P** or 1-way ANOVA multiple comparisons in **J** and **N**.

Extensive studies have demonstrated that palmitoylation plays critical roles in modulating immune responses by targeting immune regulators (e.g., NOD2, NLRP3, cGAS, and STING) (14–21). However, the role of palmitoylation in regulating MAVS function remains to be elucidated. To determine whether MAVS can be modified by palmitoylation, we detected MAVS palmitoylation using an acyl-biotin exchange (ABE) assay in which the thioester linkages of the free cysteine thiol groups of MAVS were irreversibly blocked by *N*-ethylmaleimide and the palmitoylated cysteines were exposed to hydroxylamine (HAM). We observed that MAVS was modified by palmitoylation and its palmitoylation level was substantially decreased in human embryonic kidney (HEK) 293T cells treated with the inhibitor 2-bromopalmitate (2-BP) (Figure 1, A and B). The signal of MAVS palmitoylation was not detected in the resting state, whereas the level of MAVS palmitoylation increased with Sendai virus (SeV) or intracellular (IC) polyinosinic:polycytidylic acid [poly(I:C)] treatment in a time-dependent manner in both human myeloid leukemia mononuclear cell (THP-1)–derived macrophages and mouse bone marrow–derived macrophages (BMDMs) (Figure 1, C and D). Consistently, 2-BP also markedly reduced SeV- or IC poly(I:C)–induced MAVS palmitoylation (Figure 1, E–H). These data suggest that MAVS undergoes palmitoylation upon RNA virus infection in both human and murine macrophages. Based on in silico analysis (https://swisspalm.org/), we next identified cysteine residues 13, 79, and 452 of human MAVS as potential palmitoylation sites and found that the mutation of C79S in MAVS almost completely abrogated its palmitoylation (Figure 1, I and J), indicating that C79 is the major palmitoylation site of MAVS and is mostly conserved across different species (Figure 1K).

We next assessed whether MAVS palmitoylation regulated the RLR-induced type I IFN response. Upon SeV or IC poly(I:C) treatment in *MAVS*-knockout (*MAVS*-KO) HEK293T cells, wild-type (WT) MAVS triggered the phosphorylation of TBK1 and IRF3, and the expression and secretion of IFN-β were markedly greater than those in palmitoylation-deficient mutant MAVS C79S–transfected cells (Figure 1, L and M). Notably, the systemic lupus erythematosus–related (SLE-related) mutation MAVS C79F (22), a loss-of-function variant of MAVS (Figure 1N), was associated with defective palmitoylation (Figure 1, O and P). Moreover, MAVS C79F lost its ability to trigger the activation of type I IFN signaling under SeV or IC poly(I:C) treatment conditions (Figure 1Q), suggesting that SLE patients with MAVS C79F are at increased risk of viral infection. Collectively, these results indicate that palmitoylation of MAVS at C79 is essential for the activation of the RLR-induced type I IFN response.

### ZDHHC12 is a major palmitoyl S-acyltransferase for MAVS palmitoylation in macrophages.

**Figure 2:**
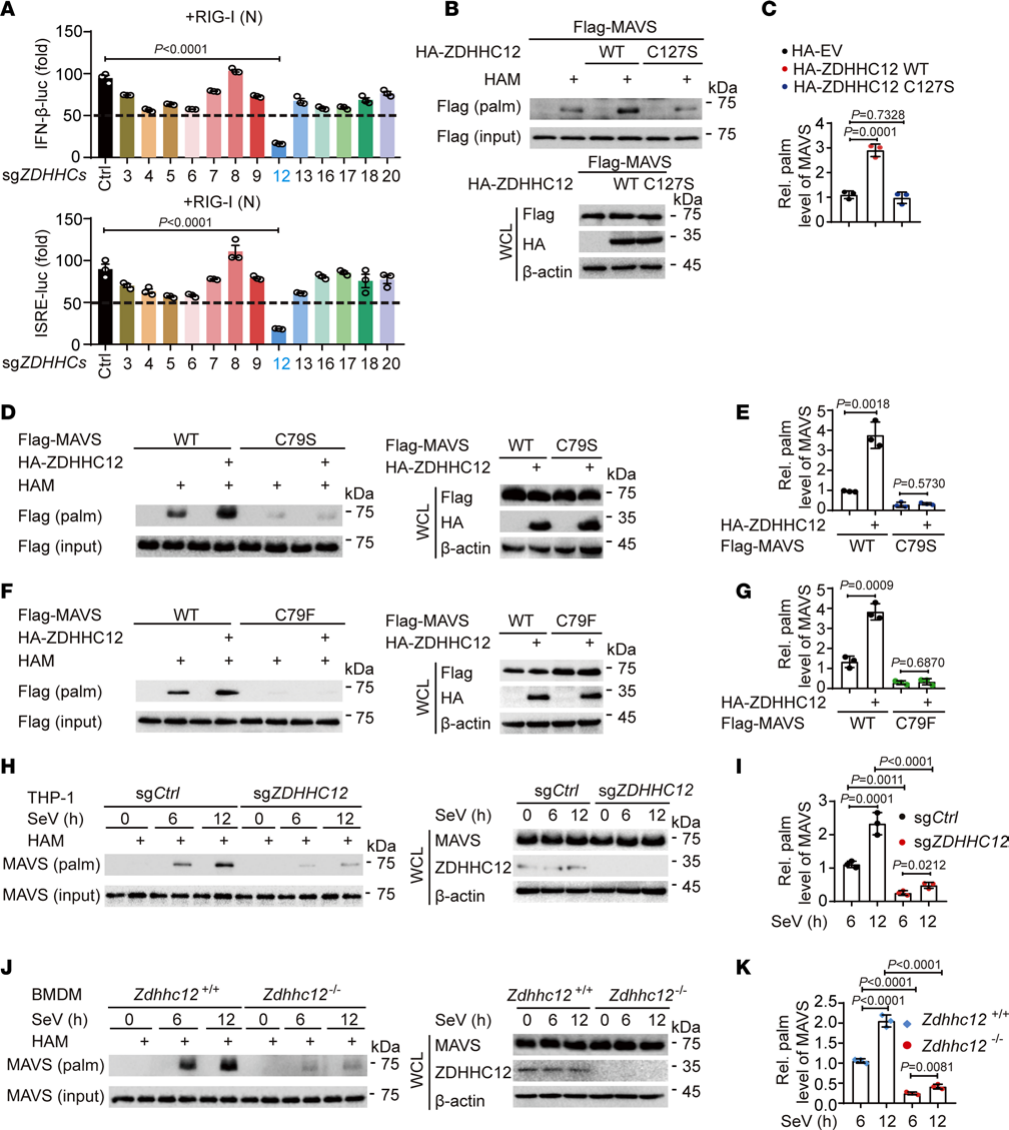
Palmitoylation of MAVS is mainly mediated by ZDHHC12 in macrophages. (**A**) HEK293T cells were transfected with control single-guide RNA (sg*Ctrl*) or sgRNA targeting specific *ZDHHCs* (sg*ZDHHCs*) for 48 hours, followed by transfection with IFN-β-luc or ISRE-luc reporter plasmids and RIG-I (N) for 24 hours. Cell lysates were collected for luciferase reporter assays. (**B** and **C**) HEK293T cells were transfected with indicated plasmids for 24 hours. Cell lysates were collected for ABE assay and immunoblot analysis. (**D**–**G**) HEK293T cells were transfected with indicated plasmids in the presence of HA-ZDHHC12 or not for 24 hours. Cell lysates were collected for ABE assay and immunoblot analysis. (**H**–**K**) WT (sg*Ctrl*) or *ZDHHC12*-KO (sg*ZDHHC12*) THP-1 macrophages (**H** and **I**) and *Zdhhc12^+/+^* or *Zdhhc12^–/–^* BMDMs (**J** and **K**) were infected with SeV (MOI = 1) for indicated time periods. Cell lysates were collected for ABE assay and immunoblot analysis. In **A**, data are presented as mean values ± SEM. In **B**, **D**, **F**, **H**, and **J**, similar results were obtained for 3 independent experiments. In **C**, **E**, **G**, **I**, and **K**, data are presented as mean values ± SD. Statistical analysis was performed using 2-tailed Student’s *t* test in **E** and **G** or 1-way ANOVA multiple comparisons in **A**, **C**, **I**, and **K**.

Protein palmitoylation is catalyzed by the palmitoyl *S*-acyltransferase family, which consists of 23 ZDHHC members. To identify which palmitoyl *S*-acyltransferase is responsible for MAVS palmitoylation, we examined the relative expression levels of 23 ZDHHC proteins in HEK293T and THP-1 cells and observed that *ZDHHC3–9*, *ZDHHC12*, *ZDHHC13*, *ZDHHC16–18*, and *ZDHHC20* were highly expressed in both cell types (Supplemental Figure 1, A and B; supplemental material available online with this article; https://doi.org/10.1172/JCI177924DS1). We subsequently performed a single-guide RNA–mediated (sgRNA-mediated) knockdown of *ZDHHC* expression followed by an IFN-β and IFN-stimulated response element (ISRE) promoter–driven luciferase reporter assay in HEK293T cells and found that the type I IFN activation induced by RIG-I-N (N-terminal caspase recruitment domain of RIG-I, an active deletion of RIG-I) was apparently inhibited by *ZDHHC12* knockdown (Figure 2A and Supplemental Figure 1C), whereas *ZDHHC12* knockdown had no effect on cGAS/STING-mediated type I IFN activation (Supplemental Figure 1D). Based on the observation that ZDHHC12 knockdown specifically inhibits RIG-I-N–mediated type I IFN activation, along with the established role of MAVS palmitoylation in RLR signaling activation demonstrated in figure 1, L–M, we speculated that ZDHHC12 may play an important role in mediating the palmitoylation of MAVS. Further experiments revealed that ectopic expression of WT ZDHHC12 rather than its enzymatically inactive C127S mutant increased the level of MAVS palmitoylation, indicating that the catalytic activity of ZDHHC12 was required for MAVS palmitoylation (Figure 2, B and C). In agreement with these results, ZDHHC12 could not further catalyze the palmitoylation of MAVS C79S or MAVS C79F (Figure 2, D–G). Furthermore, *ZDHHC12* depletion impaired SeV-triggered palmitoylation of endogenous MAVS in both THP-1–derived macrophages and BMDMs (Figure 2, H–K). Currently, ZDHHC4 has been shown to functionally affect the palmitoylation of MAVS in murine B16 melanoma cells (23). To investigate the roles of ZDHHC12 and ZDHHC4 in the palmitoylation of MAVS following SeV infection or IC poly(I:C) treatment in macrophages, we conducted knockdown experiments in which *ZDHHC12* or *ZDHHC4* was targeted. *ZDHHC12* knockdown substantially reduced the level of MAVS in THP-1–derived macrophages and BMDMs following SeV infection or IC poly(I:C) treatment (Supplemental Figure 2). In contrast, *ZDHHC4* knockdown had a minor effect on the palmitoylation level of MAVS under the indicated conditions (Supplemental Figure 2). These data suggest that ZDHHC12 functions as a major palmitoyl *S*-acyltransferase that mediates MAVS palmitoylation upon RNA virus infection in macrophages.

### ZDHHC12 interacts with MAVS.

**Figure 3:**
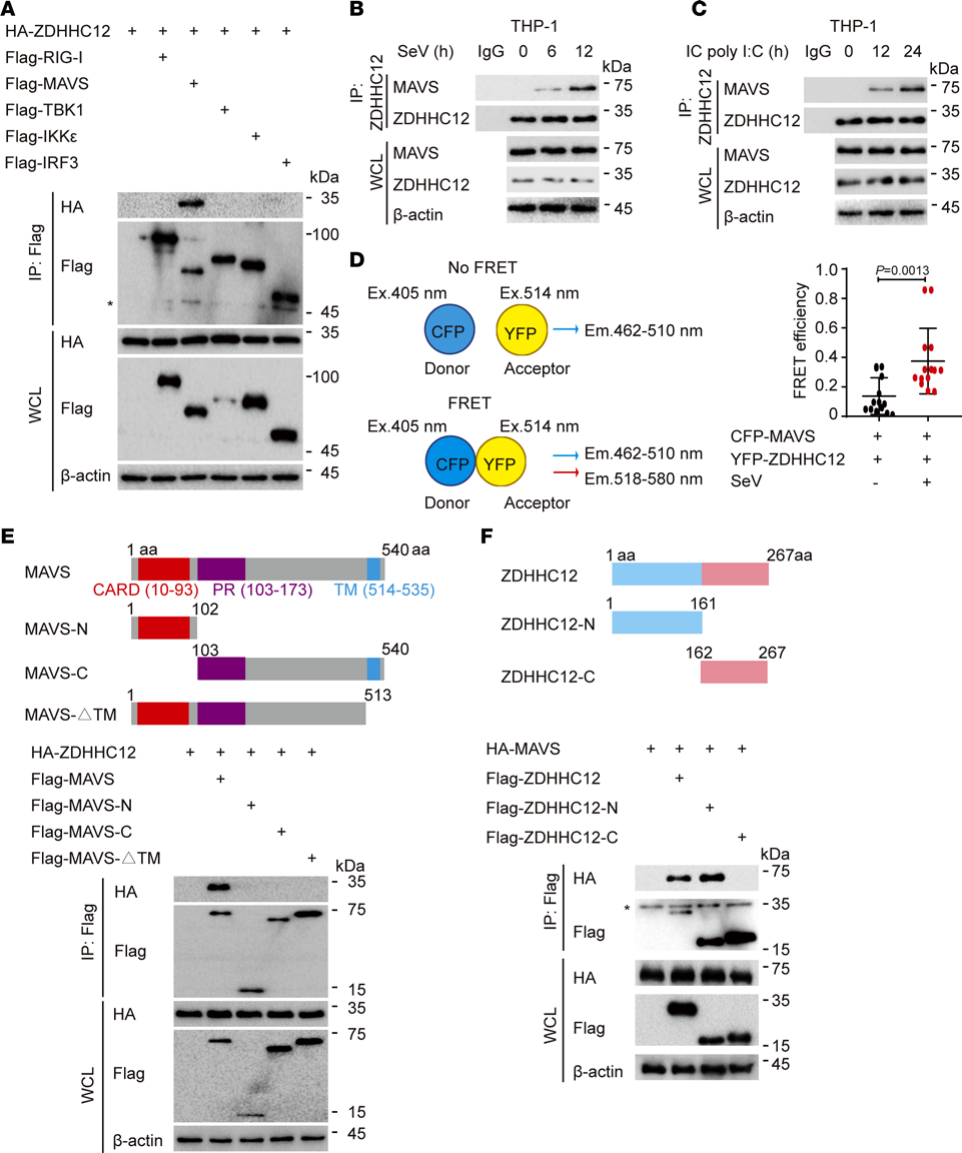
ZDHHC12 associates with MAVS. (**A**) HEK293T cells were transfected with HA-ZDHHC12, together with indicated plasmids, respectively, for 24 hours. Cell extracts were collected for immunoprecipitation (IP) and immunoblot analysis. WCL, whole-cell lysates. Asterisks indicate nonspecific anti-FLAG reactive bands. (**B** and **C**) THP-1–derived macrophages were treated with SeV (MOI = 1) (**B**) or IC poly(I:C) (5 μg/mL) (**C**) for indicated time periods. Cell lysates were collected for IP and immunoblot analysis to detect the endogenous MAVS-ZDHHC12 association. (**D**) HEK293T cells were transfected with plasmids expressing CFP-MAVS as a fluorescence resonance energy transfer (FRET) donor and YFP-ZDHHC12 as a FRET acceptor for 24 hours, followed by infection of SeV (MOI = 1) for 12 hours. The interaction between MAVS and ZDHHC12 was determined by a FRET-based protein-protein interaction assay. (**E**) Top: Domain organization of MAVS protein. Bottom: IP and immunoblot analysis of extracts of HEK293T cells transfected with plasmids encoding HA-ZDHHC12 together with indicated plasmids for 24 hours. (**F**) Top: Domain organization of ZDHHC12 protein. Bottom: IP and immunoblot analysis of extracts of HEK293T cells transfected with plasmids encoding HA-MAVS together with indicated plasmids for 24 hours. Asterisks indicate nonspecific anti-FLAG reactive bands. In **A**–**C**, **E**, and **F**, similar results were obtained for 3 independent experiments. In **D**, data are presented as mean values ± SD. Statistical analysis was performed using 2-tailed Student’s *t* test.

Given that ZDHHC12 is responsible for mediating MAVS palmitoylation in macrophages, we next determined whether ZDHHC12 interacts with MAVS. Immunoprecipitation assays revealed that ZDHHC12 could specifically bind to MAVS but not other molecules involved in RLR signaling (Figure 3A). To determine the physiological relevance of these findings, we treated THP-1–derived macrophages with SeV or IC poly(I:C). We observed that ZDHHC12 barely interacted with MAVS without the indicated stimulation. However, the ZDHHC12-MAVS interaction was increased in a time-dependent manner with SeV infection or IC poly(I:C) treatment (Figure 3, B and C), which was consistent with the dynamic pattern of MAVS palmitoylation under stimulation. A fluorescence resonance energy transfer (FRET) assay further confirmed that there was a strong interaction between MAVS and ZDHHC12 upon viral infection (Figure 3D). MAVS consists of 3 domains: an N-terminal CARD domain, a middle proline-rich region, and a C-terminal transmembrane (TM) domain (Figure 3E). To determine which domain of MAVS is needed for the interaction between ZDHHC12 and MAVS, we generated several deletion constructs of MAVS and observed that ZDHHC12 could interact with full-length MAVS but not with MAVS (N) (aa 1–102), MAVS (C) (aa 103–540), or the MAVS ΔTM (aa 1–513) domain (Figure 3E), suggesting that the integrity of MAVS is critical for its ability to bind with ZDHHC12. Furthermore, to map the essential domains of ZDHHC12 that mediate its association with MAVS, we generated ZDHHC12 truncations and found that the N-terminal domain of ZDHHC12 is important for the ZDHHC12-MAVS association (Figure 3F). Taken together, these results suggest that ZDHHC12 interacts with MAVS to mediate its palmitoylation upon RNA virus infection in macrophages.

### ZDHHC12 is pivotal for cellular antiviral responses against RNA viruses.

We next sought to determine the biological role of ZDHHC12 in antiviral innate immunity. Upon ectopic ZDHHC12 overexpression in HEK293T cells, SeV-induced, vesicular stomatitis virus–induced (VSV-induced), or IC poly(I:C)–induced IFN-β and ISRE luciferase reporter activity was increased in a dose-dependent manner (Supplemental Figure 3A). Moreover, ectopic ZDHHC12 overexpression increased the phosphorylation of TBK1 and IRF3 upon SeV infection (Supplemental Figure 3B). Furthermore, we infected HEK293T cells that ectopically expressed ZDHHC12 with VSV-eGFP and observed that ZDHHC12 overexpression substantially inhibited viral replication compared with that in cells expressing the empty vector through fluorescence microscopy and flow cytometry analysis (Supplemental Figure 3, C and D).

**Figure 4:**
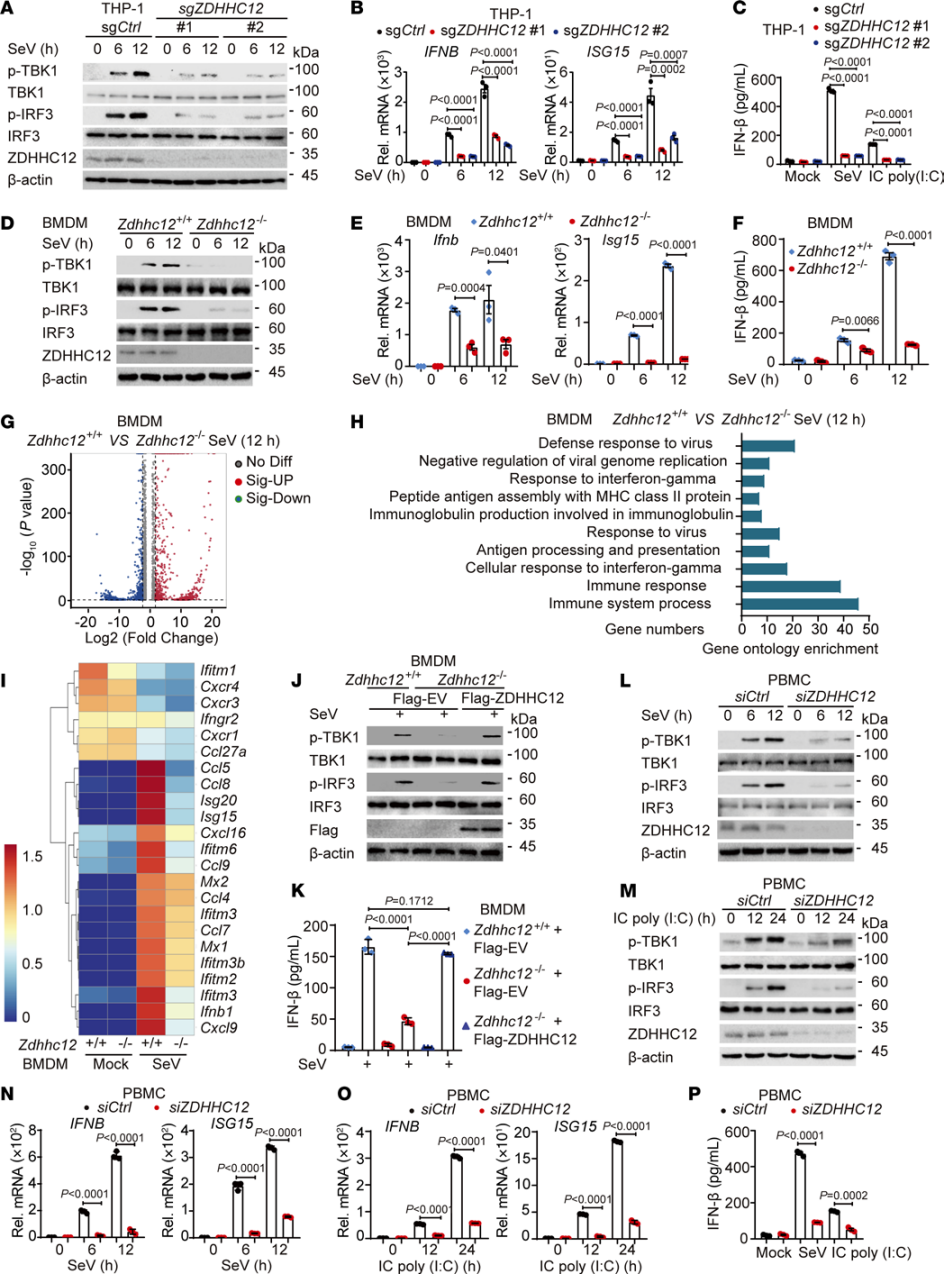
*ZDHHC12* deficiency impairs cellular antiviral responses. (**A** and **B**) WT (sg*Ctrl*) or *ZDHHC12*-KO (sg*ZDHHC12*#1/#2) THP-1 macrophages were treated with SeV for indicated time periods. Cell lysates were collected for immunoblot and real-time qPCR analysis. (**C**) WT or *ZDHHC12*-KO THP-1 macrophages were treated with SeV or IC poly(I:C). IFN-β release was determined by ELISA. (**D**–**F**) BMDMs isolated from *Zdhhc12^+/+^* or *Zdhhc12^–/–^* mice were treated with SeV for indicated time periods. Cell lysates were collected for immunoblot (**D**) and real-time qPCR analysis (**E**). IFN-β release was determined by ELISA (**F**). (**G**) Volcano plot of differentially expressed genes in BMDMs isolated from *Zdhhc12^+/+^* or *Zdhhc12^–/–^* mice with SeV infection (MOI = 1, 12 hours). (**H**) Gene Ontology enrichment analysis of the genes in **G**. (**I**) Heatmap view of mRNA variations of type I IFN–mediated ISG sets in BMDMs from *Zdhhc12^+/+^* or *Zdhhc12^–/–^* mice with SeV infection. (**J** and **K**) Immunoblot (**J**) and ELISA analysis (**K**) in *Zdhhc12^+/+^* or *Zdhhc12^–/–^* BMDMs transfected with empty vector (EV) or plasmid encoding ZDHHC12 using jetPRIME for 48 hours, followed by infection with SeV for 12 hours. (**L**–**O**) The human PBMCs were transfected with si*Ctrl* or si*ZDHHC12* for 48 hours and infected with SeV or IC poly(I:C) for indicated time periods. Cell lysates were collected for immunoblot (**L** and **M**) and real-time qPCR analysis (**N** and **O**). (**P**) WT or *ZDHHC12*-knockdown PBMCs were treated with SeV or IC poly(I:C). IFN-β release was determined by ELISA. In **A**, **D**, **G**–**J**, **L**, and **M**, similar results were obtained for 3 independent experiments. In **B**, **C**, **E**, **F**, **K**, **O**, and **P**, data are presented as mean values ± SEM. Statistical analysis was performed using 2-tailed Student’s *t* test in **E**, **F**, **O**, and **P** or 1-way ANOVA multiple comparisons in **B**, **C**, and **K**.

To further elucidate the functions of ZDHHC12 in antiviral innate immune signaling, we designed two *ZDHHC12*-specific small interfering RNAs (siRNAs) to downregulate the expression of *ZDHHC12*. *ZDHHC12* knockdown apparently decreased the phosphorylation of TBK1 and IRF3 as well as the expression of *IFNB* and *ISG15* mRNAs induced by SeV but not that induced by the DNA virus herpes simplex virus type 1 (HSV-1) (Supplemental Figure 3, E–H), indicating that ZDHHC12 specifically regulated type I IFN signaling mediated by RLRs but not DNA sensors. To substantiate these findings, we used the CRISPR/Cas9 system to generate *ZDHHC12*-KO THP-1 cells. In comparison with the WT group, *ZDHHC12* deficiency markedly inhibited the phosphorylation of TBK1 and IRF3, the expression of *IFNB* and *ISG15* mRNAs, and the IFN-β secretion induced by SeV infection or IC poly(I:C) treatment (Figure 4, A–C, and Supplemental Figure 3, I and J). We next investigated whether ZDHHC12 regulates the type I IFN signaling pathway via its enzymatic activity and found that mutation of the ZDHHC12 enzymatic site abrogated its ability to upregulate type I IFN signaling during viral infection (Supplemental Figure 3, K and L).

To further elucidate the biological importance of ZDHHC12 in RNA virus–triggered innate immune signaling, we generated *Zdhhc12*-deficient mice via CRISPR/Cas9–mediated genome editing as described previously (18). We treated BMDMs with SeV or poly(I:C) and found that *Zdhhc12* deficiency markedly suppressed the phosphorylation of TBK1 and IRF3, the expression of *Ifnb* and *Isg15* mRNAs, and the secretion of IFN-β (Figure 4, D–F, and Supplemental Figure 4, A-C). Global RNA sequencing analysis was subsequently performed to identify genes and pathways regulated by ZDHHC12. Gene Ontology enrichment analysis showed obvious enrichment of immune response functions, including “response to virus” (Figure 4, G and H). The abolishment of *Zdhhc12* led to the downregulation of a variety of ISGs upon viral infection (Figure 4I). Moreover, gene set enrichment analysis (GSEA) revealed that the core enriched genes related to the immune response, immune effector process, and positive immune response were apparently decreased in SeV-infected *Zdhhc12^–/–^* BMDMs (Supplemental Figure 4D). The reintroduction of ZDHHC12 into *Zdhhc12*-deficient BMDMs restored their ability to activate the type I IFN signaling pathway (Figure 4, J and K). Notably, ZDHHC4 has been demonstrated to enhance MAVS-mediated immune responses in murine B16 melanoma cells (23). In our study, *ZDHHC12* knockdown apparently suppressed the expression of *IFNB* and *ISG15* mRNAs triggered by SeV infection or IC poly(I:C) treatment in THP-1–derived macrophages and BMDMs, whereas *ZDHHC4* knockdown only slightly affected the expression of these mRNAs under the indicated conditions (Supplemental Figure 5), indicating that ZDHHC12, but not ZDHHC4, plays a major role in the activation of the RNA virus–mediated type I IFN signaling pathway in macrophages. To further determine the role of ZDHHC12 under physiological conditions, we knocked down *ZDHHC12* in human peripheral blood mononuclear cells (PBMCs) and found that *ZDHHC12* deficiency in PBMCs exhibited a similar pattern to that observed in human and murine macrophages (Figure 4, L–P). Collectively, these results suggest that ZDHHC12 is a positive regulator of type I IFN signaling in response to RNA virus infection in macrophages and PBMCs.

### ZDHHC12-mediated palmitoylation of MAVS does not affect its stability or mitochondrial localization.

Based on our previous studies (18), we investigated whether ZDHHC12-mediated MAVS palmitoylation influences the protein stability of MAVS. Intriguingly, the degradation rate of the MAVS C79S mutant was comparable to that of WT MAVS (Supplemental Figure 6A). In addition, we found that ZDHHC12 overexpression had no discernible influence on the degradation rate of endogenous MAVS (Supplemental Figure 6B). Consistently, *ZDHCC12* deficiency also did not affect the degradation rate of MAVS in THP-1–derived macrophages or BMDMs (Supplemental Figure 6, C and D). Previous studies have indicated that palmitoylation can regulate protein localization (24) and that the mitochondrial localization of MAVS is vital for its aggregation and activation (25). We assessed MAVS-mitochondrion associations using a mitochondrial isolation assay, which revealed no significant differences in the protein levels of WT MAVS and the palmitoylation-deficient mutant MAVS C79S in the mitochondria (Supplemental Figure 6E). Similarly, *ZDHHC12* deficiency had no apparent effect on the protein level of MAVS within the mitochondrial compartment of THP-1–derived macrophages or BMDMs (Supplemental Figure 6, F and G). Taken together, our data suggest that ZDHHC12-mediated MAVS palmitoylation does not affect the stability or mitochondrial localization of MAVS in macrophages.

### ZDHHC12-mediated palmitoylation of MAVS functions downstream of its K63-linked ubiquitination.

**Figure 5:**
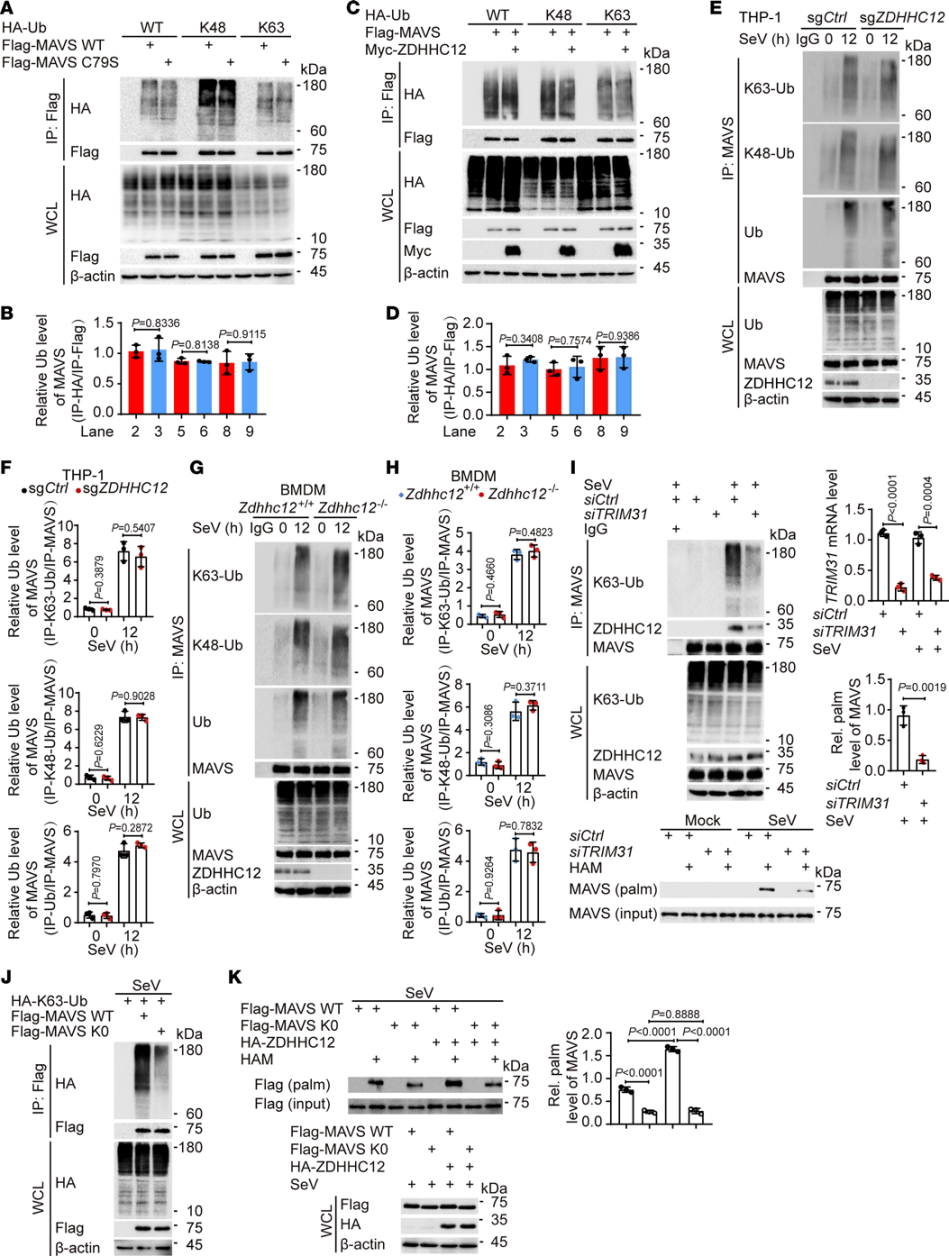
K63 ubiquitination of MAVS facilitates its palmitoylation. (**A** and **B**) HEK293T cells were transfected with indicated plasmids for 24 hours. Cell lysates were collected for IP and immunoblot analysis. (**C** and **D**) HEK293T cells were transfected with indicated plasmids for 24 hours. Cell lysates were collected for IP and immunoblot analysis. (**E**–**H**) IP and immunoblot analysis of WT (sg*Ctrl*) or *ZDHHC12*-KO (sg*ZDHHC12*) THP-1 macrophages (**E** and **F**) or BMDMs from *Zdhhc12^+/+^* or *Zdhhc12^–/–^* mice (**G** and **H**) infected with SeV for 12 hours. (**I**) THP-1–derived macrophages were transfected with si*Ctrl* or si*TRIM31* for 48 hours, and then infected with SeV for 12 hours. Cell lysates were collected for immunoblot analysis, real-time qPCR analysis, IP analysis, and ABE assay. (**J**) HEK293T cells were transfected with indicated plasmids for 24 hours, followed by infection of SeV for 12 hours. Cell extracts were collected for IP and immunoblot analysis. (**K**) HEK293T cells were transfected with WT FLAG-MAVS or FLAG-MAVS K0 mutant in the presence of HA-ZDHHC12 or not for 24 hours, then infected with SeV for 12 hours. Cell lysates were collected for ABE assay and immunoblot analysis. In **A**–**K**, similar results were obtained for 3 independent experiments. In **B**, **D**, **F**, **H**, **I**, and **K**, data are presented as mean values ± SD. Statistical analysis was performed using 2-tailed Student’s *t* test in **B**, **D**, **F**, **H**, and **I** or 1-way ANOVA multiple comparisons in **K**.

Upon viral infection, MAVS undergoes ubiquitination, which is a critical event for regulating MAVS aggregation (12). Considering that other reported posttranslational modifications (PTMs), including acetylation (6), arginine monomethylation (26), *O*-GlcNAcylation (27, 28), and SUMOylation (29), could affect the ubiquitination of MAVS, we speculated that palmitoylation could also influence the ubiquitination of MAVS. To our surprise, mutation of the MAVS palmitoylation site, ZDHHC12 overexpression, or *ZDHHC12* deletion had no discernible influence on MAVS ubiquitination, including K48-linked and K63-linked ubiquitination (Figure 5, A–H), suggesting that ZDHHC12-mediated palmitoylation of MAVS had no obvious effect on its ubiquitination.

Given that K48-linked ubiquitination of MAVS typically triggers its degradation through the ubiquitin-proteasome pathway (30), our findings suggest that ZDHHC12-mediated MAVS palmitoylation does not affect its stability. This observation aligns with the conclusions drawn in Supplemental Figure 6, A–D. Since MAVS C79S–mediated type I IFN signaling has rarely been detected, we speculated that the palmitoylation of MAVS functions downstream of the K63-linked ubiquitination of MAVS. Interestingly, our observations revealed that the knockdown of *TRIM31*, a known mediator of the essential K63-linked ubiquitination of MAVS for its activation (13), led to a significant reduction in the ZDHHC12-MAVS association and the palmitoylation level of MAVS (Figure 5I). These findings suggest that ZDHHC12-mediated MAVS palmitoylation might occur downstream of its K63-linked ubiquitination. To confirm that the K63-linked polyubiquitin of MAVS facilitates its palmitoylation, we replaced all lysine residues in MAVS with arginine to construct the MAVS K0 mutant according to a previous report (31). As expected, MAVS K0 strongly inhibited the K63-linked ubiquitination of MAVS (Figure 5J). Notably, the palmitoylation level of MAVS K0 was substantially decreased, and ZDHHC12 overexpression did not further increase MAVS K0 palmitoylation (Figure 5K). Overall, we propose that K63-linked ubiquitination plays a critical role in recruiting ZDHHC12 to MAVS, which subsequently facilitates its palmitoylation in macrophages.

### Palmitoylation of MAVS is essential for its aggregation.

**Figure 6:**
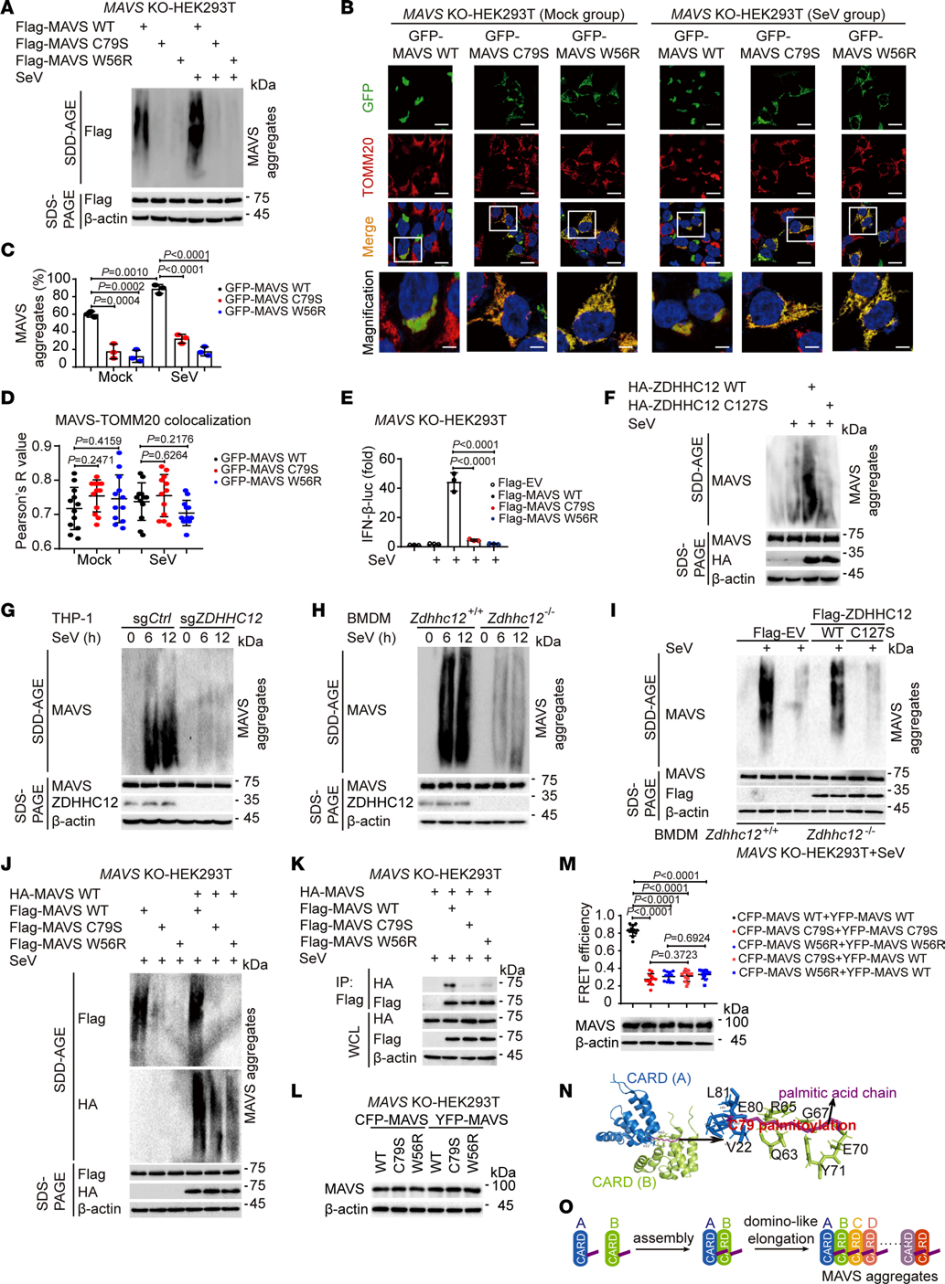
Palmitoylation is essential for MAVS aggregation. (**A**) Immunoblot and SDD-AGE analysis in *MAVS*-KO HEK293T cells transfected with indicated plasmids and infected with SeV. (**B**) The colocalization between MAVS and TOMM20 was examined by confocal microscopy. Scale bars: 10 μm. (**C**) Quantification of MAVS aggregates of **B** (30 cells per sample). (**D**) The MAVS-TOMM20 colocalization was quantified by Pearson’s correlation coefficient. (**E** and **F**) *MAVS*-KO (**E**) or WT (**F**) HEK293T cells were transfected with indicated plasmids, followed by SeV infection. Cell lysates were collected for luciferase reporter assays (**E**) and SDD-AGE analysis (**F**). (**G** and **H**) The indicated cells were infected with SeV for indicated time periods. Cell lysates were collected for immunoblot analysis and SDD-AGE analysis. (**I**) Immunoblot analysis and SDD-AGE analysis in indicated BMDMs transfected with indicated plasmids, followed by infection with SeV. (**J** and **K**) *MAVS*-KO HEK293T cells were transfected with indicated plasmids, then infected with SeV. Cell extracts were harvested for SDD-AGE (**J**) and IP analysis (**K**). (**L** and **M**) *MAVS*-KO HEK293T cells were transfected with the indicated plasmids, and then infected without (**L**) or with SeV (**M**). The MAVS-MAVS interaction was determined by FRET assay (**M**). Cell lysates were collected for immunoblot analysis. (**N**) PyMOL software (https://www.pymol.org/) was used to determine the interaction locations of monomer MAVS CARD (A and B) and the palmitoylation site MAVS C79 in monomer MAVS CARD (A). (**O**) Cartoon of potential cooperativity between palmitoylation of MAVS CARD domain during growth of the MAVS prion-like aggregates. In **A** and **F**–**L**, 3 independent experiments yielded similar results. In **C**, **D**, and **M**, data are presented as mean values ± SD. In **E**, data are presented as mean values ± SEM. Statistical analysis was performed using 1-way ANOVA multiple comparisons in **C**–**E** and **M**.

Given that the palmitoylation of MAVS occurs downstream of its K63-linked ubiquitination, we sought to determine whether MAVS palmitoylation affects its aggregation. We observed that the overexpression of WT MAVS in *MAVS*-KO HEK293T cells led to its aggregation even in the absence of SeV infection, with SeV infection further enhancing this aggregation (Figure 6A), which is consistent with previous findings (13, 32). In the absence or presence of SeV infection, the aggregates of WT MAVS were more abundant than those of palmitoylation-deficient mutant MAVS C79S or polymerization-deficient mutant MAVS W56R (as a negative control, abrogation of the hydrophobic interactions at the intra-strand interface of MAVS W56R prevents MAVS CARD oligomerization; ref. 33) (Figure 6A). Previous studies have indicated that cells with MAVS aggregation exhibit decreased mitochondrial skeleton length and cytosolic area (32, 34–37). According to these criteria, MAVS aggregation can be quantified. Notably, the transfection of WT MAVS in *MAVS*-KO HEK293T cells, with or without SeV infection, led to more significant MAVS aggregation than did the transfection of MAVS C79S or MAVS W56R (Figure 6, B and C), which is in line with the phenomenon depicted in Figure 6A. Furthermore, there were no statistically significant differences in the Pearson’s correlation coefficients derived from the colocalization analysis of either WT MAVS or MAVS C79S with mitochondria, further confirming that palmitoylation of MAVS does not impact its mitochondrial localization (Figure 6D). Consistent with the loss of MAVS aggregation, MAVS C79S or MAVS W56R was almost unable to induce IFN-β reporter activation with SeV infection (Figure 6E). We next observed that ZDHHC12 transfection resulted in greater formation of MAVS aggregates than did transfection with the empty vector or the enzymatically inactive mutant ZDHHC12 C127S (Figure 6F). In accordance with the observation of ZDHHC12 overexpression, *ZDHHC12-*KO THP-1–derived macrophages or *Zdhhc12^–/–^* BMDMs displayed markedly decreased MAVS aggregation compared with that of the indicated control cells upon SeV infection (Figure 6, G and H). We found that MAVS aggregation was dramatically greater in WT ZDHHC12 BMDMs, but not in ZDHHC12 C127S–reconstituted *Zdhhc12^–/–^* BMDMs, upon SeV infection than in control *Zdhhc12^–/–^* BMDMs (Figure 6I). Collectively, these data demonstrate that ZDHHC12-mediated palmitoylation of MAVS is essential for its aggregation during RNA virus infection.

Previous studies demonstrated that active MAVS fibers could catalyze similar biochemical changes in inactive MAVS (9, 38). Hence, we sought to investigate whether normal MAVS (WT MAVS) could trigger the aggregation of palmitoylation-deficient MAVS. We found that the palmitoylation-deficient mutant MAVS C79S or the polymerization-deficient mutant MAVS W56R failed to induce MAVS aggregation in the absence or presence of normal MAVS palmitoylation (Figure 6J). Consistently, HA-MAVS barely interacted with FLAG–MAVS C79S or FLAG–MAVS W56R (Figure 6K). These results suggest that palmitoylation at MAVS C79 plays a pivotal role in MAVS aggregation. To further support our conclusion, we constructed CFP-MAVS and GFP-MAVS plasmids to perform FRET assay to assess the MAVS-MAVS association (Figure 6L). Our findings revealed that the FRET efficiency in the CFP-MAVS C79S/YFP-MAVS C79S and CFP-MAVS W56R/YFP-MAVS W56R groups was substantially lower than that in the CFP-MAVS WT/YFP-MAVS WT group (Figure 6M), indicating that the interactions of MAVS C79S–MAVS C79S and MAVS W56R–MAVS W56R were reduced, which is consistent with the observations depicted in Figure 6, A–C. In addition, the FRET efficiency in the CFP-MAVS C79S/YFP-MAVS WT and CFP-MAVS W56R/YFP-MAVS WT groups was lower than that in the CFP-MAVS WT/YFP-MAVS WT group (Figure 6M), suggesting a decreased association between WT MAVS and its mutations, which is in line with the findings presented in Figure 6K. Notably, the FRET efficiency in the CFP-MAVS C79S/YFP-MAVS WT group did not differ statistically from that in the CFP-MAVS C79S/YFP-MAVS C79S group (Figure 6M), which aligns with the observations illustrated in Figure 6J, suggesting that the palmitoylation of MAVS is essential for MAVS oligomerization. Based on previous structural analysis (33), we used the MAVS CARD (A)–CARD (B) 1:1 complex as the initiating template and found that the palmitic acid chain introduced at C79 of the MAVS CARD (A) could reach the pocket of the MAVS CARD (B) to tighten the MAVS CARD (A)–CARD (B) association (Figure 6N). Therefore, based on our observations and structural analysis, we speculate that MAVS palmitoylation might trigger a domino-like effect for MAVS aggregation (Figure 6O). Collectively, these data suggest that palmitoylation at MAVS C79 is responsible for MAVS aggregation.

### ZDHHC12 deficiency dampens antiviral immunity in vivo.

**Figure 7:**
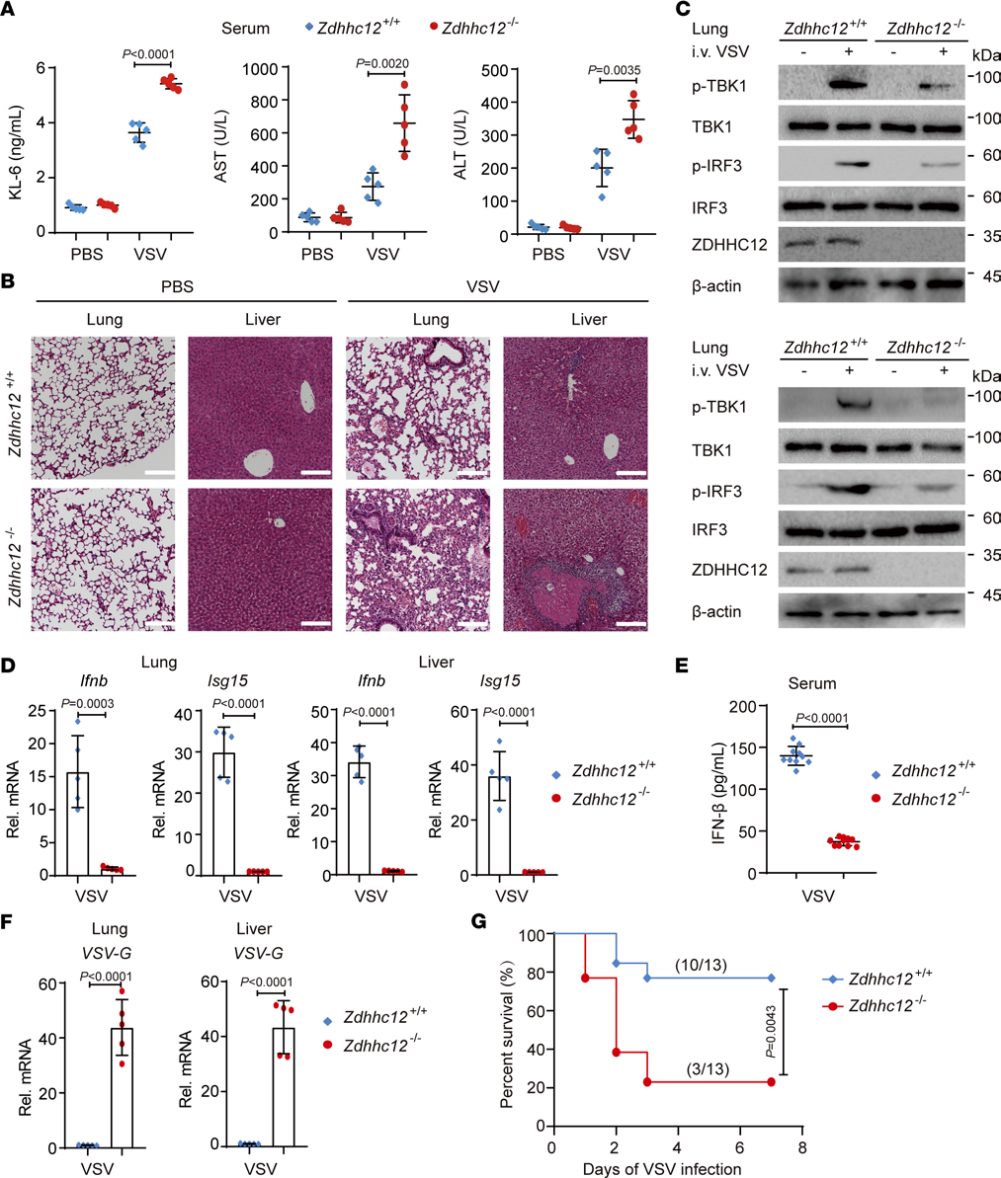
*Zdhhc12* deficiency inhibits antiviral immunity against RNA viruses in vivo. (**A**–**E**) *Zdhhc12^+/+^* or *Zdhhc12^–/–^* mice (*n* = 5 per group) were intravenously (i.v.) injected with PBS or VSV (1 × 10^8^ PFU/mouse) for 24 hours. (**A**) The production of KL-6, ALT, and AST in serum of indicated mice was determined by ELISA. (**B**) The lung and liver tissues were isolated and stained with H&E, and assayed using a light microscope. Scale bars: 100 μm. (**C**) The phosphorylation levels of TBK1 and IRF3 of the lung or liver tissues (the mixture of 5 mice per group) were detected by immunoblot analysis. (**D**) *Ifnb* and *Isg15* mRNA levels of the lung or liver tissues of VSV-infected mice were detected by real-time qPCR analysis. (**E**) *Zdhhc12^+/+^* or *Zdhhc12^–/–^* mice (*n* = 10 per group) were intravenously injected with VSV (1 × 10^8^ PFU/mouse) for 24 hours. The IFN-β production of serum was determined by ELISA. (**F**) The *VSV-G* mRNA level of the lung or liver tissues of VSV-infected mice in **A**. (**G**) Survival curve of *Zdhhc12^+/+^* or *Zdhhc12^–/–^* mice (*n* = 13 per group) intravenously injected with VSV (1 × 10^8^ PFU/mouse). Difference was calculated using log-rank (Mantel-Cox) test. In **A** and **D**–**F**, each symbol represents an individual mouse. Data are presented as mean values ± SD. Statistical analysis was performed using 2-tailed Student’s *t* test.

To investigate the functional significance of ZDHHC12 in the host antiviral response in vivo, we challenged *Zdhhc12^+/+^* and *Zdhhc12^–/–^* mice with VSV. To assess the extent of lung and liver injury induced by VSV, we evaluated histological and biochemical changes in mice with or without VSV infection, which has been commonly employed in other studies (39–45). Krebs von den lungen 6 (KL-6) is expressed predominantly on alveolar type II cells in the lungs and serves as a specific marker for lung injury, with elevated serum KL-6 levels indicating such damage (46–48). Alanine transaminase (ALT) and aspartate aminotransferase (AST) are present primarily within liver cells and act as indicators of liver damage, with increased serum ALT and AST levels indicating injury. We observed higher serum KL-6, ALT, and AST levels in *Zdhhc12^–/–^* mice infected with VSV than in *Zdhhc12^+/+^* mice (Figure 7A). Histological changes in the lung and liver, as indicated by hematoxylin and eosin (H&E) staining (Figure 7B), were consistent with fluctuations in the serum KL-6, ALT, and AST concentrations. Together, these results indicate greater injury to the lungs and livers of *Zdhhc12^–/–^* mice infected with VSV. Upon VSV infection, the levels of phosphorylated TBK1 and IRF3, as well as the expression levels of *Ifnb* and *Isg15* in the liver and lung tissues of *Zdhhc12^–/–^* mice, were markedly lower than those in the organs of *Zdhhc12^+/+^* mice (Figure 7, C and D). In addition, we observed less IFN-β in the serum of *Zdhhc12^–/–^* mice infected with VSV than in that of *Zdhhc12^+/+^* mice (Figure 7E). Consistent with the decreased production of IFN-β, *Zdhhc12^–/–^* mice presented higher *VSV-G* mRNA expression levels in liver and lung tissues than did *Zdhhc12^+/+^* mice in response to VSV infection (Figure 7F). Moreover, *Zdhhc12^–/–^* mice were more susceptible to VSV infection than were *Zdhhc12^+/+^* mice (Figure 7G). Taken together, these results demonstrate that *Zdhhc12* deficiency impairs innate antiviral responses against RNA virus infection by decreasing the production of IFN-β in vivo.

To investigate the role of ZDHHC12 from bone marrow–derived cells in innate antiviral immune responses in vivo, we generated chimeric mice through adoptive bone marrow transplantation, as previously described (49, 50). Among bone marrow recipients that were infected with VSV, WT mice receiving *Zdhhc12*-KO bone marrow (referred to as KO>WT) presented greater levels of lung and liver injury than did WT mice receiving WT bone marrow (referred to as WT>WT): the serum KL-6, ALT, and AST concentrations from KO>WT mice were higher than those from WT>WT mice (Supplemental Figure 7A); and histological changes in the lungs and liver, as indicated by H&E staining, aligned with fluctuations in the serum KL-6, ALT, and AST concentrations (Supplemental Figure 7B). Upon VSV infection, the levels of phosphorylated TBK1 and IRF3 in the liver and lung tissues of KO>WT mice were lower than those in the organs of WT>WT mice (Supplemental Figure 7C). ELISA analysis showed less IFN-β in the serum of KO>WT mice infected with VSV than in that of WT>WT mice (Supplemental Figure 7D). Furthermore, compared with WT>WT mice, KO>WT mice were more susceptible to VSV infection according to overall survival assays (Supplemental Figure 7E). Collectively, these results highlight a crucial role for ZDHHC12-regulated innate antiviral responses in protection against RNA virus infection in bone marrow–derived cells.

## Discussion

MAVS functions as a critical “switch” in the RNA virus–mediated antiviral innate signaling pathway via the formation of prion-like aggregates upon receiving upstream signals from the cytosolic RNA sensor RIG-I (51, 52). Accumulating evidence has demonstrated the pivotal roles of MAVS in multiple biological processes, including pathogen invasion and autoimmune diseases (e.g., SLE and psoriasis) (22, 53, 54). Hence, MAVS activation must be activated in a timely manner to establish effective immune responses to pathogen challenge and then turned off to maintain immune homeostasis. Many studies have revealed that combined PTMs of MAVS — e.g., ubiquitination (13, 55, 56), arginine monomethylation (26), *O*-GlcNAcylation (27, 28), and SUMOylation (29) — are crucial to ensure an “on” or “off” switch for regulating MAVS aggregation through affecting its K48- or K63-linked ubiquitination. In our study, we demonstrate that palmitoylation is essential for MAVS aggregation by functioning downstream of its K63-linked ubiquitination rather than directly influencing the ubiquitination process in macrophages. Without palmitoylation, MAVS might not exist as an active monomer for subsequent aggregation.

Previous studies have demonstrated that palmitoylation catalyzed by ZDHHCs plays important roles in modulating innate immune responses (57, 58). For example, ZDHHC5-mediated NOD2 palmitoylation is crucial for facilitating antimicrobial and proinflammatory responses by redistributing NOD2 localization to plasma membranes and endosomal components. Additionally, it restricts the autophagic degradation of NOD2, mediated by the cargo recognition receptor SQSTM1/p62, thereby enhance NOD2 stability (14, 21). ZDHHC18-mediated cGAS palmitoylation functions as a brake to shut down cGAS signaling by causing conformational changes in cGAS to inhibit cGAS-DNA binding and dimerization in macrophages (17). Collectively, these studies indicate that palmitoylation can regulate protein stability, subcellular localization, or conformational state. MAVS aggregation in the mitochondria is required for its activation and RLR-mediated antiviral innate immune responses (51). However, reports on the regulation of MAVS aggregation by palmitoylation are limited. In our study, we demonstrate that endogenous MAVS is barely palmitoylated in the resting state, whereas MAVS palmitoylation occurs upon RNA virus infection in macrophages. ZDHHC12 is a major palmitoyl *S*-acyltransferase that mediates MAVS palmitoylation after RNA virus infection in human and murine macrophages. Currently, ZDHHC4 has been shown to functionally affect the palmitoylation and activation of MAVS in the murine B16 melanoma cell line (23). We compared the functions of ZDHHC4 and ZDHHC12 in macrophages and demonstrated that deficiency of *ZDHHC12*, but not *ZDHHC4*, notably decreased the palmitoylation levels of MAVS and the RNA virus–triggered type I IFN signaling pathway in THP-1–derived macrophages and BMDMs after SeV infection or IC poly(I:C) treatment. Notably, a very low level of MAVS palmitoylation was still detected in *ZDHHC12*-deficient cells, indicating that other ZDHHCs (e.g., ZDHHC4) may contribute slightly to MAVS palmitoylation in macrophages. Previous studies have revealed the divergent roles of ZDHHC9 and ZDHHC18 in the regulation of cGAS-mediated immune responses. Specifically, ZDHHC9 has been demonstrated to enhance cGAS-mediated immune responses in the human breast cancer cell line MDA-MB-231 (15), whereas ZDHHC18 exerts a negative regulatory influence on cGAS-mediated immune responses in both human and murine macrophages (17). Thus, our collaborative research with W. Sheng’s team has shed light on the functional roles of ZDHHC12 in human and murine macrophages and ZDHHC4 in murine B16 melanoma cells, revealing their ability to promote MAVS-mediated immune responses. These findings suggest that within immune or cancer cell contexts, distinct palmitoyl acyltransferases may modulate immune responses through distinct mechanisms.

We discovered in our previous study that ZDHHC12-mediated NLRP3 palmitoylation prevents overt inflammasome activation by promoting NLRP3 degradation (18). However, in this study, ZDHHC12-mediated MAVS palmitoylation facilitates MAVS aggregation without affecting its stability or mitochondrial localization, suggesting that palmitoylation could play a critical role in regulating the aggregation of membrane-associated proteins. The distinct functions of ZDHHC12-mediated palmitoylation in regulating the NLRP3 inflammasome and MAVS-mediated antiviral innate immunity may help balance immune responses and prevent autoimmune diseases.

Dysregulation of prion-like protein aggregation could lead to the formation of toxic protein aggregates, which are the primary risk factors for most neurodegenerative diseases, including Alzheimer’s disease and Parkinson’s disease (59, 60). Metabolic modifications that modulate the aggregation of prion-like proteins could have significant implications for the development of these diseases. For example, acetylation of tau could affect its ability to bind to microtubules, increase its prion-like aggregation, and decrease its degradation, resulting in synaptic dysfunction and loss (61, 62). Hence, the inhibition of tau acetylation has been suggested as a potential therapeutic strategy for Alzheimer’s disease and other tauopathies. Upon RNA virus infection, the signaling adaptor MAVS forms prion-like aggregates to elicit robust antiviral signaling cascades, but excessive MAVS aggregation leads to host immunopathology (e.g., SLE and psoriasis), indicating that it is critical for uncovering the checkpoint that governs the prion-like behavior of MAVS to maintain immune homeostasis. In our study, we reveal a mechanism by which K63-linked ubiquitin chains on MAVS recruit palmitoyl *S*-acyltransferases (e.g., ZDHHC12) to mediate MAVS palmitoylation, which act as a checkpoint for MAVS aggregation and downstream antiviral innate immunity in macrophages. Moreover, we demonstrate that defective MAVS palmitoylation might contribute to the pathogenesis of MAVS-associated SLE. Collectively, the discovery of the roles of metabolic modifications in regulating the aggregation of prion-like proteins provides new insights into the mechanisms underlying both normal physiological processes and pathological conditions, and further research in this field may lead to the development of novel therapeutic strategies for the treatment of related diseases associated with prion-like protein aggregation.

In summary, we identify MAVS as a palmitoylated protein, and palmitoylation at C79 of MAVS is necessary for its aggregation and the activation of antiviral innate immune responses. We reveal a mechanism that links the functions of ubiquitination and palmitoylation in the regulation of MAVS activity in macrophages. Therefore, this new understanding of MAVS aggregation might provide potential targets for drug development against viral infections and autoimmune diseases.

## Methods

### Sex as a biological variable.

The sex of the mice used in this study was not considered as a biological variable.

### Mice.

C57BL/6 *Zdhhc12^–/–^* mice were generated via CRISPR/Cas9–mediated genome editing by Cyagen Biosciences, as described previously (18). C57BL/6 WT (GDMLAC-07) mice were obtained from Guangzhou Medical Laboratory Animal Center. In all experiments, 6- to 8-week-old mice were used. All the mice were housed in a specific pathogen–free animal facility with standard temperature conditions maintained at 20°C–26°C, relative humidity at 40%–70%, and a strict 12-hour light/12-hour dark cycle (lights on at 8 am and off at 8 pm) at Sun Yat-sen University.

### Cell culture and transfection.

The HEK293T cell line purchased from American Type Culture Collection (ATCC) was cultured in Dulbecco’s modified Eagle medium (DMEM; Corning, catalog 10-013-CVR) supplemented with 10% fetal bovine serum (FBS; Gibco, catalog 1099-141) and 1% l-glutamine (Gibco, catalog 25030-081). The *MAVS*-KO HEK293T cell line has been described previously (63). The human monocytic THP-1 cell line obtained from ATCC was cultured in RPMI 1640 medium (Gibco, catalog C22400500BT) supplemented with 10% FBS (Gibco) and 1% l-glutamine (Gibco). Monocytic THP-1 cells maintained in RPMI 1640 medium containing 100 ng/mL phorbol-12-myristate-13-acetate (PMA; Sigma-Aldrich, catalog P1585) for 12 hours were differentiated into macrophages, and the macrophages were then treated with the indicated stimulus after a 24-hour resting period. PBMCs isolated from healthy donors were collected in BD Vacutainer CPT tubes. PBMCs were cultured in RPMI 1640 medium (Gibco) supplemented with 10% FBS (Gibco) and 1% l-glutamine (Gibco). Bone marrow cells were isolated from randomly chosen 6-week-old *Zdhhhc12^+/+^* or *Zdhhc12^–/–^* mice and then differentiated into BMDMs cultured in 10 mL of conditioned medium (DMEM supplemented with 100 ng/mL macrophage colony-stimulating factor [PeproTech, catalog AF-315-02], 1% penicillin-streptomycin [Gibco, catalog 15140122], 10% FBS, and 1% l-glutamine), as described previously. HEK293T cells were transfected with the indicated plasmids or poly(I:C) (5 μg/mL; InvivoGen, catalog tlrl-picw) with Superluminal High-efficiency Transfection Reagent (MIKX, catalog 11231804). THP-1–derived macrophages and BMDMs were transfected with poly(I:C) (5 μg/mL) or the indicated plasmids with Lipofectamine 3000 reagent (Thermo Fisher Scientific, catalog L3000015) and jetPRIME (Polyplus-transfection, catalog 114-01), respectively. All the indicated cells were grown at 37°C in a 5% CO_2_ incubator.

### Reagents and antibodies.

Antibodies specific for TBK1 (1:1,000; catalog 3013), phospho-TBK1 (Ser172) (1:1,000; catalog 5483S), IRF3 (1:1,000; catalog 4302S), phospho-IRF3 (Ser396) (1:1,000; catalog 4947S), ubiquitin (1:1,000; catalog 58395S), ubiquitin K48 (1:1,000; catalog 4289S), and ubiquitin K63 (1:1,000; catalog 5621S) were purchased from Cell Signaling Technology. Horseradish peroxidase (HRP)–anti-FLAG (M2) (1:4,000; catalog A8592), anti–β-actin (1:5,000; catalog A1978), and FLAG beads (catalog A2220) were purchased from Sigma-Aldrich. Anti-MAVS (1:1,000; catalog sc-166583), anti-MAVS (1:1,000; catalog sc-365333), goat anti-rabbit (1:4,000; catalog sc-2004), and goat anti-mouse (1:4,000; catalog sc-2005) antibodies were purchased from Santa Cruz Biotechnology. Anti-TOMM20 (1:1,000; catalog 11802-1-AP) was purchased from the ProteinTech Group. Anti–c-Myc–HRP (1:2,000; catalog 11814150001) and anti-hemagglutinin (anti-HA)–HRP (1:3,000; catalog 12013819001) were purchased from Roche Applied Science. Alexa Fluor 488–conjugated goat anti-mouse (catalog A11029), Alexa Fluor 488–conjugated goat anti-rabbit (catalog A11034), Alexa Fluor 568–conjugated goat anti-mouse (catalog A11031), and Alexa Fluor 568–conjugated goat anti-rabbit (catalog A11036) antibodies were purchased from Invitrogen. Protein A–agarose (catalog 20333) and protein G–agarose (catalog 20399) were obtained from Pierce. EDTA-free protease inhibitor (catalog BL630B) and phosphatase inhibitor (catalog 04906837001) were purchased from Biosharp and Roche Applied Science, respectively.

### Bone marrow transplant.

To perform bone marrow transplantation, recipient mice (8 weeks old) were given antibiotics (gentamicin, 1 mg/mL) in their drinking water for 2 days before being given 950 cGy irradiation. Donor bone marrow cells were obtained from donor mice (6–8 weeks old), and 5 × 10^6^ cells were intravenously injected into the recipient mice (after irradiation), followed by antibiotic-containing drinking water for 1 week. The chimeric mice were housed in a specific pathogen–free animal facility and were used for related experiments 6 weeks later.

### Virus infection.

Sendai virus (SeV) and vesicular stomatitis virus (VSV) were provided by Xiaofeng Qin (Suzhou Institute of Systems Medicine, Suzhou, China). HSV-1 was provided by Guoying Zhou (Guangzhou Medical University, Guangzhou, China). The cells were infected with the indicated viruses at a multiplicity of infection (MOI) of 1. *Zdhhc12^+/+^* mice (8 weeks old), *Zdhhc12^–/–^* mice (8 weeks old), and the indicated chimeric mice (14 weeks old) were intravenously injected with VSV (1 × 10^8^ PFU/mouse). Blood samples were collected from the angular vein at 24 hours after VSV injection, and serum samples were isolated by centrifugation at 500*g* at 4°C for 15 minutes and stored at –80°C until assayed. The concentrations of IFN-β and KL-6 in the serum samples were quantified by ELISA. Additionally, the levels of AST and ALT in the serum samples were assessed by Kingmed Diagnostics. All the mice were euthanized by CO_2_ exposure, and the lung and liver tissues were collected for immunoblotting, quantitative PCR (qPCR), and histological analyses. For the survival experiments, *Zdhhc12^+/+^* mice (8 weeks old), *Zdhhc12^–/–^* mice (8 weeks old), and the indicated chimeric mice (14 weeks old) were monitored for survival after intravenous injection of VSV (1 × 10^8^ PFU/mouse).

### Histological assessment of acute injury.

The indicated mice were euthanized by CO_2_ exposure. The isolated lung and liver tissues were fixed in 4% paraformaldehyde (Meilunbio, catalog MA0192-1), embedded in paraffin, sectioned (thickness, 6 μm), and stained with H&E (Servicebio). The H&E-stained sections were examined by microscopy (DMi8, Leica).

### Small interfering RNA and single-guide RNA transfection.

The chemically synthesized small interfering RNAs (siRNAs) of the target genes were generated by Sangon Biotech. The single-guide RNA (sgRNA) sequences were designed using the CRISPR Design Tool (http://chopchop.cbu.uib.no/), synthesized by Sangon Biotech, and cloned and inserted into the lenti-CRISPR v2 vector (Addgene plasmid 108100) to obtain the indicated gene-targeting vector. The siRNAs and sgRNAs were transfected using Lipofectamine RNAiMAX (Invitrogen, catalog 13778100) and Superluminal High-efficiency Transfection Reagent (MIKX, catalog 11231804), respectively, according to the manufacturers’ instructions. The sequences of the siRNAs and sgRNAs used were as follows: control siRNA, 5′-UUCUCCGAACGUGUCACGUTT-3′; human *ZDHHC12* siRNA #1, 5′-GGUCAGUGGUUGCGGUCCATT-3′; human *ZDHHC12* siRNA #2, 5′-AGGAGGAGCUCAAAGAGGATT-3′; human *TRIM31* siRNA, 5′-GGACCACAAAUCCCAUAAUTT-3′; human *ZDHHC4* siRNA, 5′-CCACCAACCAGACUACUAATT-3′; mouse *Zdhhc12* siRNA, 5′-GGUCUGGCCUUCAGUUCUUTT-3′; mouse *Zdhhc4* siRNA, 5′-GGCUAGUGUAUGCAGAAUATT-3′; *ZDHHC3* sgRNA, 5′-TTGCACGCCCTCATCATGGT-3′; *ZDHHC4* sgRNA, 5′-GAAGTATTTGGCTACTGTC-3′; *ZDHHC5* sgRNA, 5′-ACGGGATTTCACGTGGTTC-3′; *ZDHHC6* sgRNA, 5′-TTAGGAACAACCATAGCTGT-3′; *ZDHHC7* sgRNA, 5′-GTCGCCTATGCAGACTTCG-3′; *ZDHHC8* sgRNA, 5′-GCTCCGCTGTACAAGAACG-3′; *ZDHHC9* sgRNA, 5′-CGCCCGAGGAATCACTCCA-3′; *ZDHHC12* sgRNA, 5′-AGTATCTGCAGCGCCGAAGA-3′; *ZDHHC13* sgRNA, 5′-CAGACCCCACTCTTATTGA-3′; *ZDHHC16* sgRNA, 5′-TGGTGTGTTCGGGCTGGCTT-3′; *ZDHHC17* sgRNA, 5′-GTGCTATTGTGGATCAACT-3′; *ZDHHC18* sgRNA, 5′-CAGACAAGCTTCACCGACCC-3′; *ZDHHC20* sgRNA, 5′-TAGGACCAGACGACCACGA-3′.

### RNA extraction and real-time qPCR.

Total RNA was isolated from the indicated cells or tissues with TRIzol reagent (Invitrogen, catalog 10296010). cDNA was subsequently generated using HiScript III RT SuperMix for qPCR (+gDNA wiper) (Vazyme, catalog R323) according to the manufacturer’s instructions. Real-time qPCR analysis was performed using 2× PolarSignal SYBR Green mix Taq (with Tli RNase H) (MIKX, catalog MKG900). All the data were normalized to the expression of human *GAPDH* or mouse *Gapdh*. The following primers were used: human *IFNB* forward, 5′-CCTACAAAGAAGCAGCAA-3′; human *IFNB* reverse, 5′-TCCTCAGGGATGTCAAAG-3′; human *ISG15* forward, 5′-CGCAGATCACCCAGAAGATCG-3′; human *ISG15* reverse, 5′-TTCGTCGCATTTGTCCACCA-3′; human *GAPDH* forward, 5′-GGAGCGAGATCCCTCCAAAAT-3′; human *GAPDH* reverse, 5′-GGCTGTTGTCATACTTCTCATGG-3′; human *ZDHHC3* forward, 5′-GCTCTCATTTCCTTGCACGC-3′; human *ZDHHC3* reverse, 5′-AGTCTCTGCCATTTCTTCCCTG-3′; human *ZDHHC4* forward, 5′-TGGTCTTGCAAGGGATGGTTT-3′; human *ZDHHC4* reverse, 5′-GCAGCAGATAGGGCAGAAGAA-3′; human *ZDHHC5* forward, 5′-TGGCAGTAATGTGTGTGGCT-3′; human *ZDHHC5* reverse, 5′-GTTCATTGGTTGTGCGTCCC-3′; human *ZDHHC6* forward, 5′-AGCTGCATTTGCTACCACCT-3′; human *ZDHHC6* reverse, 5′-AGCCTTCTCTTCAATCCATGACT-3′; human *ZDHHC7* forward, 5′-TCATGACGTGGCTTCTGGTC-3′; human *ZDHHC7* reverse, 5′-CCCGTTGACCACAGAGTACC-3′; human *ZDHHC8* forward, 5′-ATGGACCCTGGTGTTTTCCC-3′; human *ZDHHC8* reverse, 5′-CATGCGGACCTGGATACCTC-3′; human *ZDHHC9* forward, 5′-CCTGCCATCCCTGTATTTGCT-3′; human *ZDHHC9* reverse, 5′-TAGCGCCCGAGGAATCACT-3′; human *ZDHHC12* forward, 5′-ACCCTGGCTACGTGAATGTG-3′; human *ZDHHC12* reverse, 5′-TGCAGCACCAGGCAGTATC-3′; human *ZDHHC13* forward, 5′-TCCTCTTCACTGGGCCATCC-3′; human *ZDHHC13* reverse, 5′-AATACTGCCAGGTGGATGCT-3′; human *ZDHHC16* forward, 5′-ACCCCAGGGCAGGAATGAT-3′; human *ZDHHC16* reverse, 5′-CCCACACAATTGTTTAGCCAG-3′; human *ZDHHC17* forward, 5′-TACGGCAACCGGACAAAGAA-3′; human *ZDHHC17* reverse, 5′-GGTCCCCTCCAAGTTGATCC-3′; human *ZDHHC18* forward, 5′-TGACTGTCCCTACCTGGCTC-3′; human *ZDHHC18* reverse, 5′-AGGCAGCTCATGACGAAGAA-3′; human *ZDHHC20* forward, 5′-TGGAGCTCTGCGTGTTTACT-3′; human *ZDHHC20* reverse, 5′-GAACAGATGGAAAGCCACAAGG-3′; mouse *Ifnb* forward, 5′-AGATCAACCTCACCTACAGG -3′; mouse *Ifnb* reverse, 5′-TCAGAAACACTGTCTGCTGG-3′; mouse *Isg15* forward, 5′-TCCATGACGGTGTCAGAACT-3′; mouse *Isg15* reverse, 5′-GACCCAGACTGGAAAGGGTA-3′; mouse *Zdhhc12* forward, 5′-CCCTGCTGCTTTATCTGGCT-3′; mouse *Zdhhc12* reverse, 5′-TGCGTTCACCCACACAGTTC-3′; mouse *Zdhhc4* forward, 5′-TGATTTGTGTTGTCCTGATCTGC-3′; mouse *Zdhhc4* reverse, 5′-GTGCCCCACTTGCGATTTAAG-3′; mouse *Gapdh* forward, 5′-GAAGGGCTCATGACCACAGT-3′; mouse *Gapdh* reverse, 5′-GGATGCAGGGATGATGTTCT-3′; *VSV-G* forward, 5′-CAAGTCAAAATGCCCAAGAGTCACA-3′; *VSV-G* reverse, 5′-TTTCCTTGCATTGTTCTACAGATGG-3′.

### Enzyme-linked immunosorbent assay.

The secretion levels of IFN-β or KL-6 in the cell supernatants or serum samples were determined by enzyme-linked immunosorbent assay (ELISA) using a human IFN-β kit (InvivoGen, catalog luex-hifnbv2), mouse IFN-β kit (InvivoGen, catalog luex-mifnbv2), or mouse KL-6 kit (JINGMEI, catalog JM-11515M1), respectively, according to the manufacturers’ protocols.

### RNA sequencing analysis.

Total RNA was extracted from BMDMs using TRIzol, and high-throughput sequencing was performed by Sangon Biotech Co. using the Illumina platform. Quality control of the FASTQ data was assessed with FastQC. HISAT2 was used to align the high-quality reads to the mouse reference genome (mm10) (64). The BAM files were sorted using SAMtools (https://www.htslib.org/), and the total number of reads that mapped to the genome was determined using the HTSeq-count program. The differential gene expression analysis was performed with the DESeq2 R package, and a *P* less than 0.05 and a fold change greater than 4 were used as the thresholds. Gene enrichment was analyzed using the clusterProfiler R package. Gene Ontology analysis, Kyoto Encyclopedia of Genes and Genomes (KEGG) pathway analysis, and gene set enrichment analysis (GSEA) were performed using the Database for Annotation, Visualization and Integrated Discovery (DAVID) tool, KEGG Automatic Annotation Server, and GSEA software, respectively.

### Immunoblot analysis.

HEK293T cells, THP-1–derived macrophages, and BMDMs were transfected or stimulated as indicated. The cells were subsequently harvested in low-salt lysis buffer (50 mM HEPES [pH 8.0], 150 mM NaCl, 1 mM EDTA, 1.5 mM MgCl_2_, 10% glycerol, and 1% Triton X-100) containing phosphatase inhibitor (Roche) and EDTA-free protease inhibitor (Biosharp), incubated on a rocker with ice for 30 minutes, and then centrifuged at 12,000*g* at 4°C for 15 minutes to obtain whole-cell lysates. The cell lysates were boiled with 5× SDS loading buffer at 95°C for 5 minutes and resolved by SDS-PAGE. For immunoprecipitation, the indicated whole-cell lysates were incubated with anti-FLAG beads (Sigma-Aldrich) or the appropriate antibodies plus protein A/G beads (Pierce) on a 3D shaker at 4°C overnight. The beads were washed 3 to 5 times with low-salt lysis buffer, and the immunoprecipitates were eluted with 2× SDS loading buffer for subsequent SDS-PAGE. For the MAVS ubiquitination assay, 1% SDS was added to the low-salt lysis buffer, and the cell lysates were denatured at 95°C for 5 minutes. Then, the denatured lysates were diluted with 0.1% SDS for immunoprecipitation with the indicated antibodies. The beads were washed several times by full immersion in low-salt lysis buffer, eluted with 2× SDS loading buffer, and resolved by SDS-PAGE. Proteins were transferred to Immobilon-PSQ Transfer Membranes (Millipore, catalog ISEQ00010) and incubated with the appropriate antibodies. Protein detection was performed using Immobilon-Western Chemiluminescent HRP Substrate (Millipore). Images were assayed using ChemiDoc MP System (Bio-Rad Laboratories Inc.) and Image Lab version 6.0 (Bio-Rad Laboratories Inc.).

### Semi-denaturing detergent agarose gel electrophoresis (SDD-AGE).

The cell lysates were harvested as described above in the *Immunoblot analysis* section. Then, the cell lysates were resuspended in 1× sample buffer (0.5× TBE buffer [Sigma-Aldrich, catalog T3913], 10% glycerol [Vetec, catalog V900122], 2% SDS [Ruishu Biotechnology, catalog 151-21-3], and 0.0025% bromophenol blue [Beyotime, catalog ST2258]) and loaded into a 1.5% agarose gel (containing 10% SDS). After electrophoresis in running buffer (1× TBE buffer containing 0.1% SDS) for 40 minutes at a constant voltage of 100 V at 4°C, the proteins were transferred to Immobilon-PSQ transfer membranes (Millipore, catalog ISEQ00010) for immunoblotting.

### Flow cytometry.

HEK293T cells were transfected with the indicated plasmids for 24 hours and then infected with VSV-eGFP for 12 hours. VSV-eGFP replication was examined by microscopy (DMi8, Leica). After trypsin digestion, the cells were washed with PBS twice and analyzed by flow cytometry using an LSR Fortessa Cell Analyzer (BD Biosciences) and FlowJo version 10.0 (Tree Star Software).

### Confocal analysis.

HEK293T cells (2 × 10^4^/mL) were cultured in glass-bottom culture dishes (Nest Scientific, catalog 801002) and transfected/treated as indicated. The culture medium was subsequently gently removed. The cells were washed twice with PBS and fixed with 4% paraformaldehyde for 15 minutes. After the cells were washed 3 times, precooled methyl alcohol at –20°C was added to the dishes for 20 minutes, followed by 3 washes with PBS. The cells were blocked in 6% goat serum (Boster Biological, catalog AR1009) at room temperature for 1 hour and incubated with primary antibodies diluted in 6% goat serum at 4°C overnight. After washing with PBS 3 more times, the cells were incubated with fluorescently labeled secondary antibodies (Alexa Fluor 488– or Alexa Fluor 568–conjugated antibodies against mouse or rabbit) for 1 hours. Nuclei were counterstained with DAPI (Sigma-Aldrich, catalog D9542) for 5 minutes. Confocal images were obtained using a microscope (TCS-SP8, Leica) equipped with 100×1.40 NA oil objectives and processed for gamma adjustments using Leica AS Lite. Colocalization was quantified (quantification of >12 cells) by calculation of Pearson’s correlation coefficient using ImageJ software (National Institutes of Health).

### Fluorescence resonance energy transfer analysis.

HEK293T cells were seeded in glass-bottom culture dishes and transfected/treated as indicated. Then, the cells were washed twice with PBS, fixed with 4% paraformaldehyde for 15 minutes, and mounted in PBS. A fluorescence resonance energy transfer (FRET) assay was performed with a Leica TCS SP8 confocal microscopy system using FRET SE (sensitized emission) Leica software, as described previously (18).

### Acyl-biotin exchange assay.

The indicated cells were collected in low-salt lysis buffer, incubated on a rocker with ice for 30 minutes, and then centrifuged at 12,000*g* at 4°C for 15 minutes. The cell lysates were incubated with TCEP (500 mM; Sangon Biotech, catalog 51805-45-9) at 55°C for 1 hour and then incubated with *N*-ethylmaleimide (NEM) (50 mM; Thermo Fisher Scientific, catalog 23030) at 4°C overnight. The NEM was then removed from the cell lysates by repeated methanol-chloroform precipitation. The protein pellets were dried at room temperature and dissolved in SDS buffer containing 4% SDS, 5 mM EDTA, 50 mM Tris (pH 8.0), and protease inhibitor at 37°C. Each sample was divided into 2 parts and mixed with the thiol-reactive biotin molecule HPDP-biotin (0.4 mM; Thermo Fisher Scientific, catalog 21341). The part for which the HAM (Sigma-Aldrich, catalog 379921) cleavage step was omitted (– HAM) was supplemented with lysis buffer, and the part for which the HAM step was included (+ HAM) was supplemented with 1 M HAM. After 5 hours of incubation at 4°C, the samples were pelleted by methanol-chloroform precipitation and resuspended in 4% SDS buffer. After dissolution, one-fifth of the samples were eluted with 5× SDS loading buffer and boiled at 95°C for 5 minutes for “input,” and the remaining samples were incubated with neutravidin agarose beads (Thermo Fisher Scientific, catalog 29200) at 4°C overnight. The beads were washed 4 times with lysis buffer, eluted with 2× SDS loading buffer, and subsequently resolved by SDS-PAGE. Quantification of the relative palmitoylation (Rel. palm) level of MAVS (normalized to “input” MAVS) was determined by ImageJ software.

### Luciferase reporter assays.

HEK293T cells were plated in 24-well plates and transfected with plasmids encoding the IFN-β/ISRE luciferase reporter (firefly luciferase) and pRL-TK (Renilla luciferase), together with the indicated plasmids, using Lipofectamine 2000. After 24 hours, the cells were infected or not infected with SeV (MOI = 1) for 12 hours. The cells were subsequently collected and lysed in Passive Lysis Buffer (Promega, catalog E1941). The luciferase activity was measured with a Dual-Luciferase Assay (Promega, catalog E1910) with a Luminoskan Ascent luminometer (Thermo Fisher Scientific, catalog 2805621) according to the manufacturer’s protocol. Induction firefly luciferase activity was normalized to Renilla luciferase activity.

### Statistics.

All the quantitative data were analyzed using GraphPad Prism (GraphPad software Inc.). Data are presented as the mean value ± SEM or ± SD, as indicated in the figure legends. Data were analyzed by 2-tailed Student’s *t* test for 2-group comparisons or by 1-way ANOVA for multiple comparisons. The log-rank (Mantel-Cox) test was performed for survival studies. *P* less than 0.05 was considered statistically significant.

### Study approval.

All animal experimental protocols were approved by the Animal Care Committee of Sun Yat-sen University [authorization number: SYXK (YUE) 2023-0313; Guangzhou, China]. The mice were euthanized with CO_2_ from compressed gas cylinders in compliance with all ethical regulations.

### Data availability.

The RNA sequencing data were submitted to the Sequence Read Archive database under accession number PRJNA1144358. Values for all data points in graphs are shown in the Supporting Data Values file. See complete unedited blots in the supplemental material.

## Author contributions

LW and ML performed the investigation and the analysis. GL, SY, J Cai, ZC, and YW provided technical help. J Cui provided resources, conceived the idea, and directed the research. LW and J Cui wrote the manuscript.

## Supplementary Material

Supplemental data

Unedited blot and gel images

Supporting data values

## Version 1 12/02/2024

Electronic publication
