# Expression of Concern for Palmitoylation acts as a checkpoint for MAVS aggregation to promote antiviral innate immune responses

**DOI:** 10.1172/JCI213314

**Published:** 2026-10-15

**Authors:** Liqiu Wang, Mengqiu Li, Guangyu Lian, Shuai Yang, Jing Cai, Zhe Cai, Yaoxing Wu, Jun Cui

Original citation: J Clin Invest. 2024;134(23):e177924. https://doi.org/10.1172/JCI177924

Citation for this expression of concern: J Clin Invest. 2026;136(20):e213314. https://doi.org/10.1172/JCI213314

The authors recently alerted the Editors to errors in the source data for Figures 4E, 5F, and 6M and Supplemental Figure 3H that were identified in a post-publication audit of the data. Although the authors have indicated that the errors were due to inadvertent mistakes, the Editors are issuing this Expression of Concern to alert readers to the issues. We have requested institutional oversight into this matter and will alert readers to the outcome when the institutional evaluation is complete.

## Version 1 10/15/2026

Electronic publication

