# Supplementary material for: Palmitoylation acts as a checkpoint for MAVS aggregation to promote antiviral innate immune responses
Source: J Clin Invest. 2024 Dec 2;134(23):e177924. doi: 10.1172/JCI177924 (PMC11601910; doi:10.1172/JCI177924)
Supplement: Supplemental data [file jci-134-177924-s008.pdf]

Supplementary Information

Palmitoylation acts as a checkpoint for MAVS aggregation to promote antiviral innate immune responses

Liqiu Wang[1], Mengqiu Li[1], Guangyu Lian[1], Shuai Yang[1], Jing Cai[1], Zhe Cai[2], Yaoxing Wu[3], Jun Cui[1]

1 MOE Key Laboratory of Gene Function and Regulation, Guangdong Province Key Laboratory of Pharmaceutical Functional Genes, State Key Laboratory of Biocontrol, Innovation Center of the Sixth Affiliated Hospital, School of Life Sciences of Sun Yat-sen university, Guangzhou, Guangdong, China

2 Guangzhou Institute of Pediatrics, Guangzhou Women and Children's Medical Center, Guangzhou, Guangdong, China

3 Department of Critical Care Medicine, The First Affiliated Hospital of Sun Yat-sen University, Guangzhou, Guangdong, China

Correspondence: cuij5@mail.sysu.edu.cn

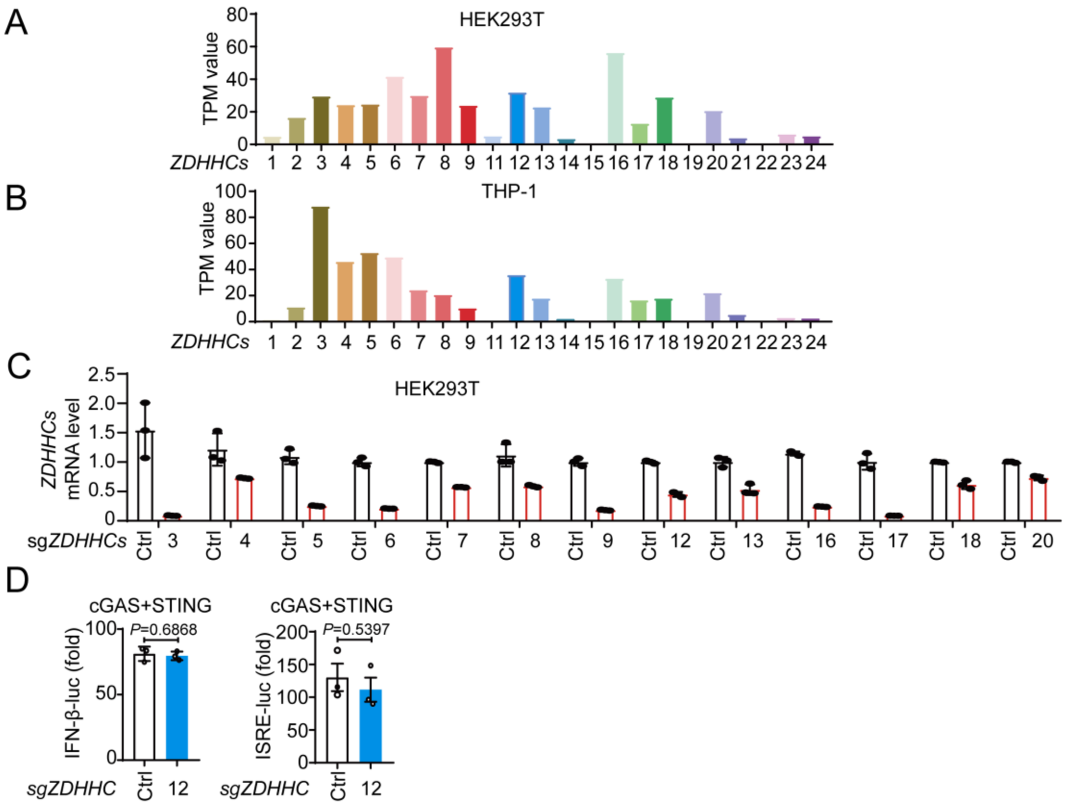

Supplementary Figure 1. Analysis of the expression of *ZDHHCs*.

(**A** and **B**) Expression level of *ZDHHC*s in human embryonic kidney (HEK) 293T cells (**A**) and THP-1 cells (**B**) according to The Human Protein Atlas (https://www.proteinatlas.org/). TPM: transcripts per million. (**C**) HEK293T cells were transfected with control single-guide RNA (sg*Ctrl*) or indicated *ZDHHCs*-specific sgRNA (sg*ZDHHCs*) for 48 h. Cell lysates were collected for real-time quantitative polymerase chain reaction (qPCR) analysis. (**D**) HEK293T cells were transfected with control single-guide RNA (sg*Ctrl*) or sg*ZDHHC12* for 48 h. The cells were transfected with plasmids encoding the IFN-stimulated response element (ISRE)-luc or IFNβ-luc reporter and pRL-TK, together with cGAS plus STING. Cell lysates were harvested for luciferase reporter assays. Data are presented as mean values ± SEM, statistical analysis was performed using two-tailed Student's t-test.

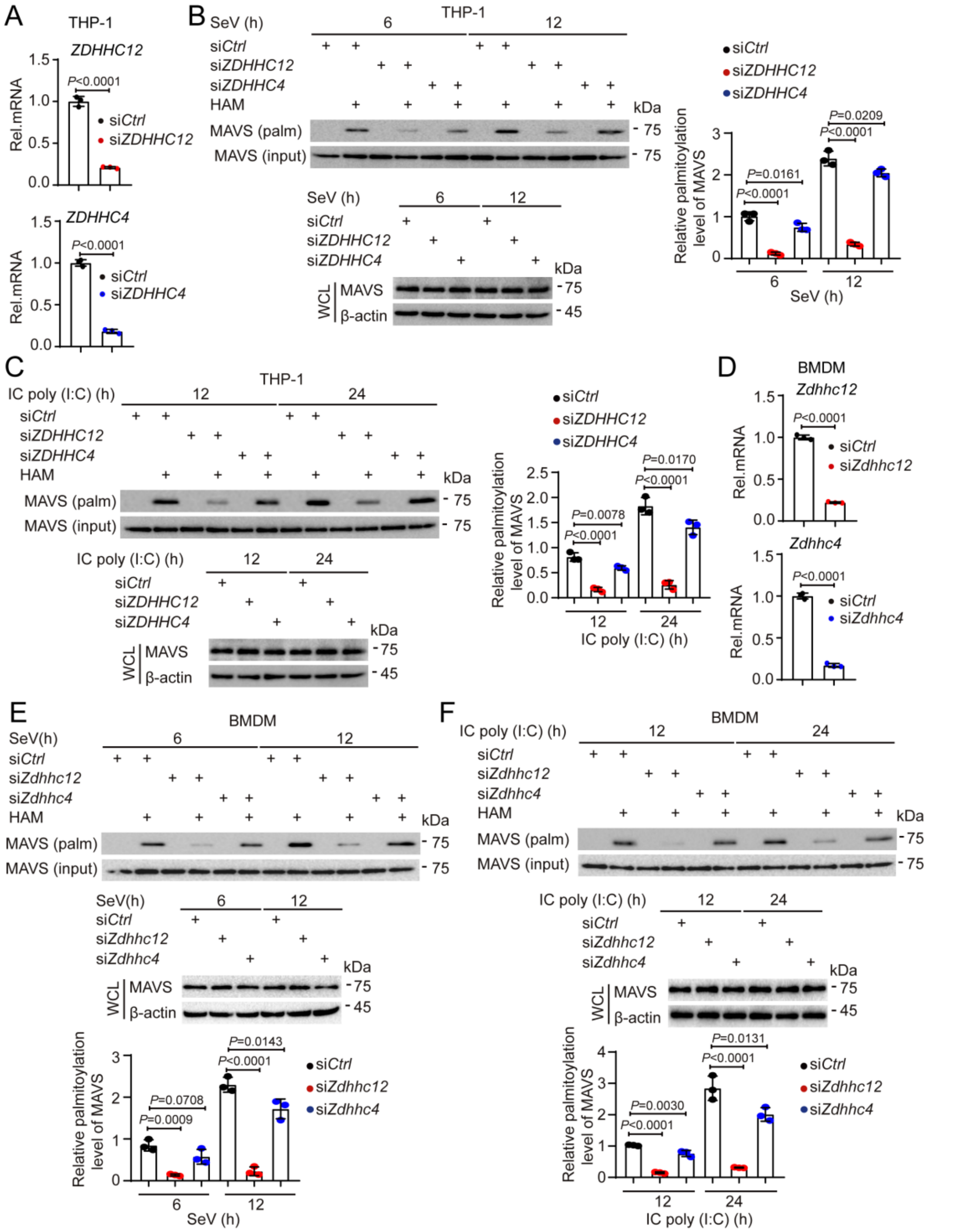

Supplementary Figure 2. ZDHHC12, but not ZDHHC4, plays a major role in palmitoylation of MAVS in macrophages.

(**A**) THP-1-derived macrophages were transfected with control small interfering RNA (si*Ctrl*), *ZDHHC12*-specific siRNA (si*ZDHHC12*), or *ZDHHC4*-specific siRNA (si*ZDHHC4*) for 48 h. Cell lysates were collected for real-time qPCR analysis for detecting knockdown efficiency. (**B** and **C**) THP-1-derived macrophages were

transfected with si*Ctrl*, si*ZDHHC12*, or si*ZDHHC4* for 48 h, and then infected with SeV (MOI=1) (**B**) or IC poly (I:C) (5 μg/mL) (**C**) for indicated time periods. Cell lysates were collected for immunoblot analysis and ABE assay. (**D**) Bone-marrow derived macrophages (BMDMs) were transfected with si*Ctrl*, si*Zdhhc12*, or si*Zdhhc4* for 48 h. Cell lysates were collected for real-time qPCR analysis for detecting knockdown efficiency. (**E** and **F**) BMDMs were transfected with si*Ctrl*, si*Zdhhc12*, or si*Zdhhc4* for 48 h, and then infected with SeV (MOI=1) (**E**) or IC poly (I:C) (5 μg/mL) (**F**) for indicated time periods. Cell lysates were collected for immunoblot analysis and ABE assay. In (**A** and **D**), data are presented as mean values ± SEM; in (**B**, **C**, **E** and **F**), quantification of relative palmitoylation level of MAVS (normalized to "input" MAVS) was determined by ImageJ software; data are presented as mean values ± SD. Statistical analysis was performed using two-tailed Student's t-test in **A** and **D** or one-way ANOVA multiple comparisons in **B**, **C**, **E** and **F**.

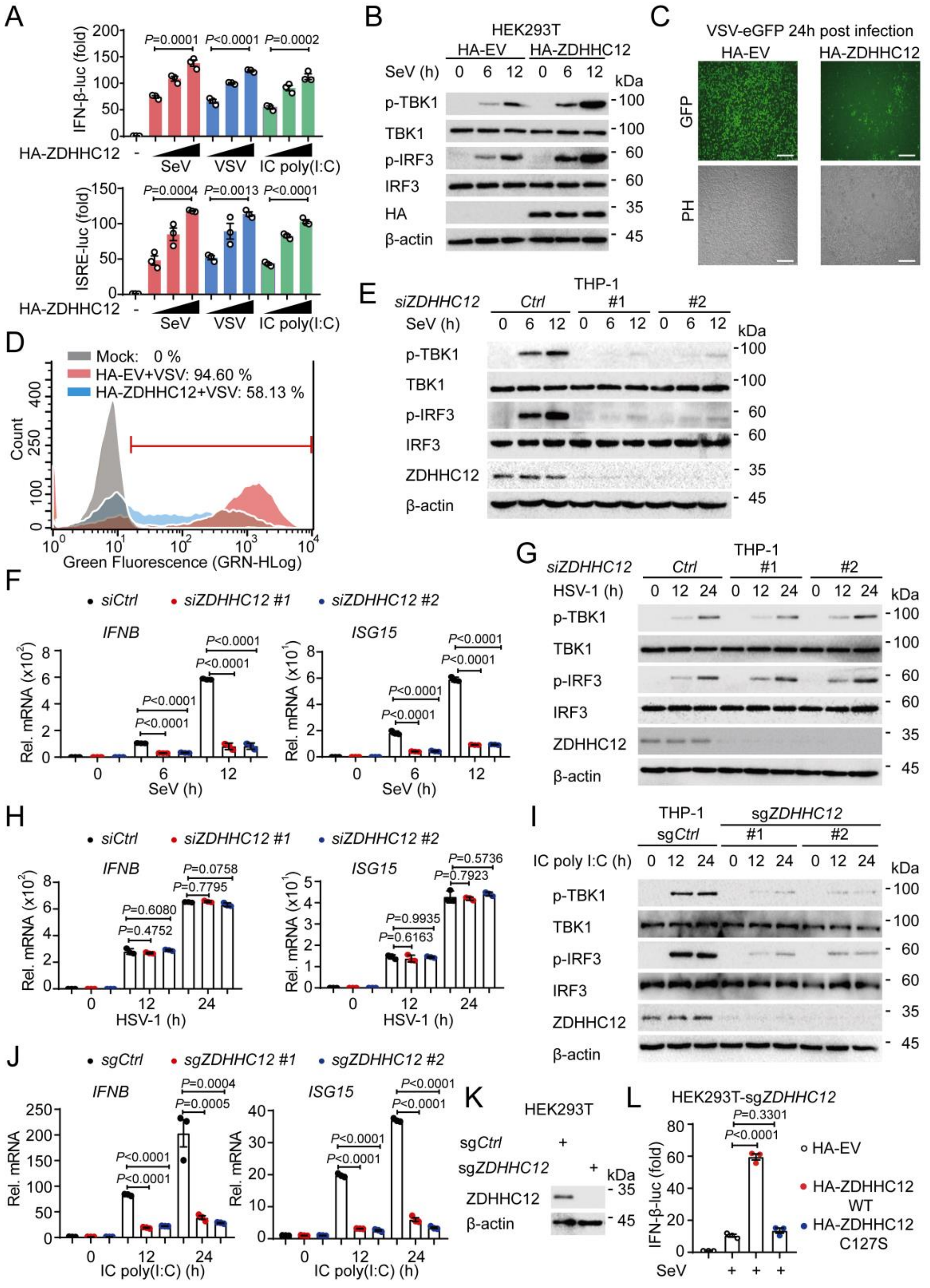

Supplementary Figure 3. ZDHHC12 positively regulates the activation of RLR-mediated type I IFN signaling.

(**A**) HEK293T cells were transfected with plasmids encoding the IFNβ/ISRE luciferase reporter and pRL-TK, together with empty vector or increasing amount of HA-ZDHHC12 vector for 24 h, and then treated with SeV (MOI=1), VSV-eGFP (MOI=1) or intracellular (IC) poly (I:C) (polyinosinic: polycytidylic acid, 5 μg/mL) for 24 h, respectively. Cell lysates were collected for luciferase reporter assays. (**B**) HEK293T cells were transfected with empty vector (EV) or HA-ZDHHC12 plasmid for 24 h, and then infected with SeV (MOI=1) as indicated time periods. Cell lysates were collected for immunoblot analysis. (**C** and **D**) Phase-contrast (PH) and fluorescence microscopy (**C**) and flow cytometric analysis (**D**) of HEK293T cells transfected with HA-EV or HA-ZDHHC12 plasmid, and then infected with VSV-eGFP (MOI=1). Scale bars, 50 μm. (**E-H**) THP-1-derived macrophages were transfected with control small interfering RNA (si*Ctrl*) or two *ZDHHC12*-specific siRNAs (si*ZDHHC12* #1, #2) for 48 h. And then infected with SeV (MOI=1, **E** and **F**) or HSV-1(MO1=1, **G** and **H**) for indicated time periods. Cell lysates were collected for immunoblot (**E** and **G**) and real-time qPCR analysis (**F** and **H**). (**I** and **J**) Wild type (WT, sg*Ctrl*) or *ZDHHC12*-knockout (KO, sg*ZDHHC12*) THP-1 macrophages were treated with IC poly (I:C) for indicated time periods. Cell lysates were collected for immunoblot (**I**) and real-time qPCR analysis (**J**). (**K**) The knockout efficiency of sg*ZDHHC12* in HEK293T cells were confirmed by immunoblot analysis. **(N)** *ZDHHC12*-KO (sg*ZDHHC12*) HEK293T cells were transfected with plasmids encoding the IFNβ luciferase reporter and pRL-TK, together with HA-EV, WT HA-ZDHHC12 or HA-ZDHHC12 C127S mutant. Cell lysates were collected for luciferase reporter assays. In (**A**, **F**, **H**, **J** and **L)**, data are presented as mean values ± SEM, statistical analysis was performed using one-way ANOVA multiple comparisons. In **(B-E**, **G**, **I** and **K**), similar results were obtained for three independent experiments.

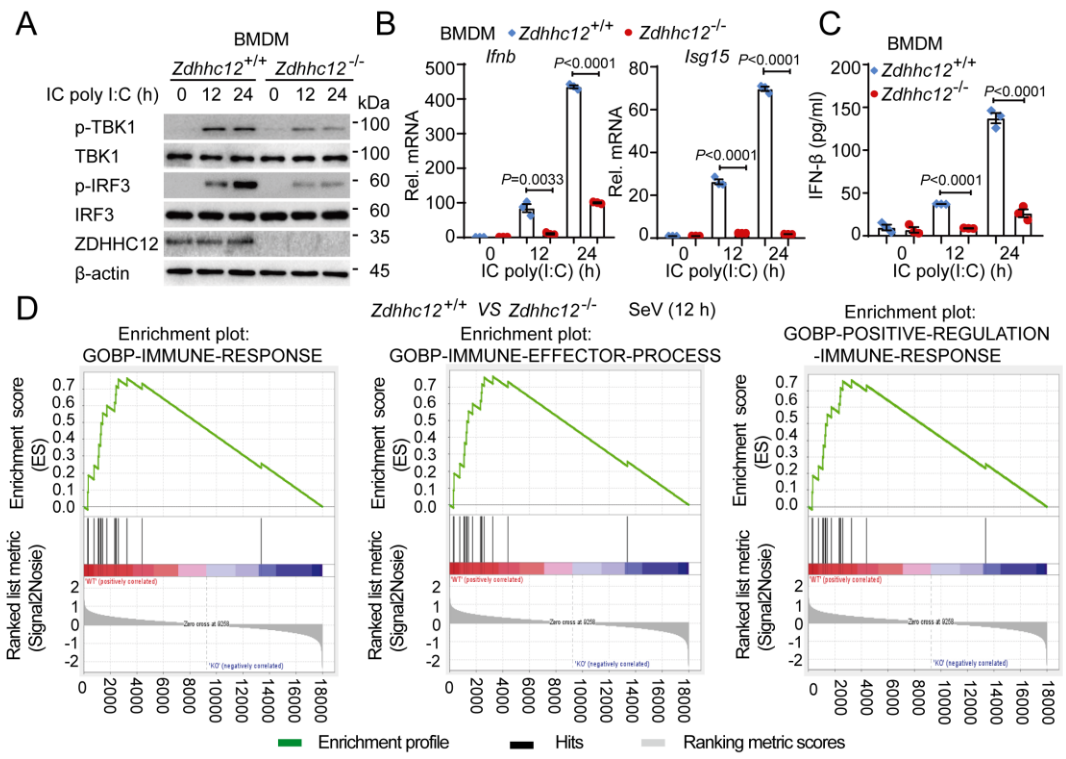

Supplementary Figure 4. *Zdhhc12* deficiency impairs antiviral innate immune responses.

(**A-C**) BMDMs isolated from *Zdhhc12*$^{+/+}$ or *Zdhhc12*$^{-/-}$ mice were treated with intracellular (IC) poly (I:C) (5 μg/mL) for indicated time periods. Cell lysates were collected for immunoblot (**A**) and real-time qPCR analysis (**B**). IFN-β release in the supernatants was determined by ELISA (**C**). (**D**) Gene-set-enrichment analysis (GSEA) of differentially expressed genes in *Zdhhc12*$^{+/+}$ or *Zdhhc12*$^{-/-}$ BMDMs with SeV (MOI=1) infection for 12 h. In (**A**), similar results were obtained for three independent experiments. In (**B** and **C**), data are presented as mean values ± SEM, statistical analysis was performed using two-tailed Student's t-test.

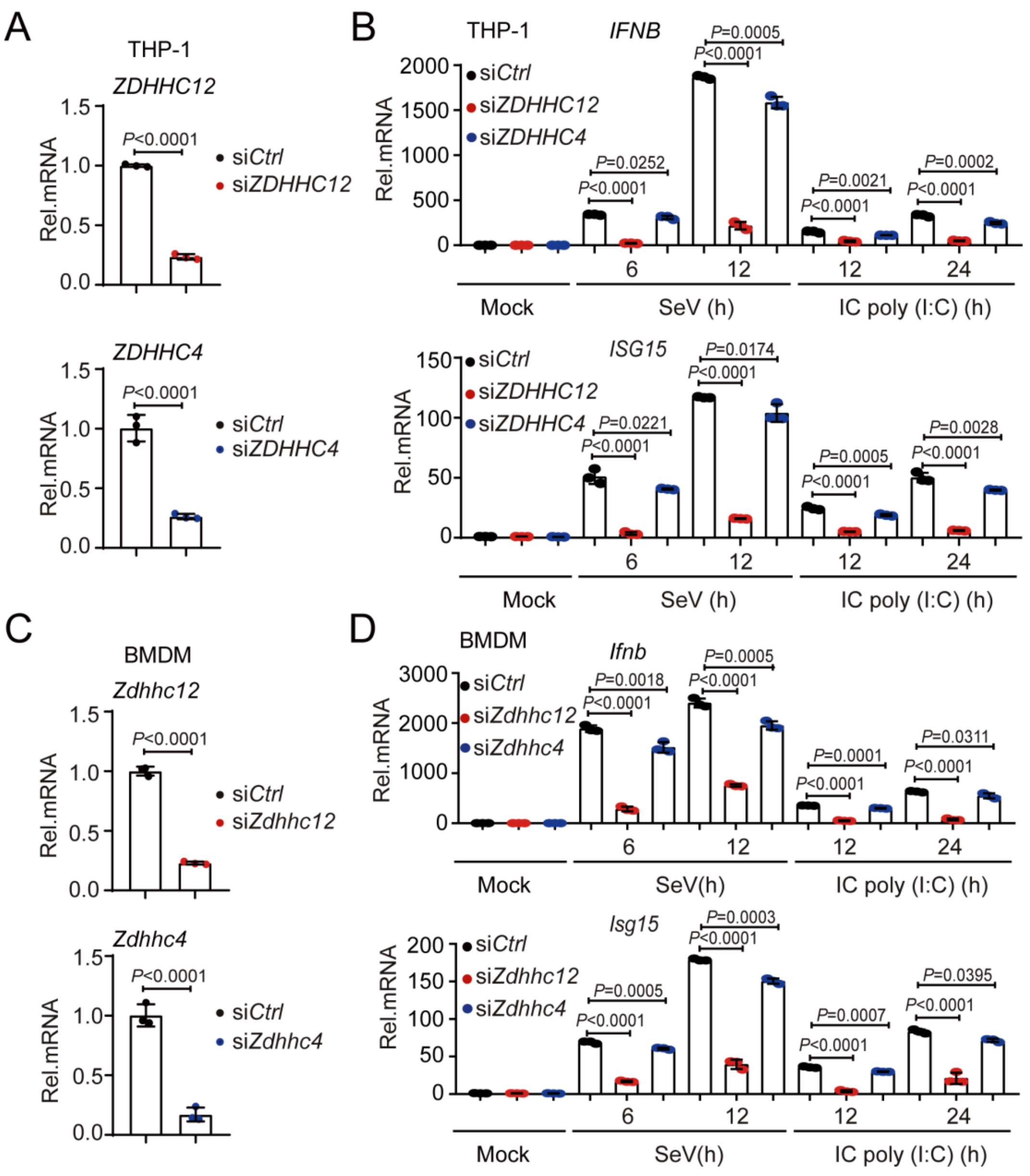

Supplementary Figure 5. ZDHHC12, but not ZDHHC4, plays a major role in the activation of RNA virus-mediated type I IFN signaling pathway in macrophages. (**A**) THP-1-derived macrophages were transfected with control small interfering RNA (si*Ctrl*), *ZDHHC12*-specific siRNA (si*ZDHHC12*), or *ZDHHC4*-specific siRNA (si*ZDHHC4*) for 48 h. Cell lysates were collected for real-time qPCR analysis for detecting knockdown efficiency. (**B**) THP-1-derived macrophages were transfected with si*Ctrl*, si*ZDHHC12*, or si*ZDHHC4* for 48 h, and then infected with SeV (MOI=1) or IC poly (I:C) (5 μg/mL) for indicated time periods. Cell lysates were collected for real-time qPCR analysis. (**C**) Bone-marrow derived macrophages

(BMDMs) were transfected with si*Ctrl*, si*Zdhhc12*, or si*Zdhhc4* for 48 h. Cell lysates were collected for real-time qPCR analysis for detecting knockdown efficiency. (**D**) BMDMs were transfected with si*Ctrl*, si*Zdhhc12*, or si*Zdhhc4* for 48 h, and then infected with SeV (MOI=1) or IC poly (I:C) (5 μg/mL) for indicated time periods. Cell lysates were collected for real-time qPCR analysis. In (**A-D**), data are presented as mean values ± SEM, statistical analysis was performed using two-tailed Student's t-test in **A** and **C** or one-way ANOVA multiple comparisons in **B** and **D**.

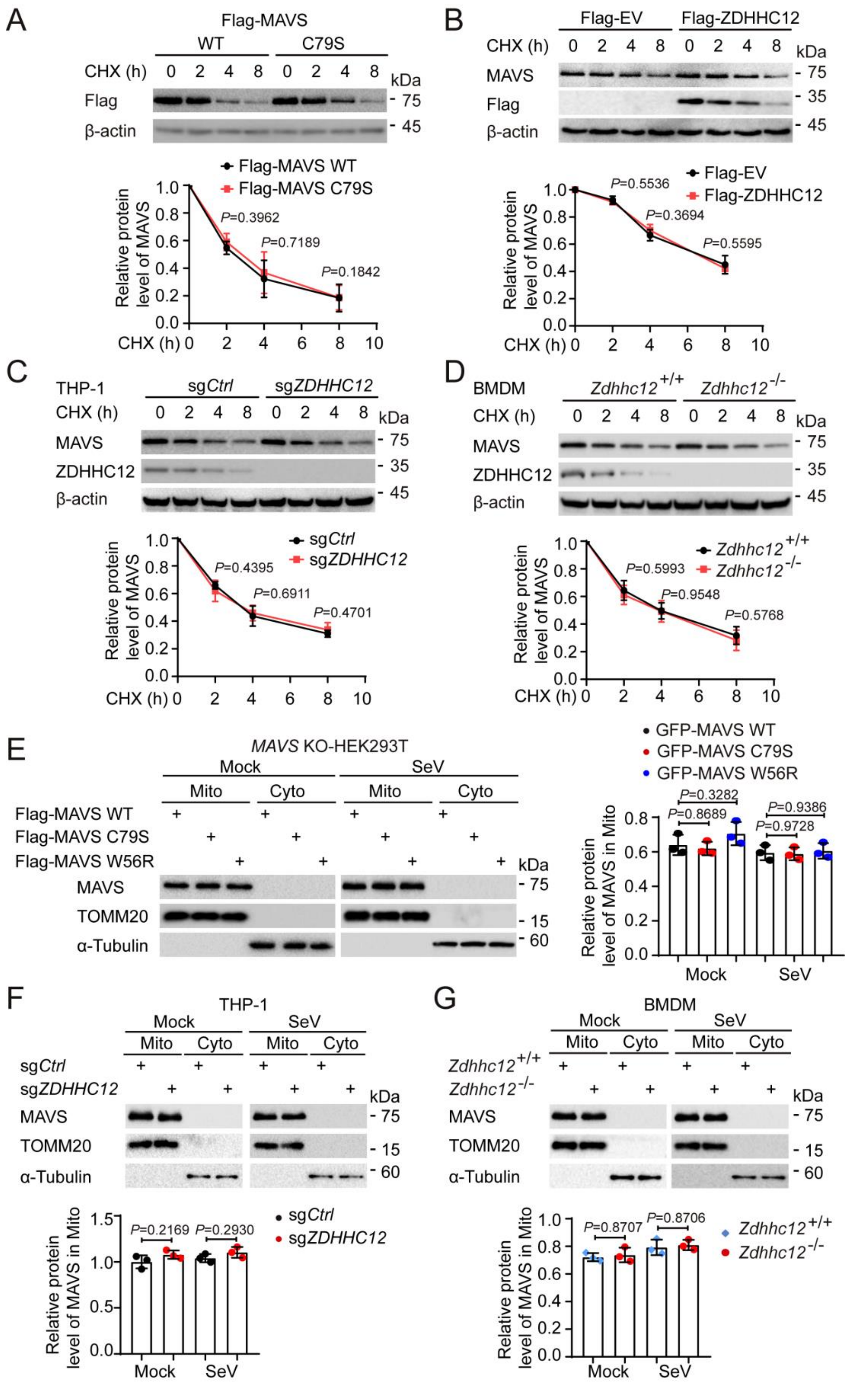

Supplementary Figure 6. Palmitoylation has no effect on the stability or

mitochondrial localization of MAVS.

(**A**) HEK293T cells were transfected with Flag-MAVS WT or Flag-MAVS C79S mutant for 24 h, then treated with cycloheximide (CHX, 10 μg/ml) as indicated time points. Cell lysates were collected for immunoblot analysis. (**B**) HEK293T cells were transfected with Flag-empty vector (EV) or Flag-MAVS C79S mutant for 24 h, then treated with CHX (10 μg/ml) as indicated time points. Cell lysates were collected for immunoblot analysis. (**C**) Wild type (WT, sg*Ctrl*) and *ZDHHC12*-knockout (KO, sg*ZDHHC12*) THP-1-derived macrophages were treated with CHX (10 μg/ml) treatment at the indicated time points. Cell lysates were collected for immunoblot analysis. (**D**) *Zdhhc12*$^{+/+}$ or *Zdhhc12*$^{-/-}$ BMDMs were treated with CHX (10 μg/ml) treatment at the indicated time points. Cell lysates were collected for immunoblot analysis. (**E-G**) *MAVS*-KO HEK293T cells were transfected with Flag-MAVS WT, Flag-MAVS C79S or Flag-MAVS W56R for 24 h, and then infected with SeV (MOI=1) for 12 h (**E**). Wild type (WT, sg*Ctrl*) and *ZDHHC12*-knockout (KO, sg*ZDHHC12*) THP-1-derived macrophages were infected with SeV (MOI=1) for 12 h (**F**). *Zdhhc12*$^{+/+}$ or *Zdhhc12*$^{-/-}$ BMDMs were infected with SeV (MOI=1) for 12 h (**G**). The mitochondrial and cytoplasmic fractions of indicated cells were isolated by a mitochondria isolation kit (Beyotime Biotechnology), and then immunoblot analysis was performed to detect MAVS, TOMM20 (mitochondria marker) and α-tubulin (cytosol marker). In (**A-G**), similar results were obtained for three independent experiments, quantification of MAVS protein levels was determined by ImageJ software, data are presented as mean values ± SD, statistical analysis was performed using two-tailed Student's t-test in **A-D**, **F** and **G** or one-way ANOVA multiple comparisons in **E**.

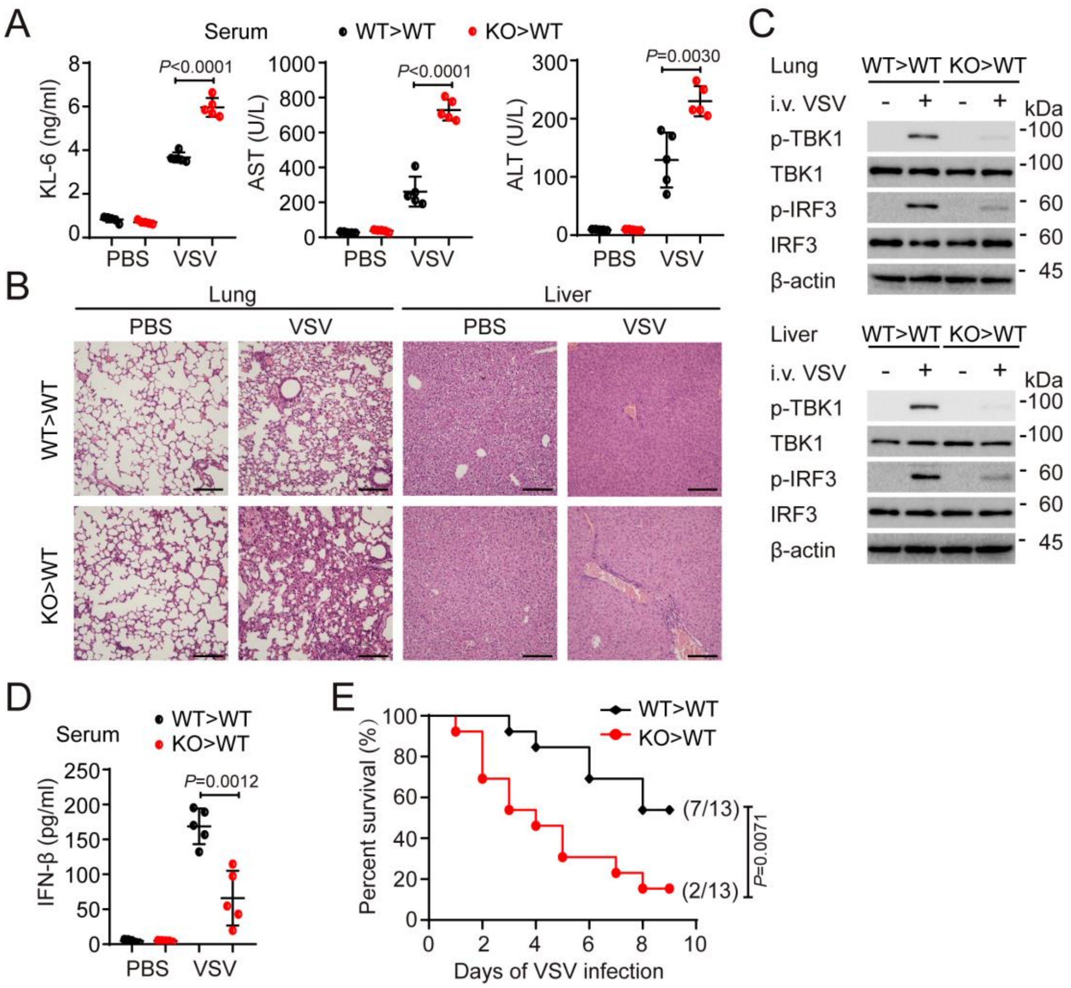

Supplementary Figure 7. *ZDHHC12* deficiency in bone marrow-derived cells impairs antiviral immunity in vivo.

(**A-D**) The WT>WT mice (wild-type mice as both recipient and donor mice, n=5 per group) and KO>WT mice (wild-type mice as recipient mice and *Zdhhc12*-KO mice as donor mice, n=5 per group) were intravenously injected with VSV (1×10^8 pfu/mouse) for 24 h. The production of KL-6, ALT and AST in serum of indicated mice was determined by ELISA (**A**). The lung and liver tissues were isolated and stained with hematoxylin and eosin (H&E), and assayed using a light microscope. Scale bars, 100 µm (**B**). The phosphorylation levels of TBK1 and IRF3 of the lung or liver tissues (the mixture of 5 mice per group) were detected by immunoblot analysis (**C**). The production of IFN-β in serum of indicated mice was determined by ELISA (**D**). Each symbol represents an individual mouse; data are presented as mean values ± SD, statistical analysis was performed using two-tailed Student's t-test. (**E**) Survival curve

of WT>WT mice or KO>WT mice (n=13 per group) given intravenously injected with VSV (1×10^8 pfu/mouse). The statistical test of difference was calculated using log-rank (Mantel-Cox) test.
