# Supplementary material for: Palmitoylation acts as a checkpoint for MAVS aggregation to promote antiviral innate immune responses
Source: J Clin Invest. 2024 Dec 2;134(23):e177924. doi: 10.1172/JCI177924 (PMC11601910; doi:10.1172/JCI177924)

Full unedited blot for Figure 1A

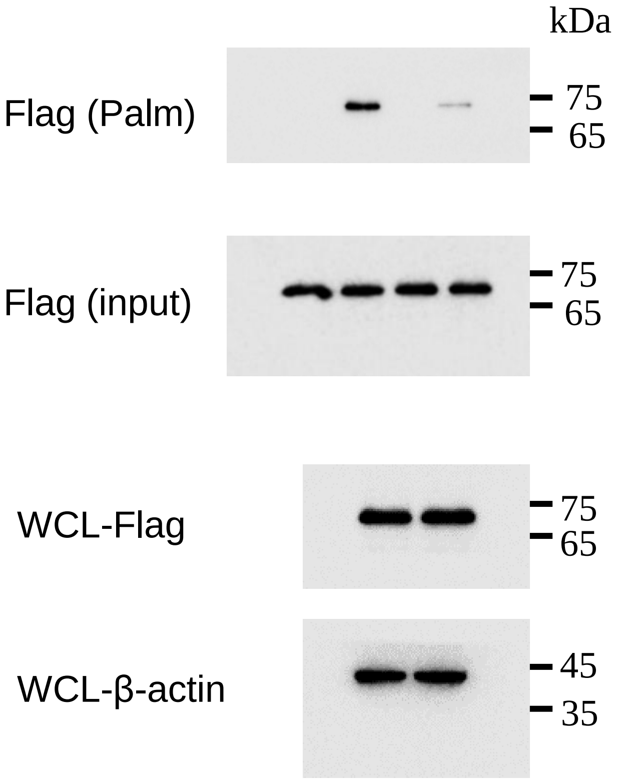

Full unedited blot for Figure 1C

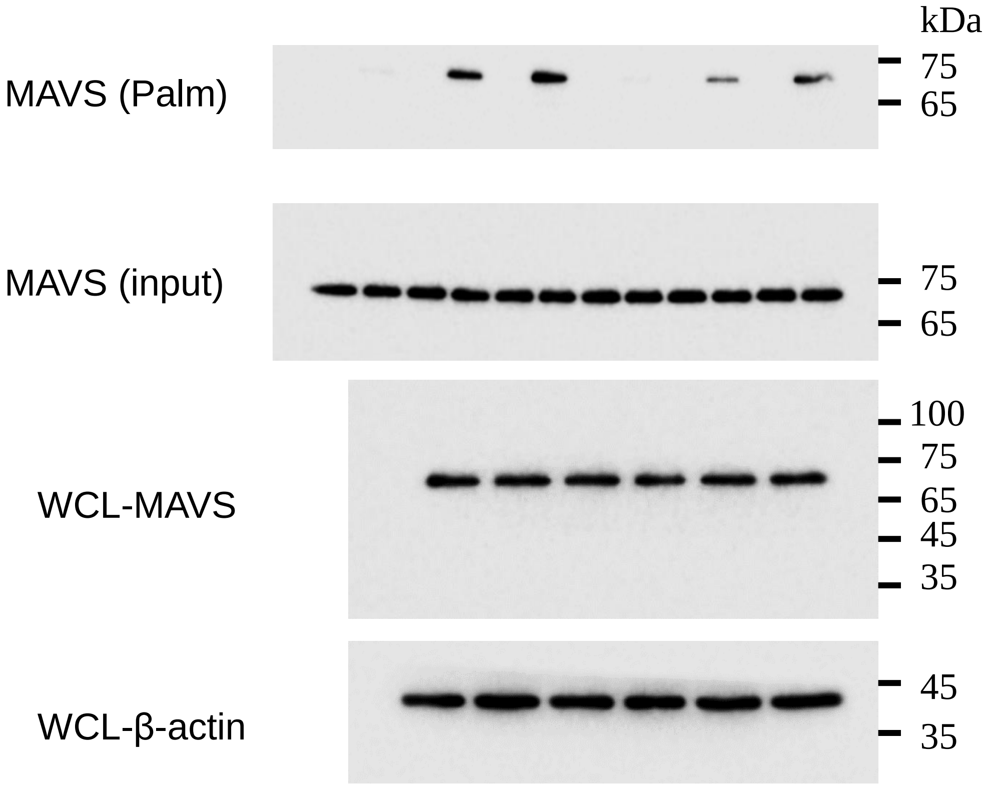

Full unedited blot for Figure 1D

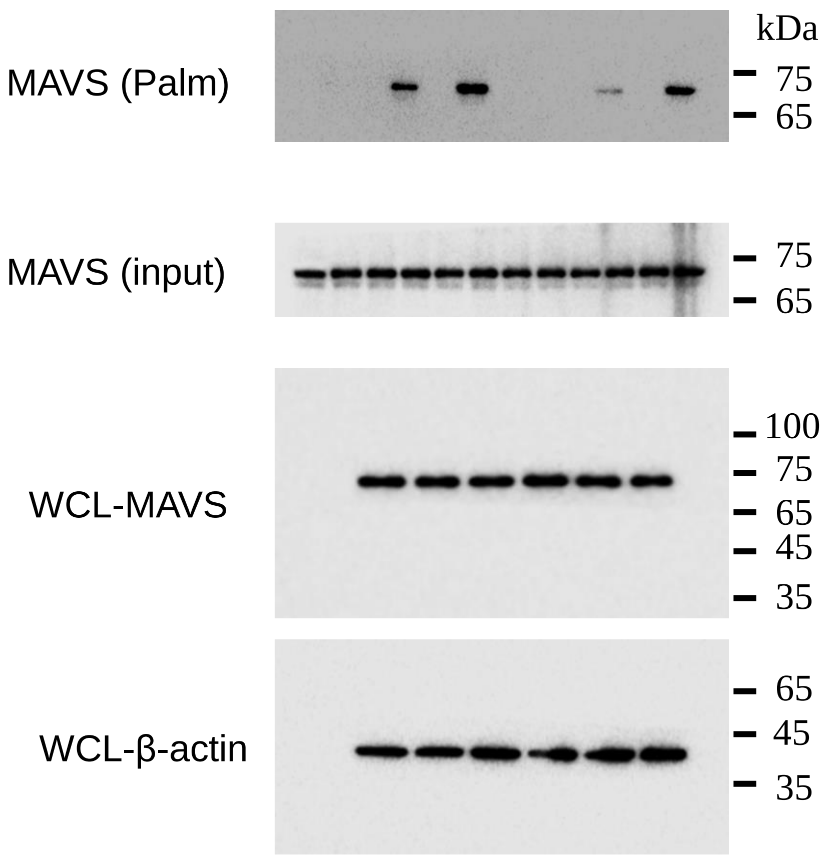

Full unedited blot for Figure 1E

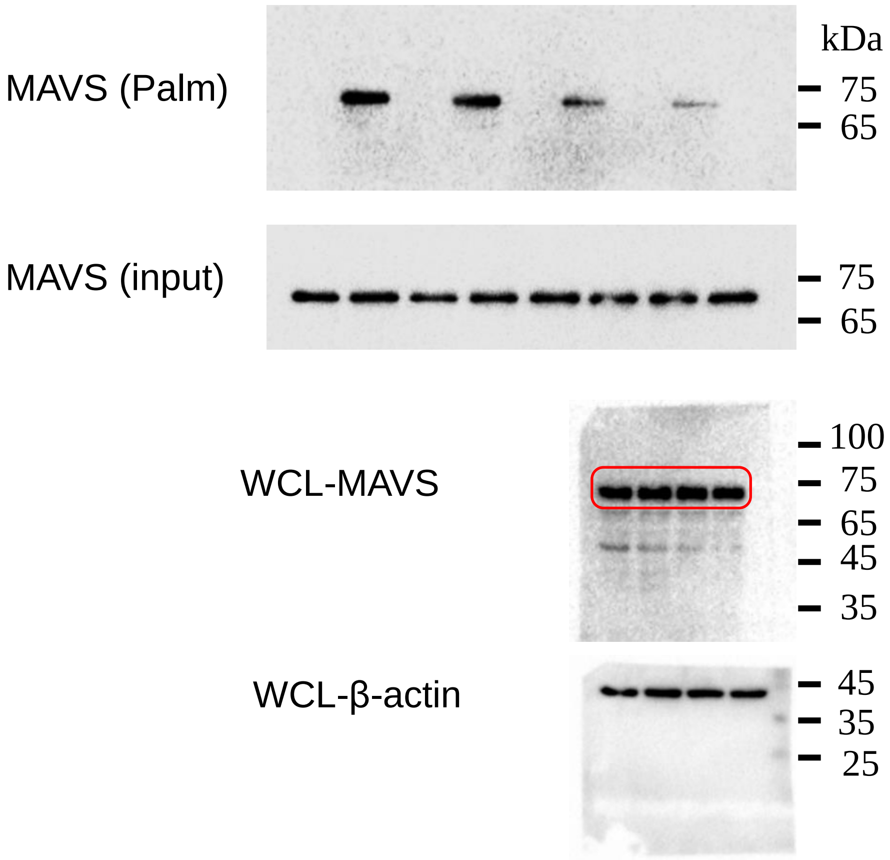

Full unedited blot for Figure 1G

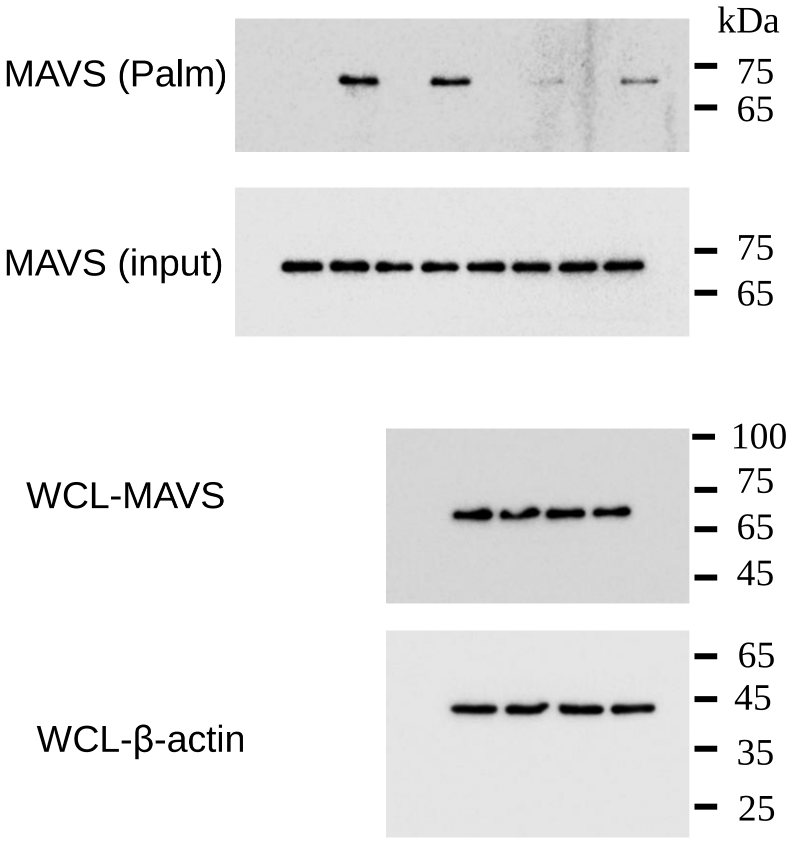

Full unedited blot for Figure 1I

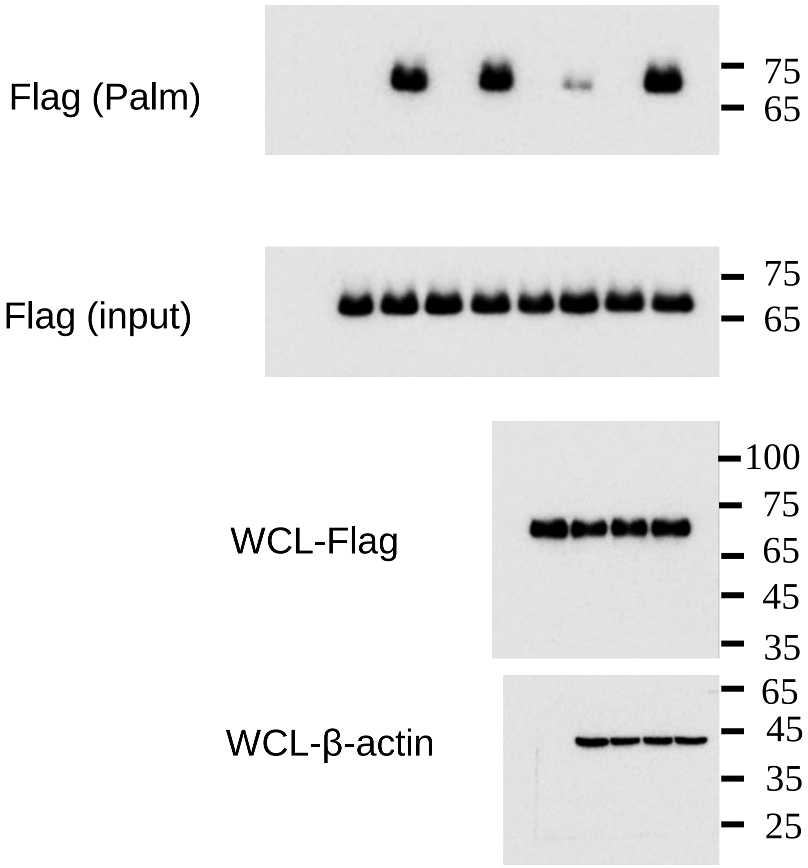

Full unedited blot for Figure 1L

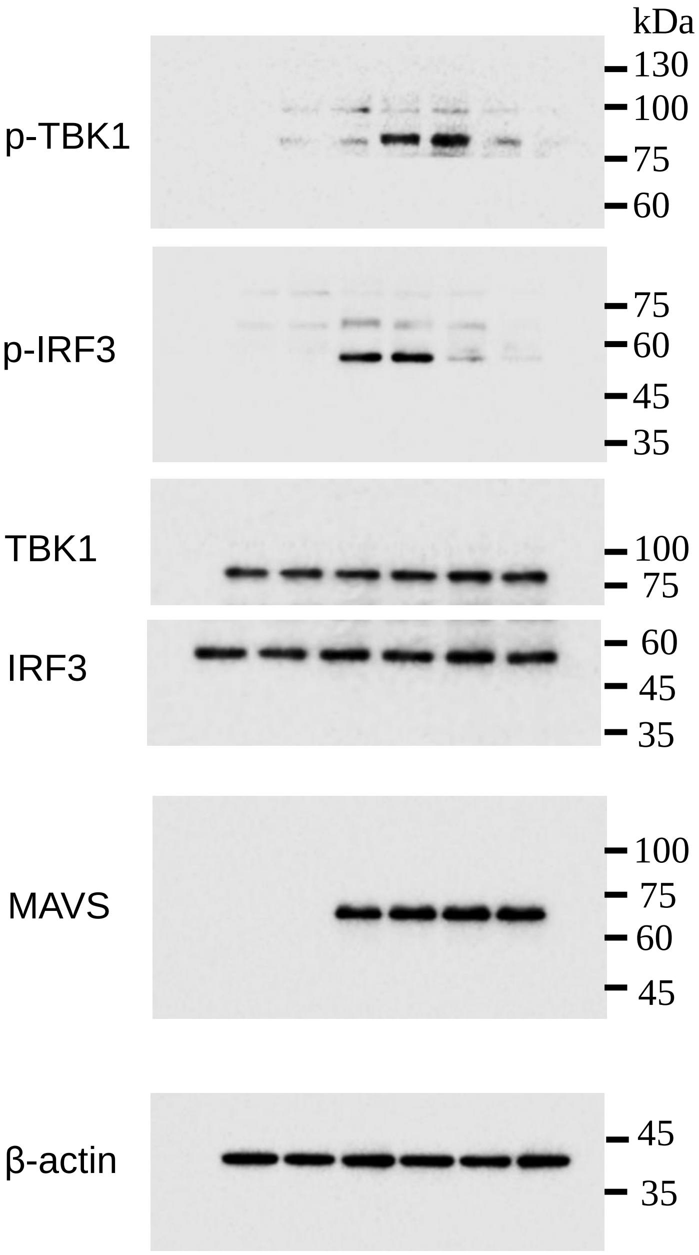

Full unedited blot for Figure 1O

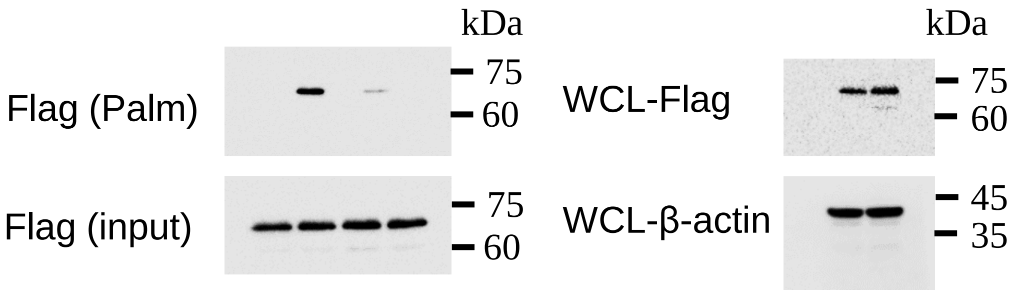

Full unedited blot for Figure 1Q

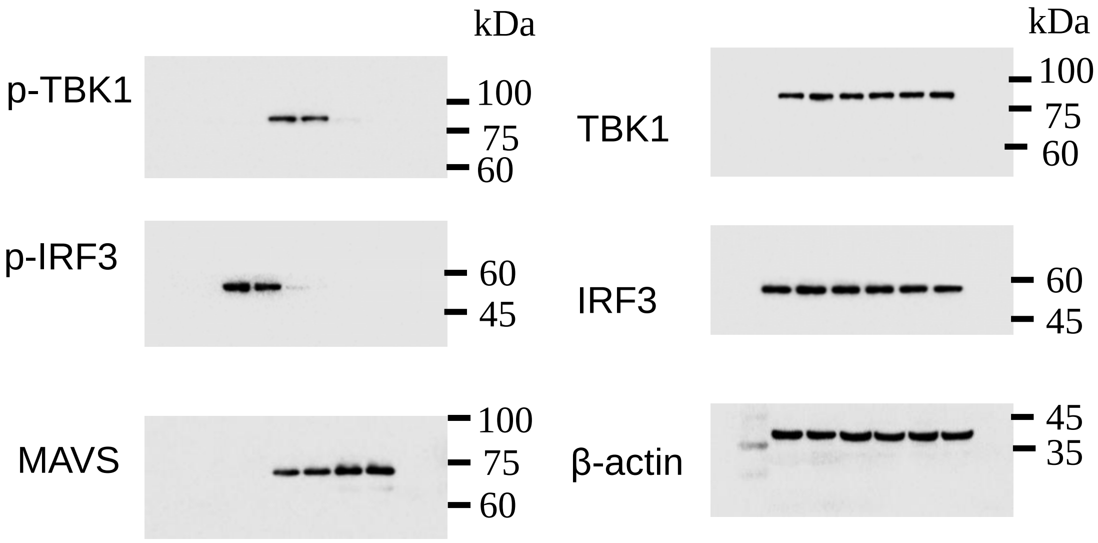

Full unedited blot for Figure 2B

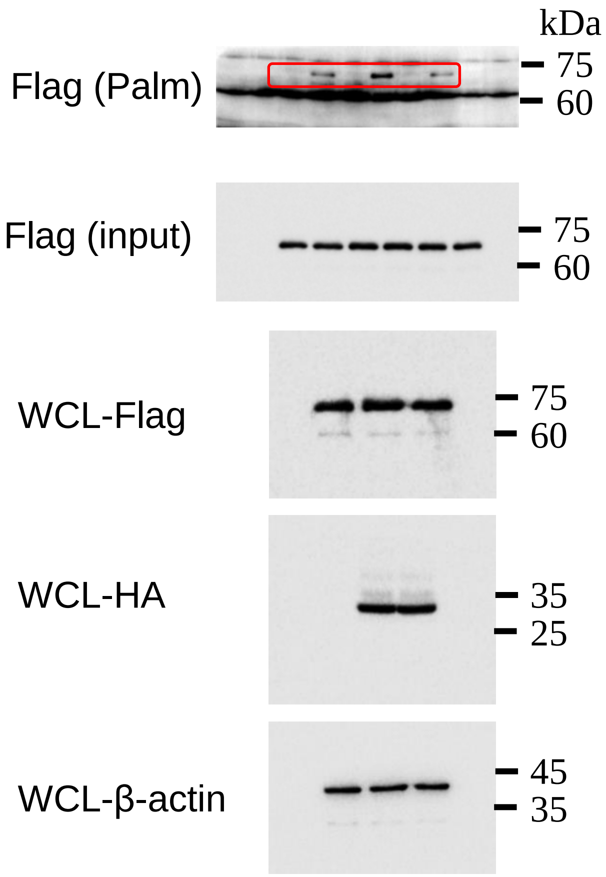

Full unedited blot for Figure 2D

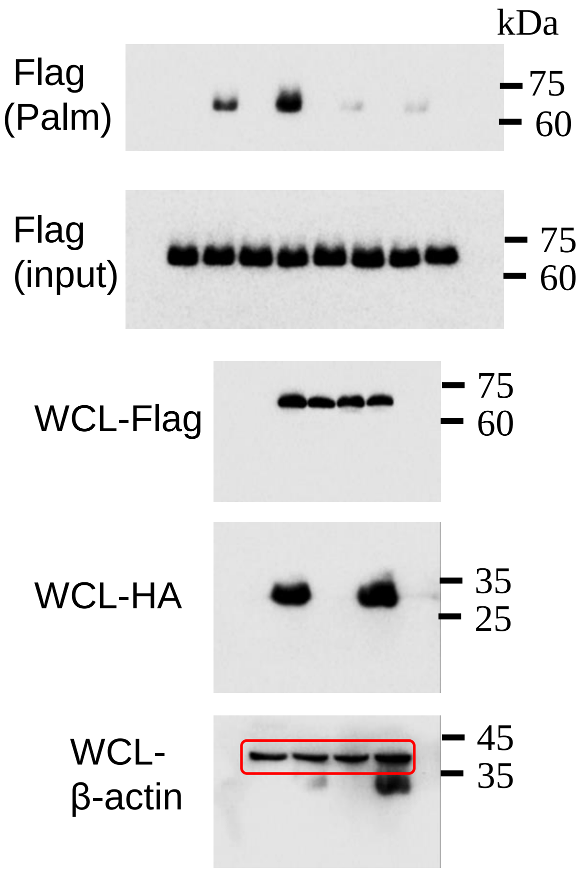

Full unedited blot for Figure 2F

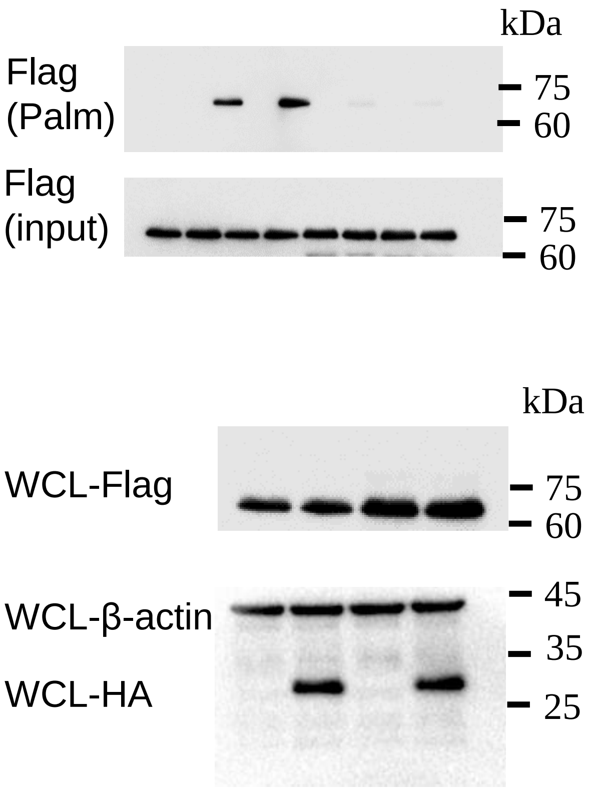

Full unedited blot for Figure 2H

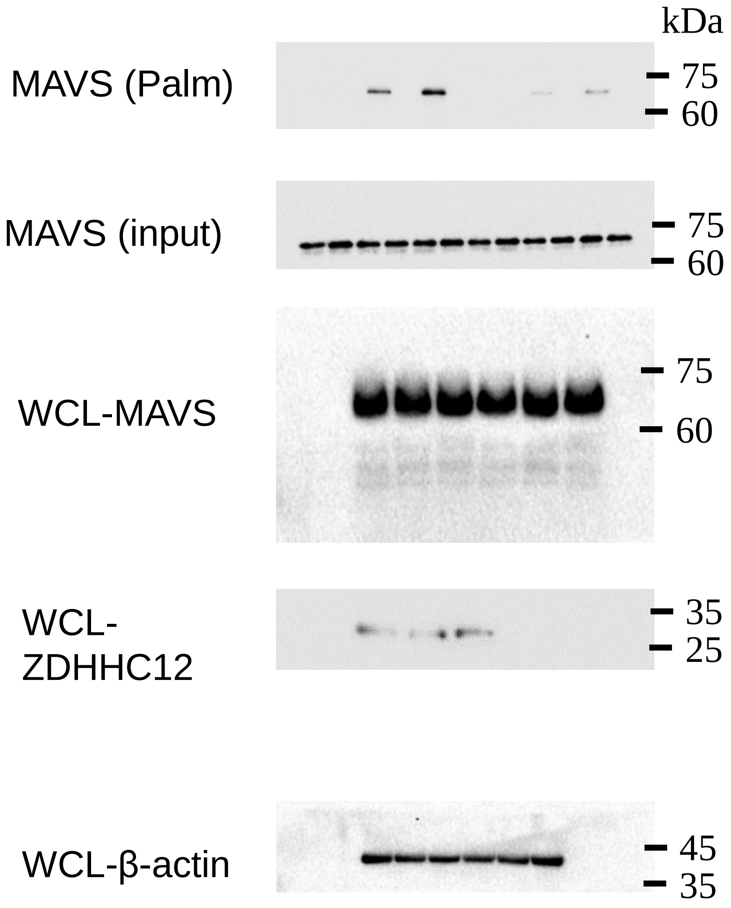

Full unedited blot for Figure 2J

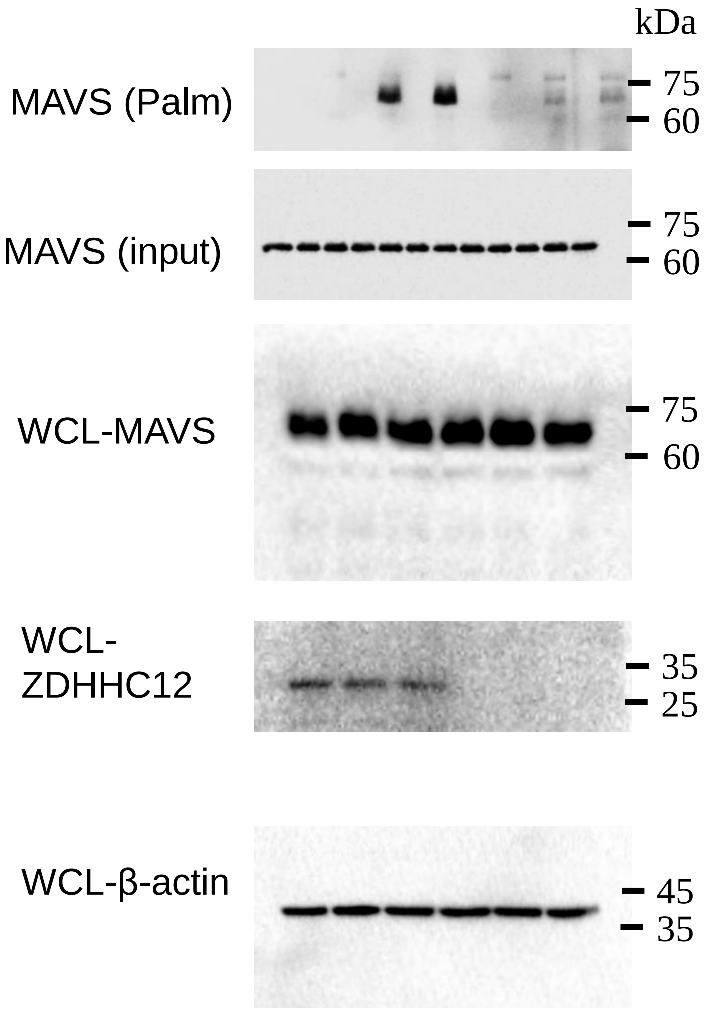

Full unedited blot for Figure 3A

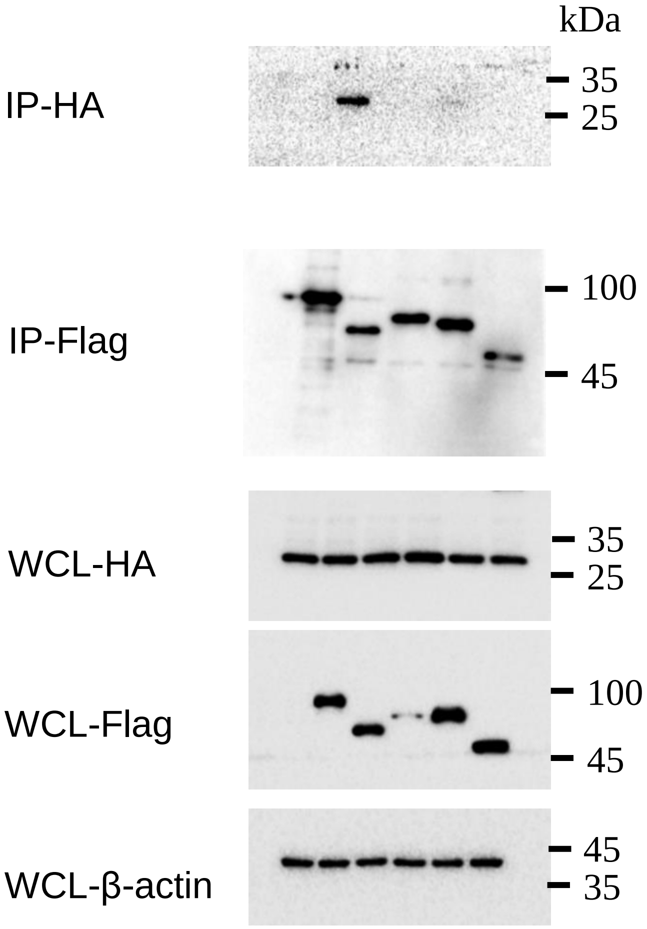

Full unedited blot for Figure 3B

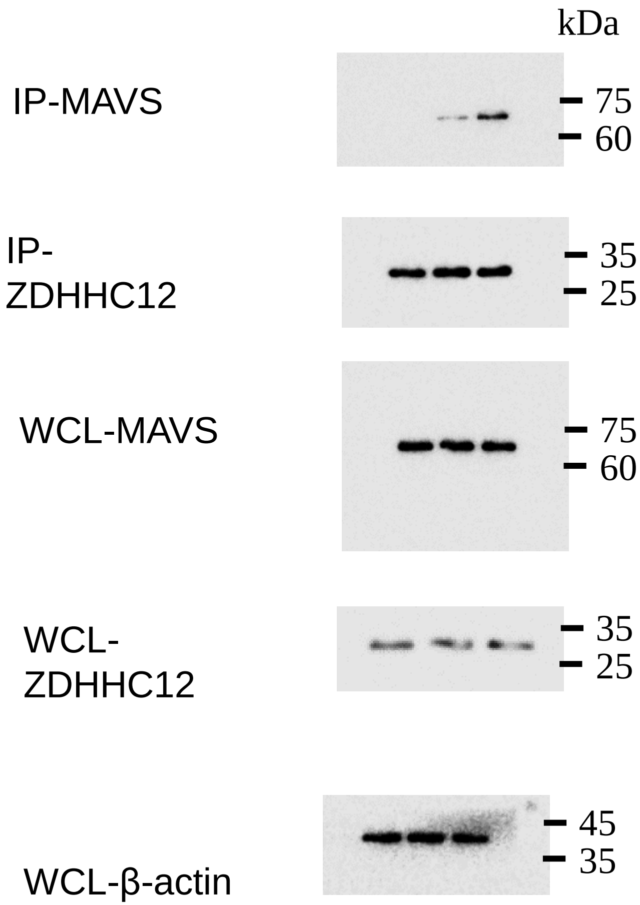

Full unedited blot for Figure 3E

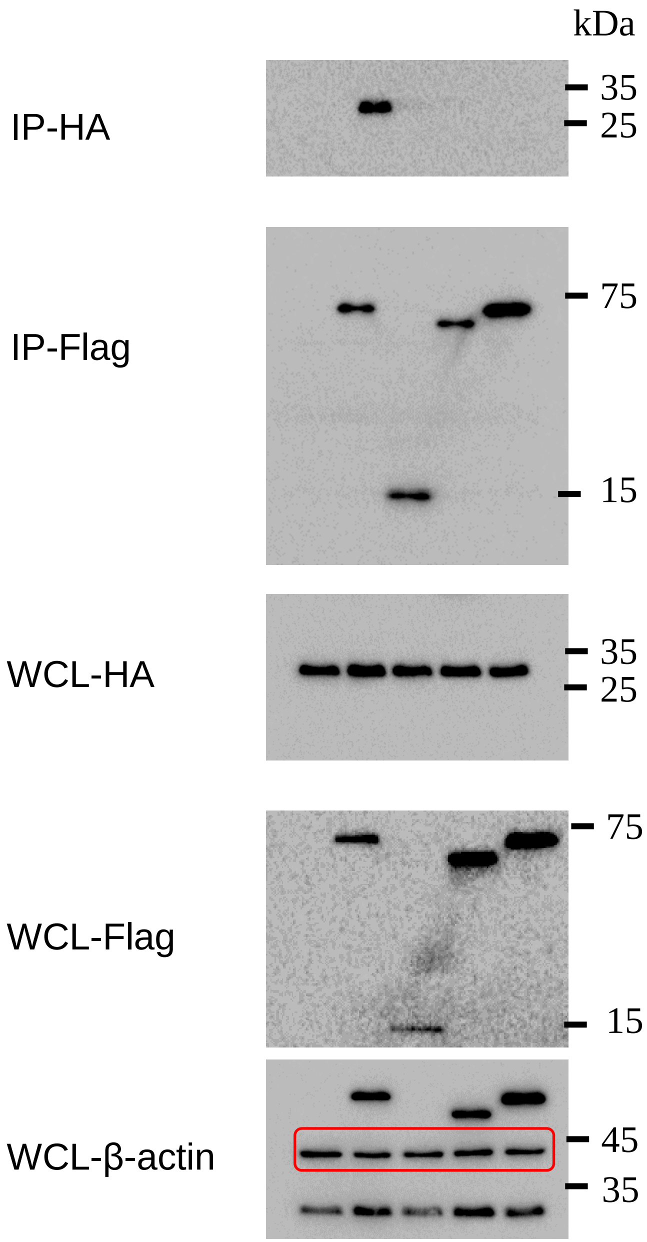

Full unedited blot for Figure 3F

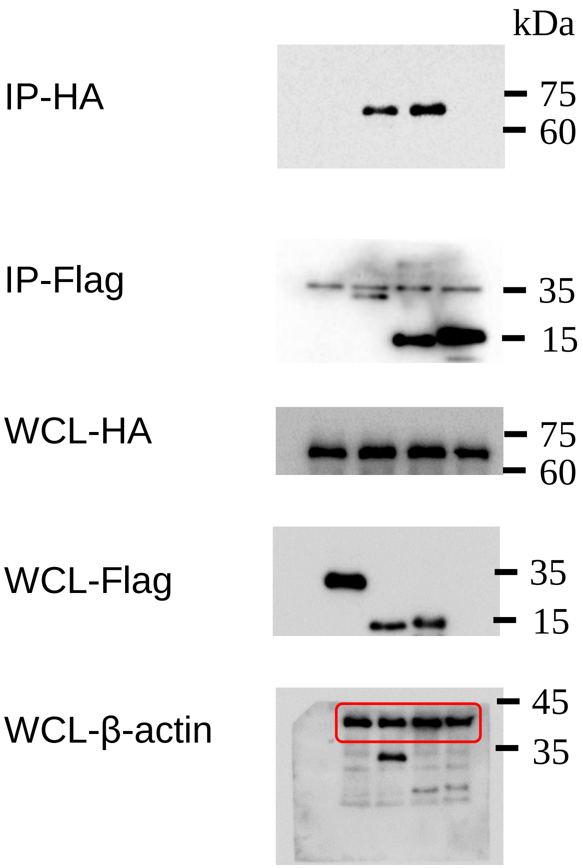

Full unedited blot for Figure 4A

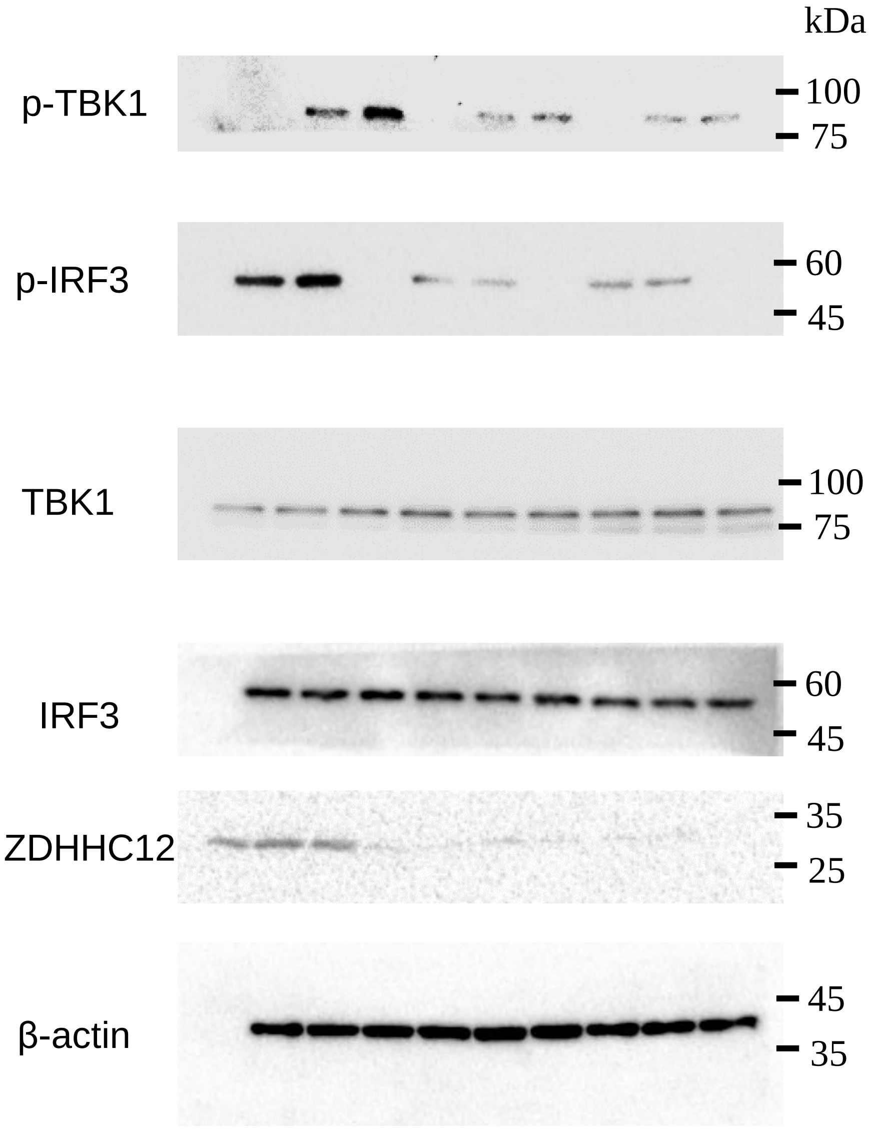

Full unedited blot for Figure 4D

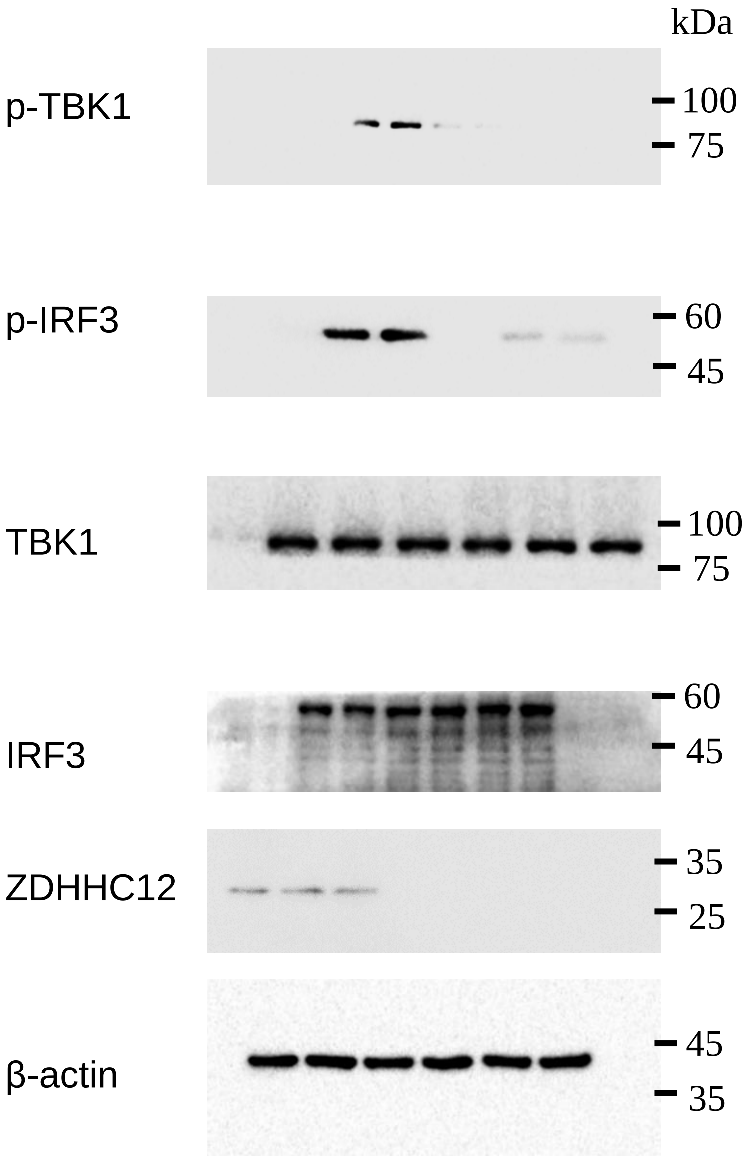

Full unedited blot for Figure 4J

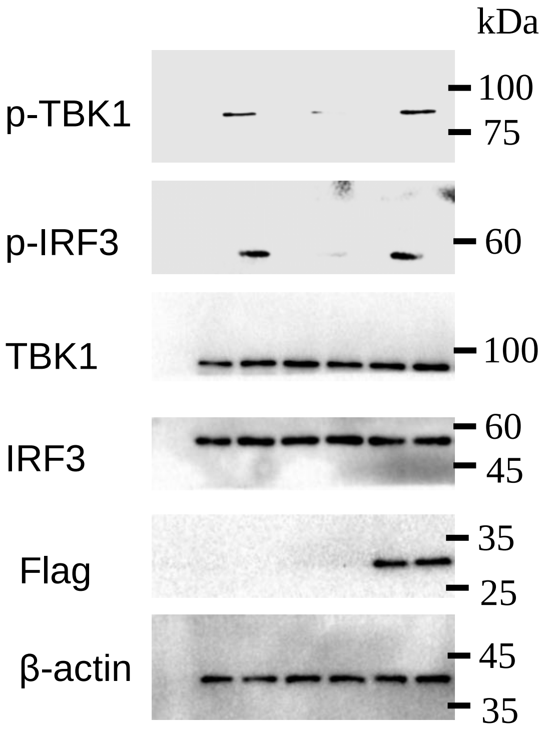

Full unedited blot for Figure 4L

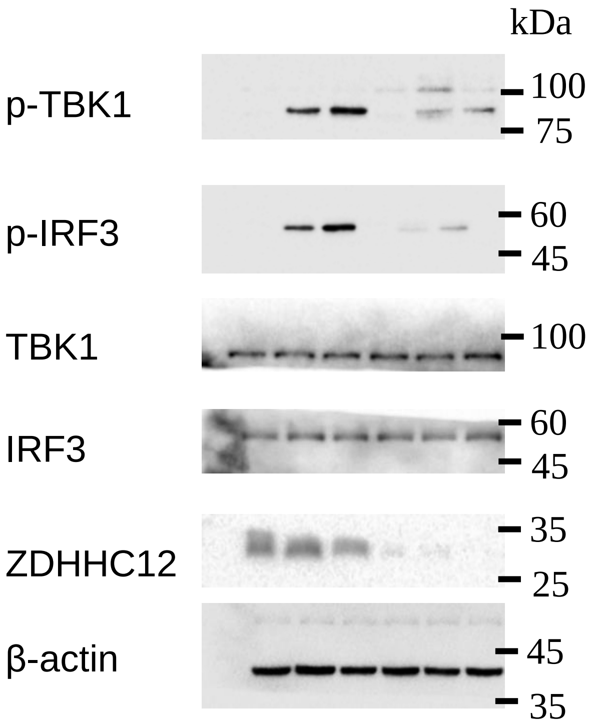

Full unedited blot for Figure 4M

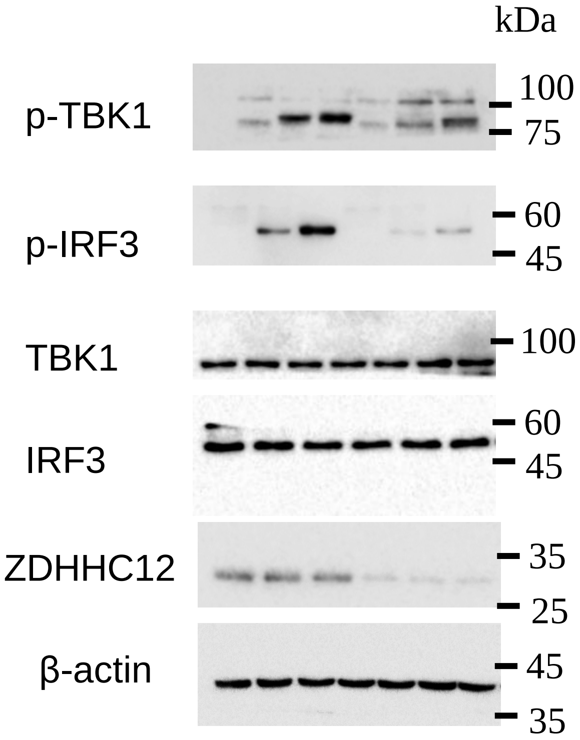

Full unedited blot for Figure 5A

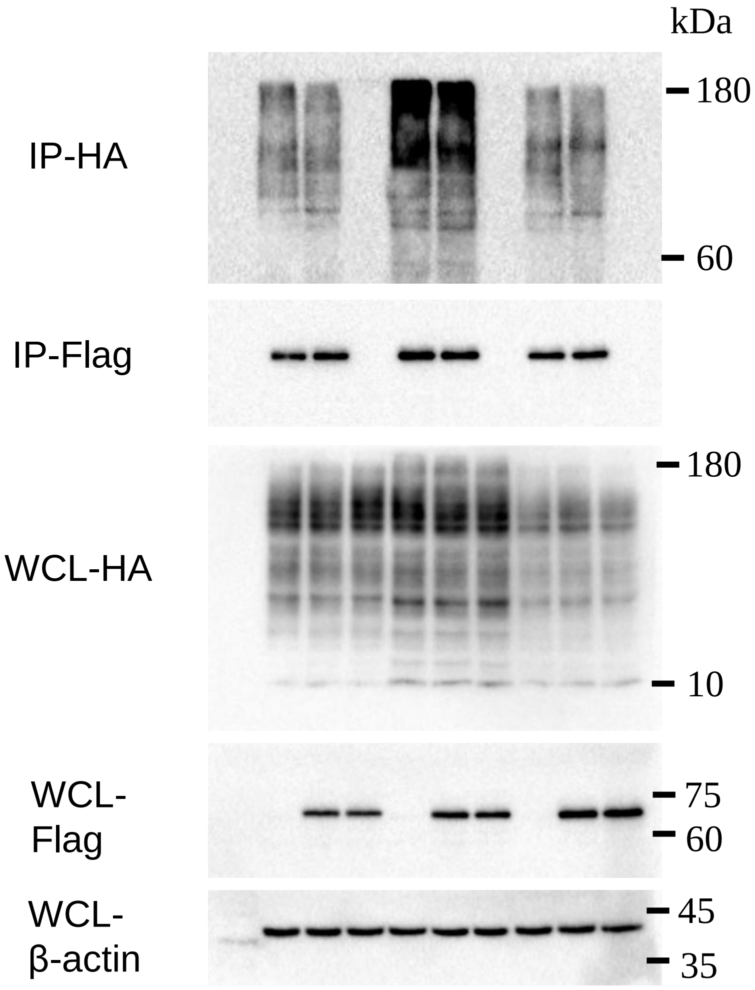

Full unedited blot for Figure 5C

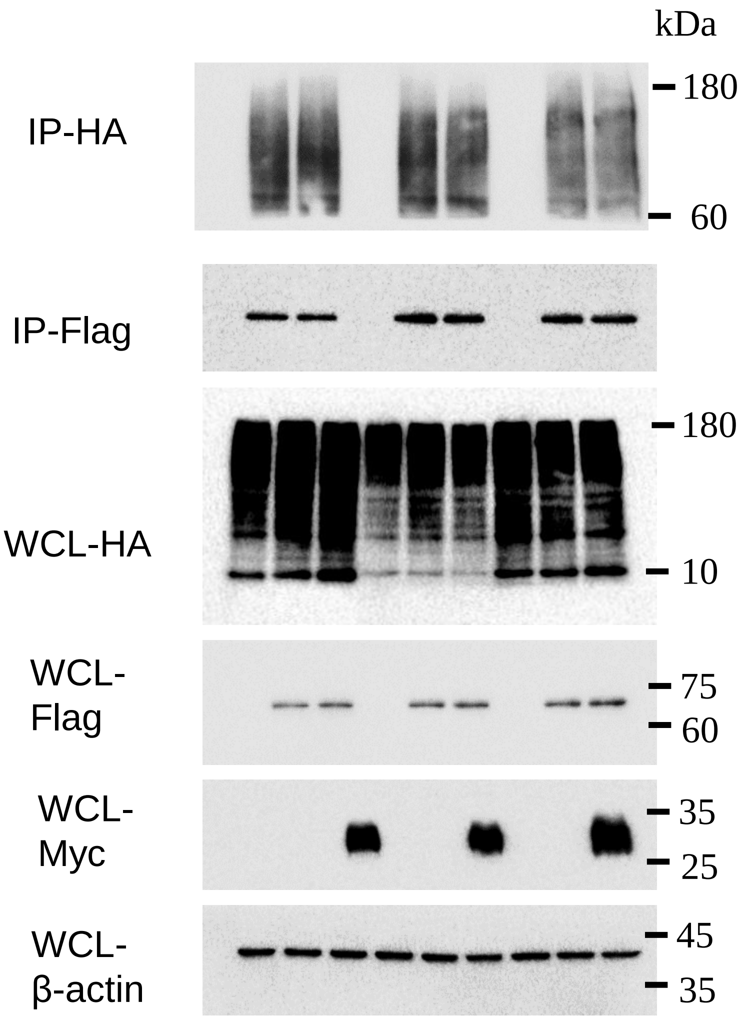

Full unedited blot for Figure 5E

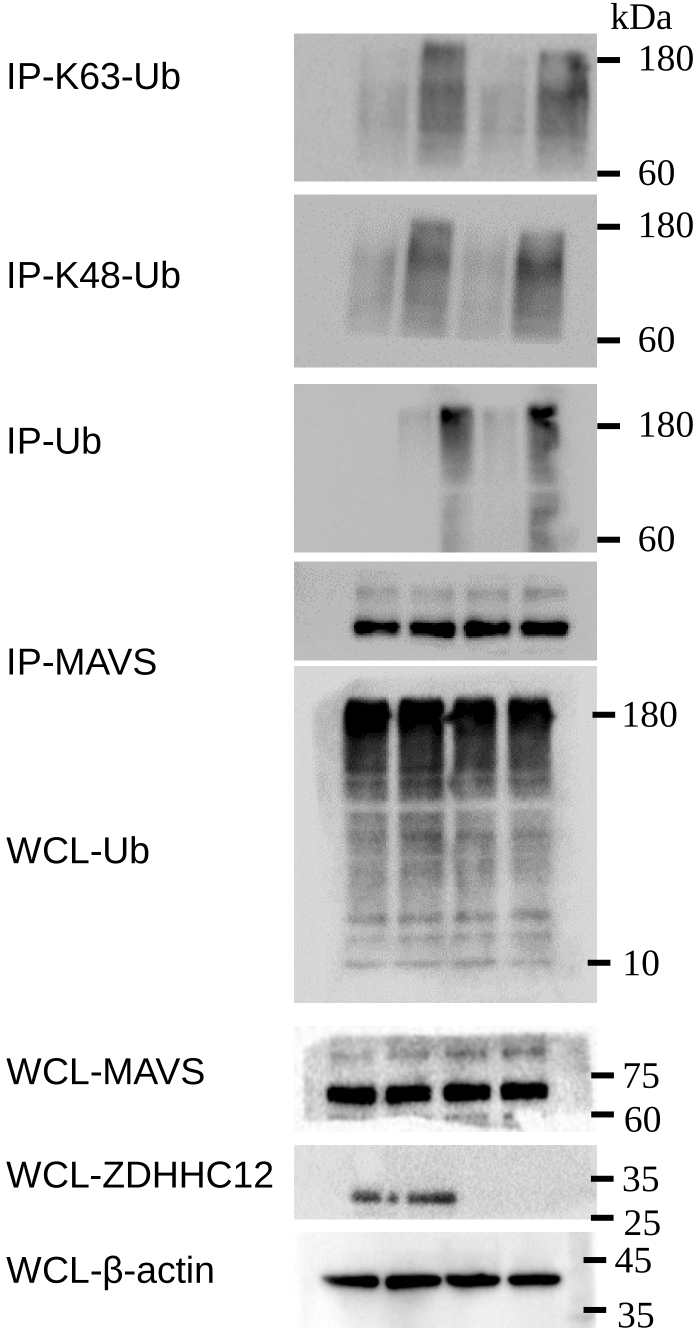

Full unedited blot for Figure 5G

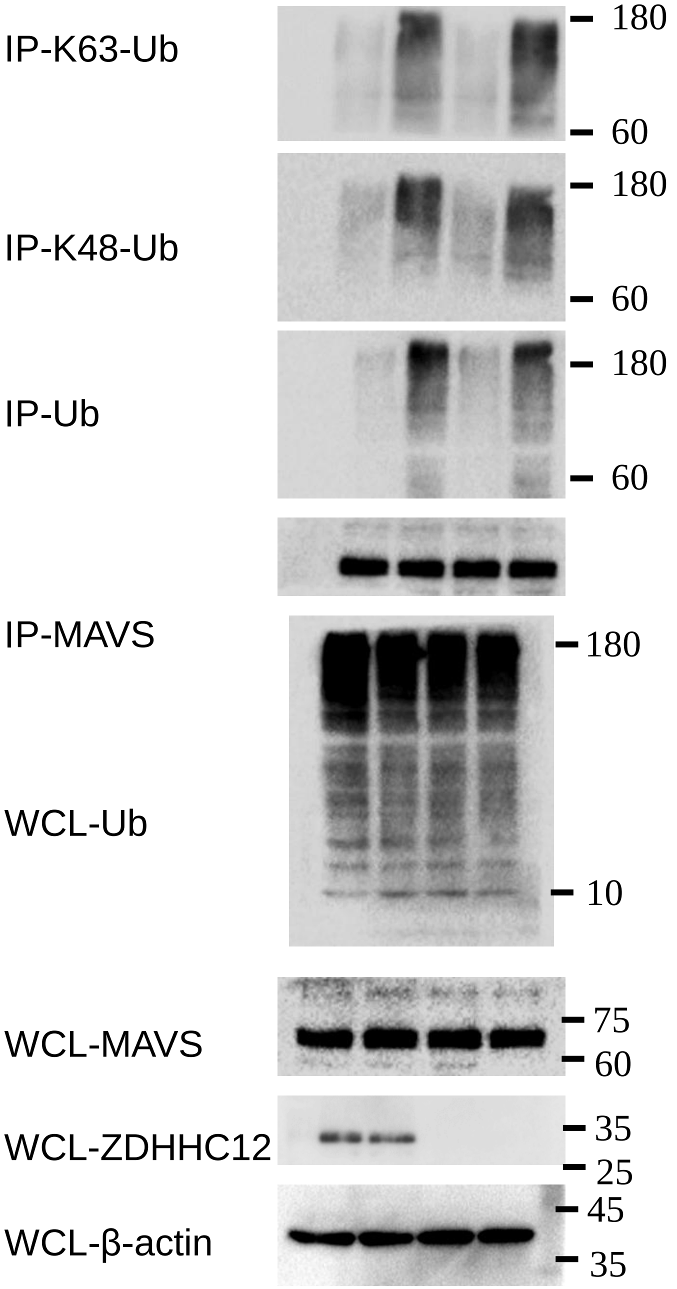

Full unedited blot for Figure 5I

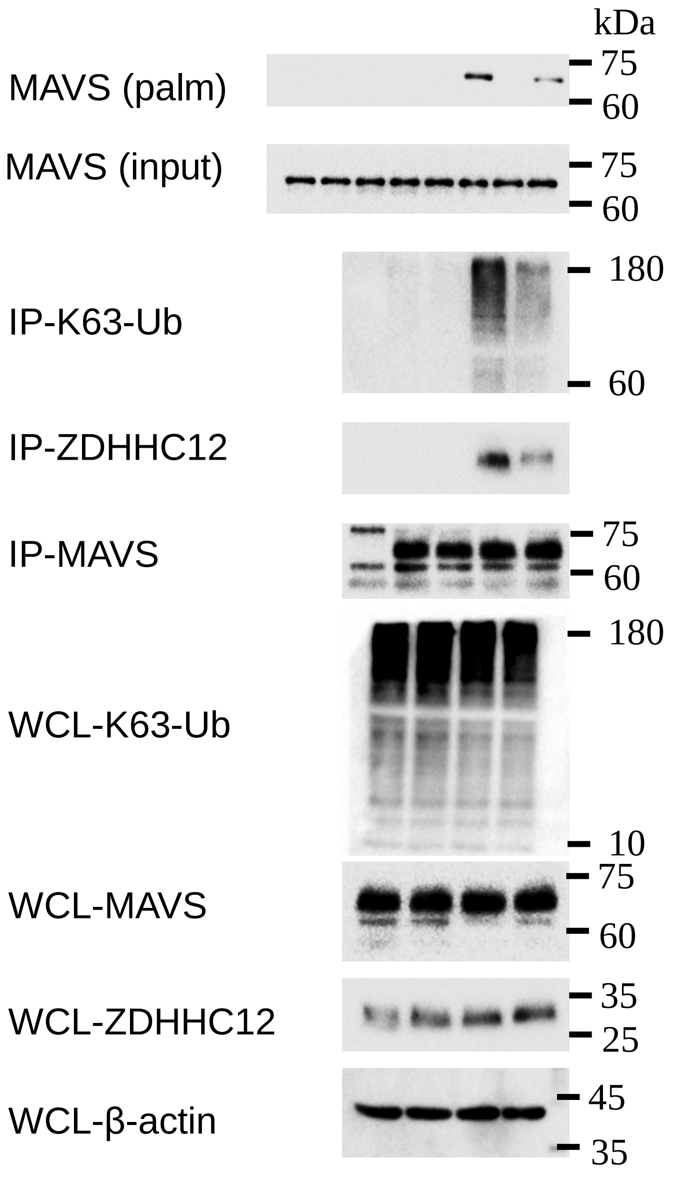

Full unedited blot for Figure 5J

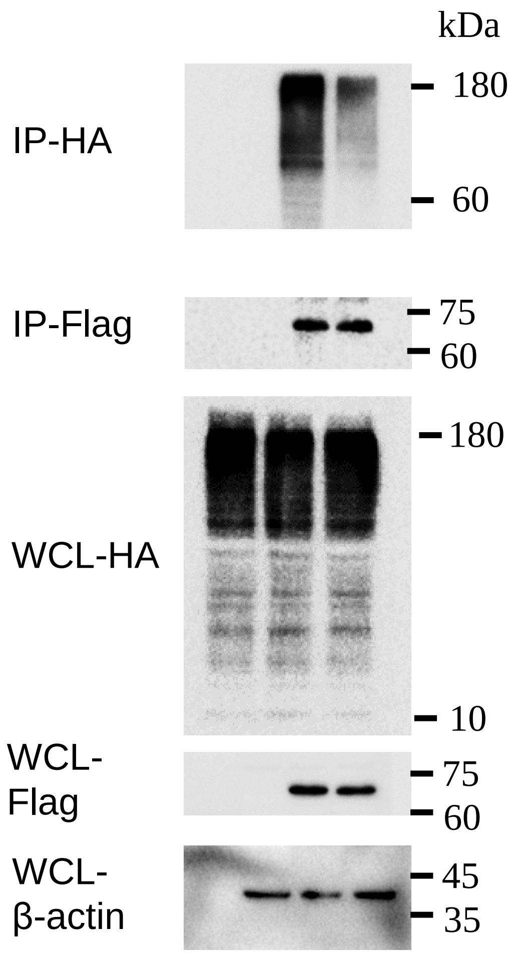

Full unedited blot for Figure 5K

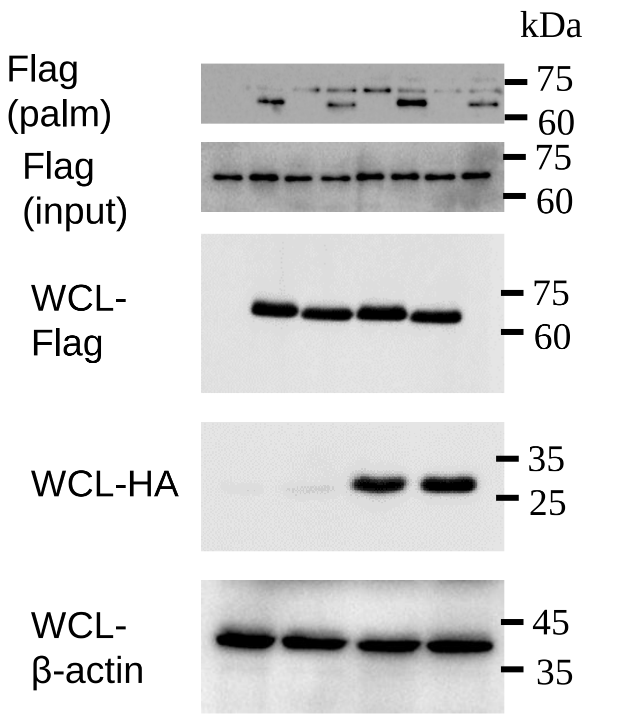

Full unedited blot for Figure 6A

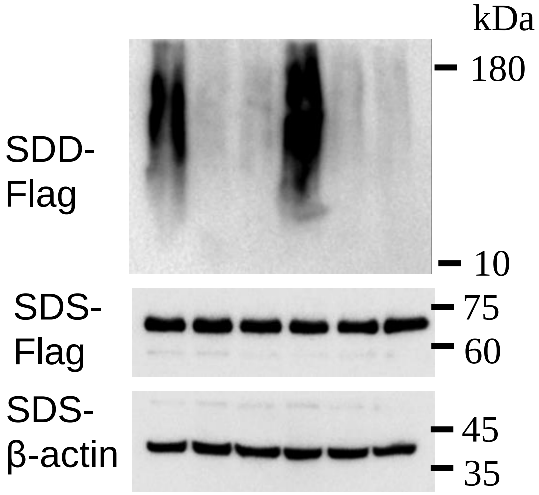

Full unedited blot for Figure 6F

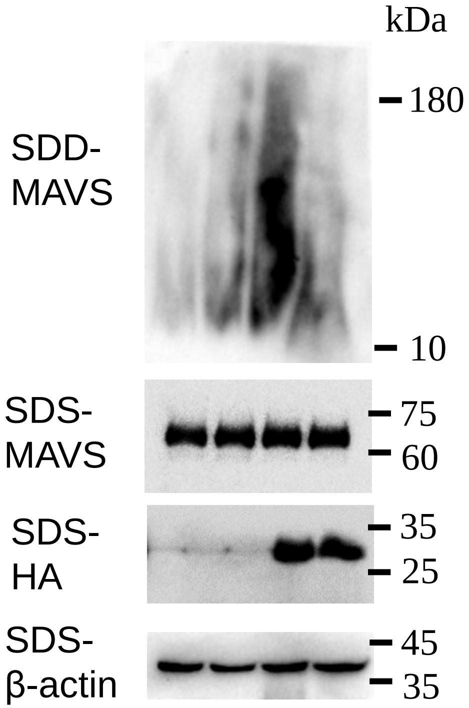

Full unedited blot for Figure 6G

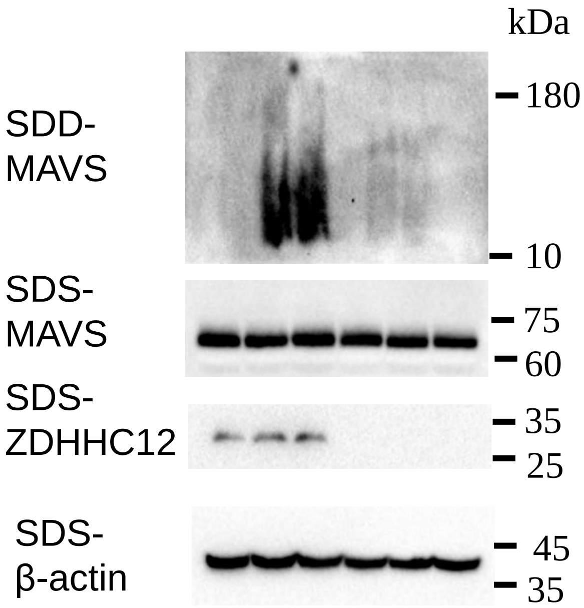

Full unedited blot for Figure 6H

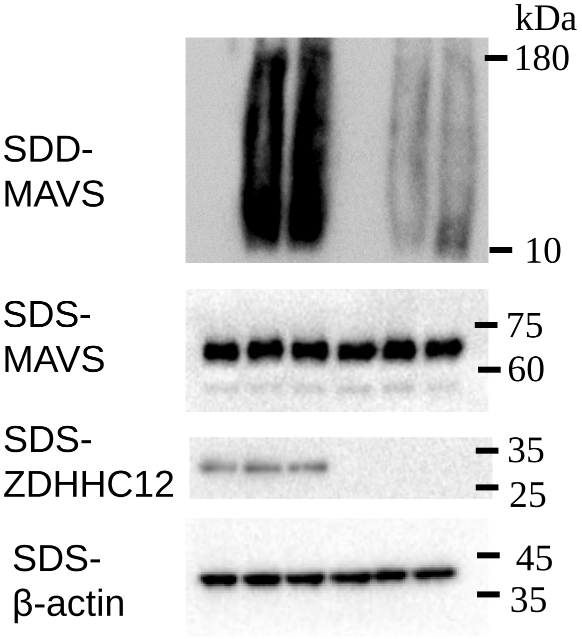

Full unedited blot for Figure 6I

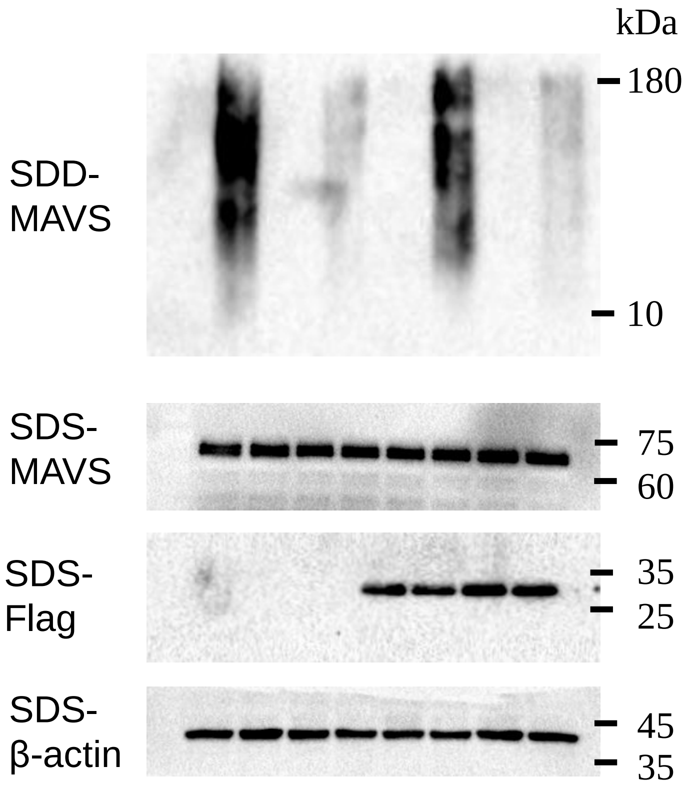

Full unedited blot for Figure 6J

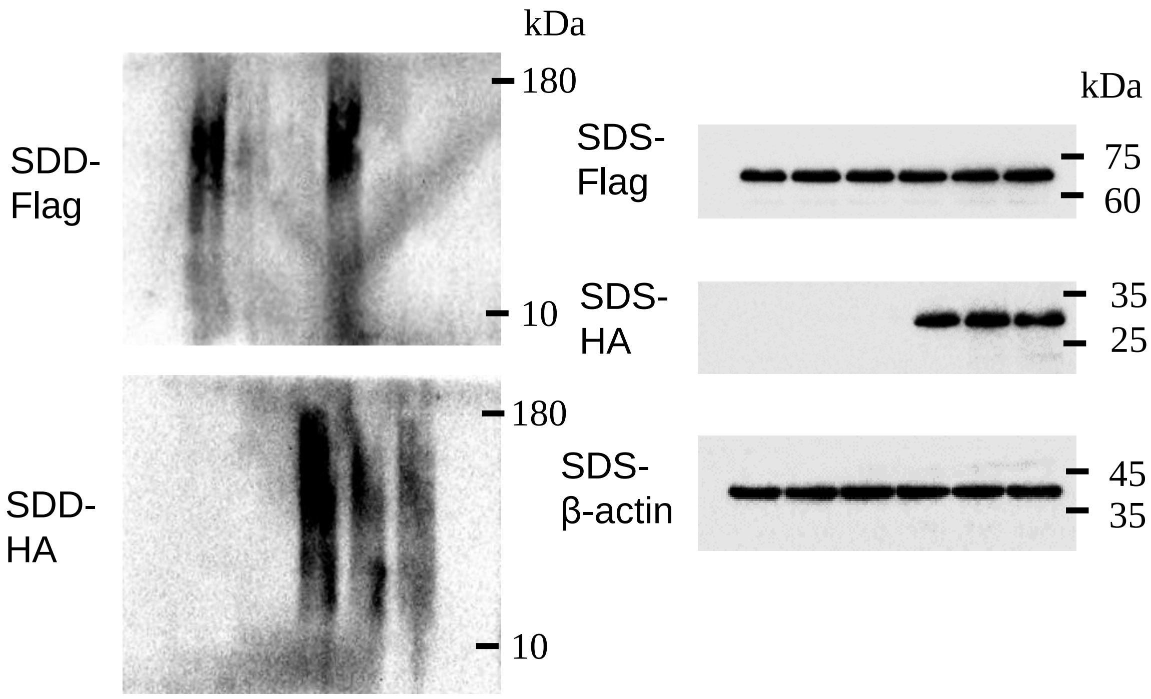

Full unedited blot for Figure 6K

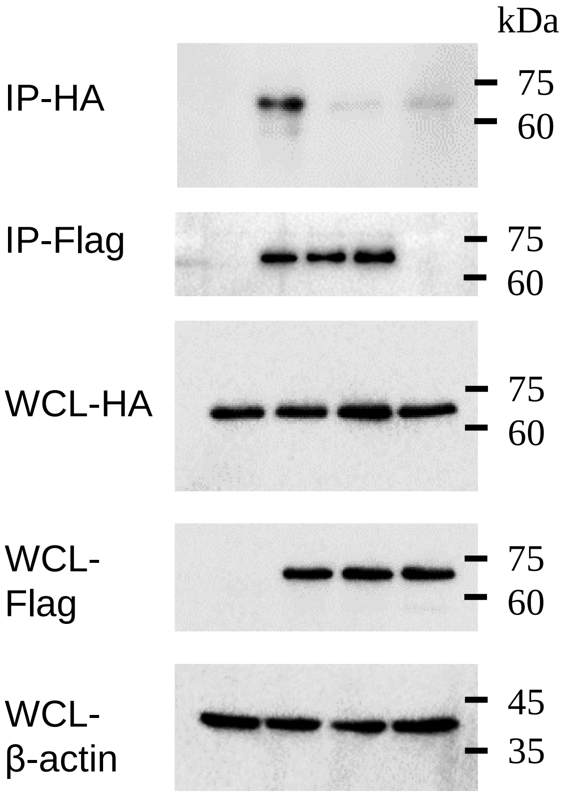

Full unedited blot for Figure 6L

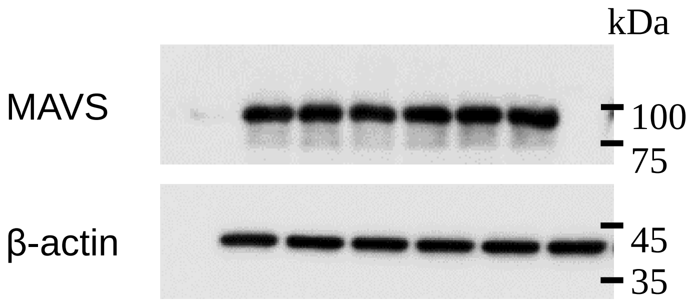

Full unedited blot for Figure 6M

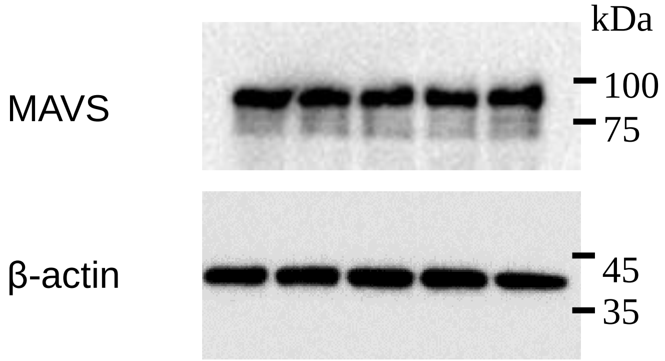

Full unedited blot for Figure 7C

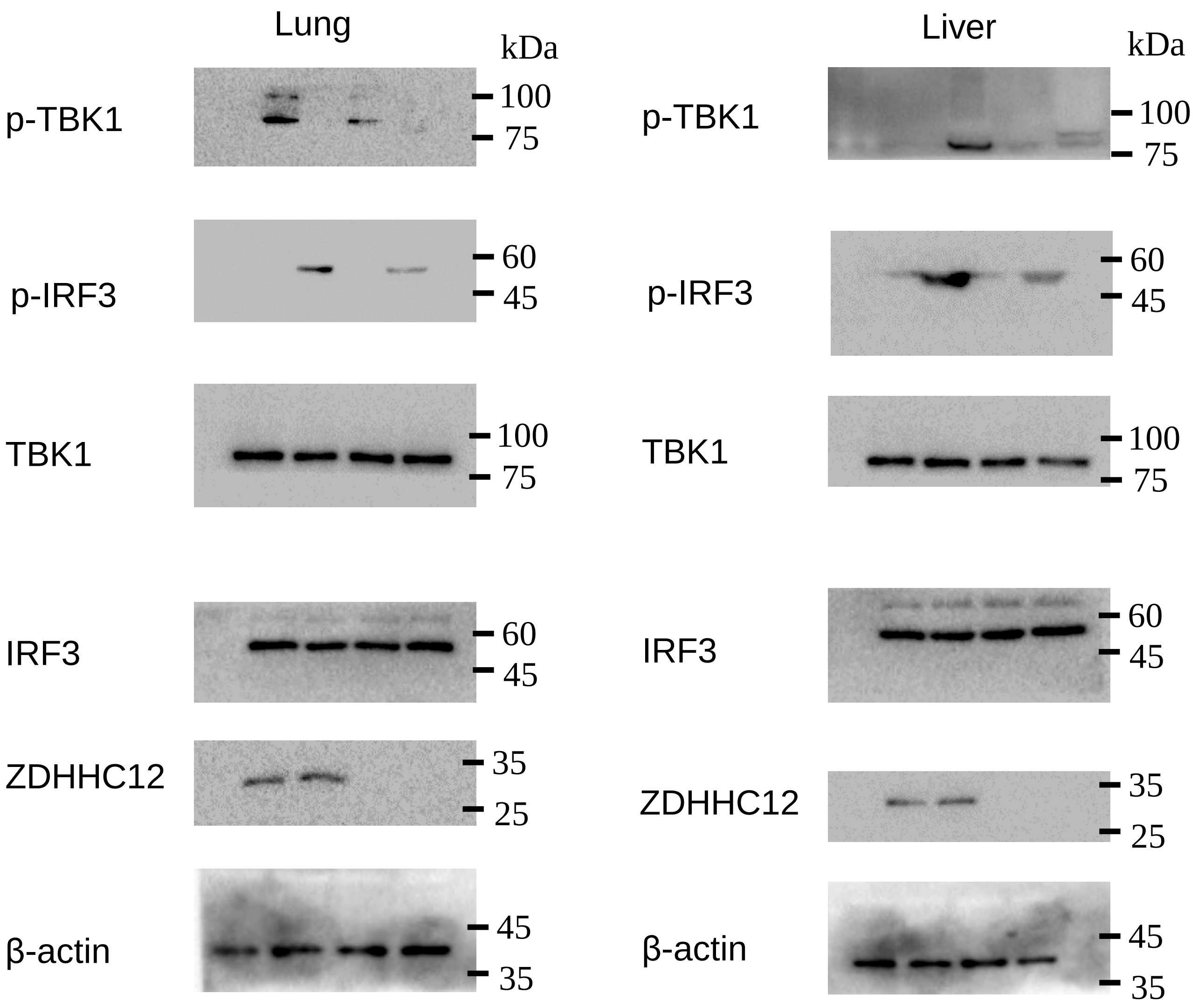

Full unedited blot for S2B

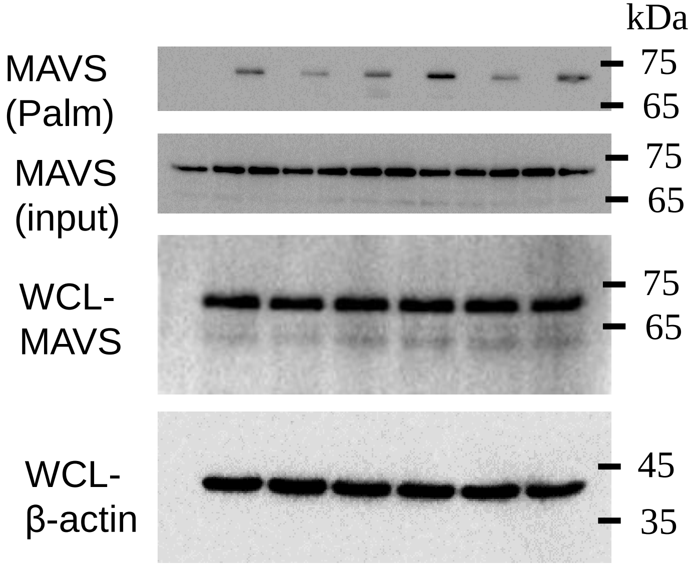

Full unedited blot for S2E

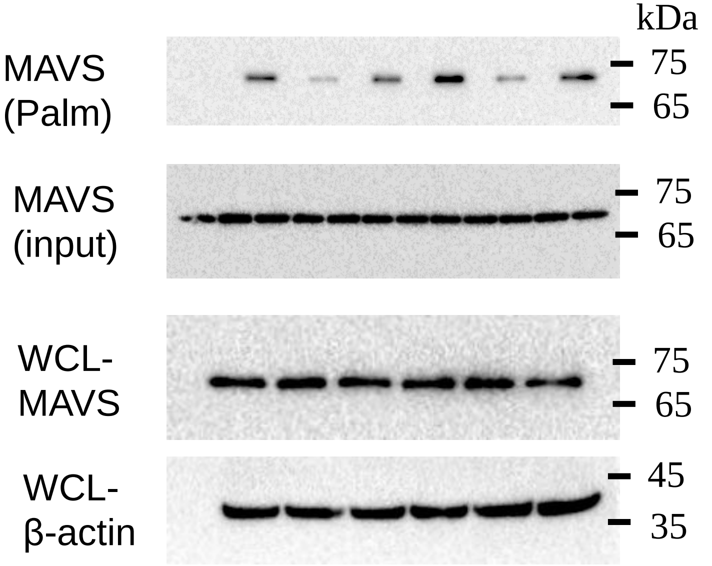

Full unedited blot for S2C

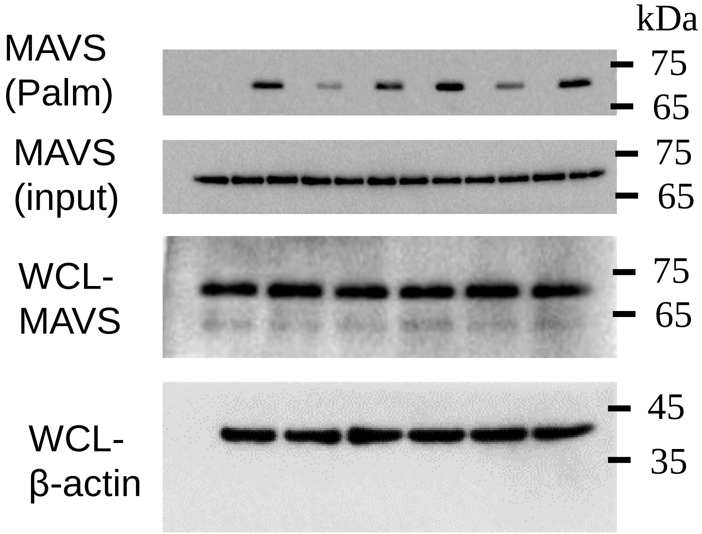

Full unedited blot for S2F

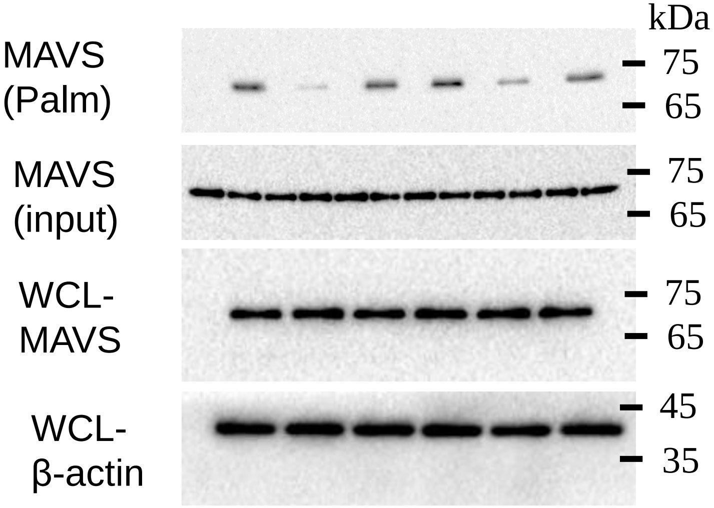

Full unedited blot for S3B

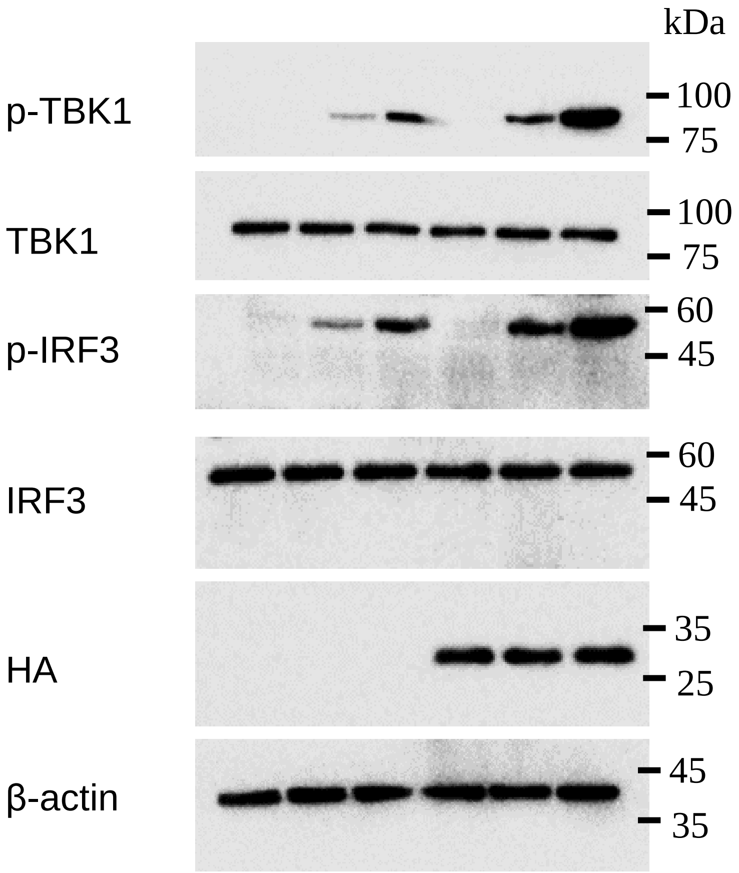

Full unedited blot for S3E

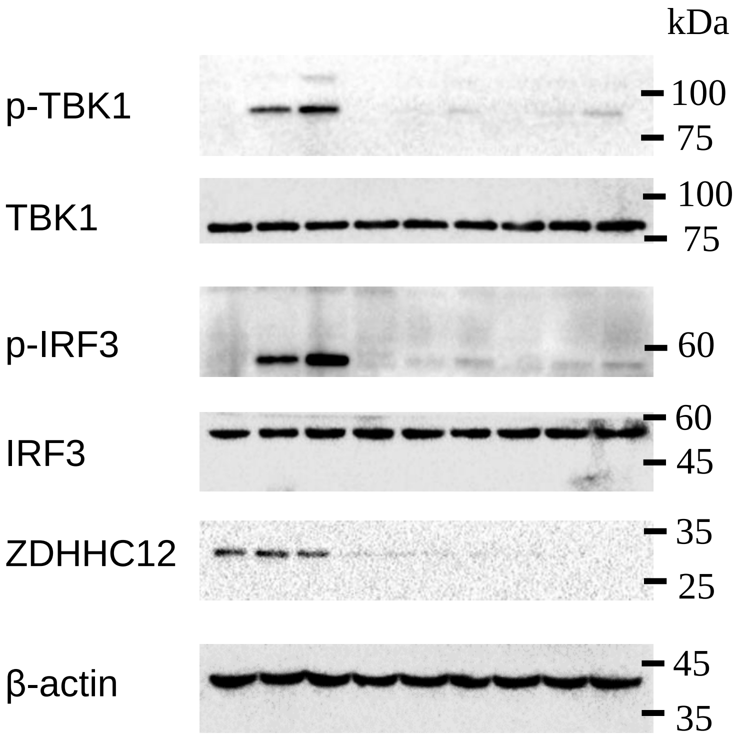

Full unedited blot for S3G

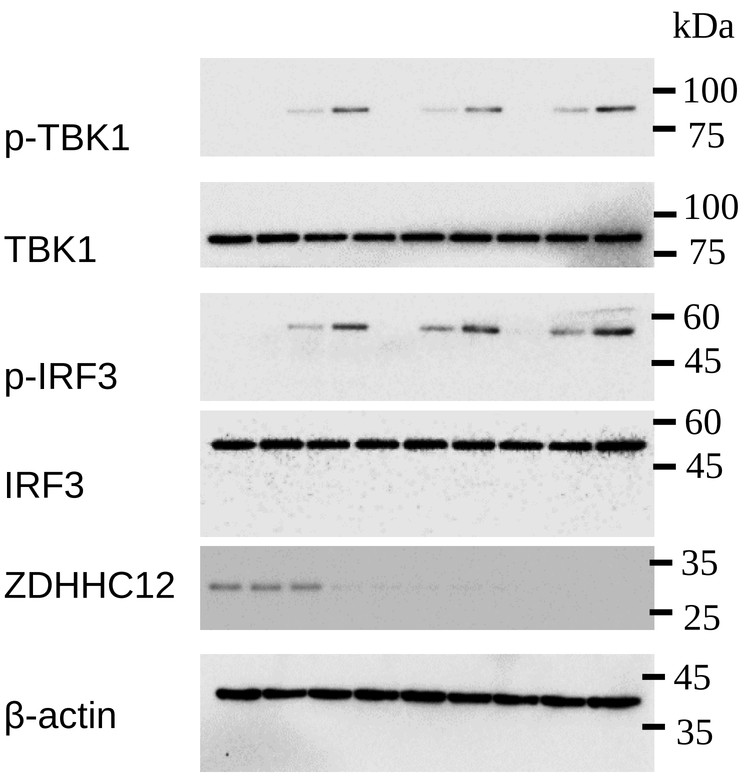

Full unedited blot for S3I

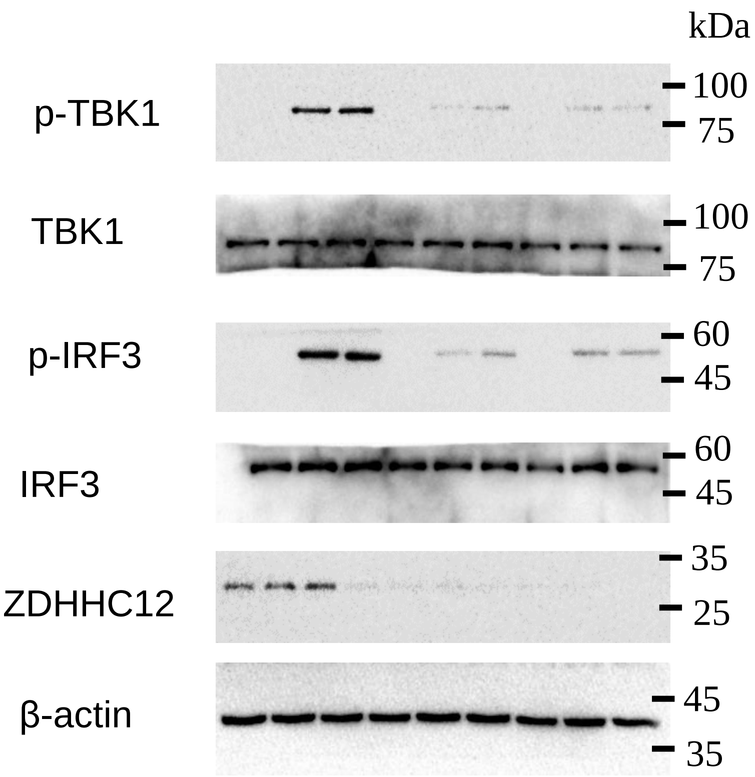

Full unedited blot for S3K

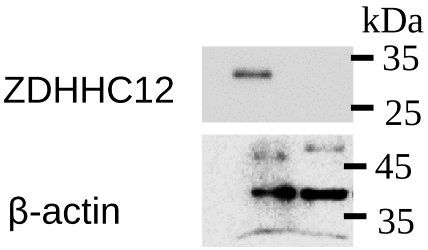

Full unedited blot for S4A

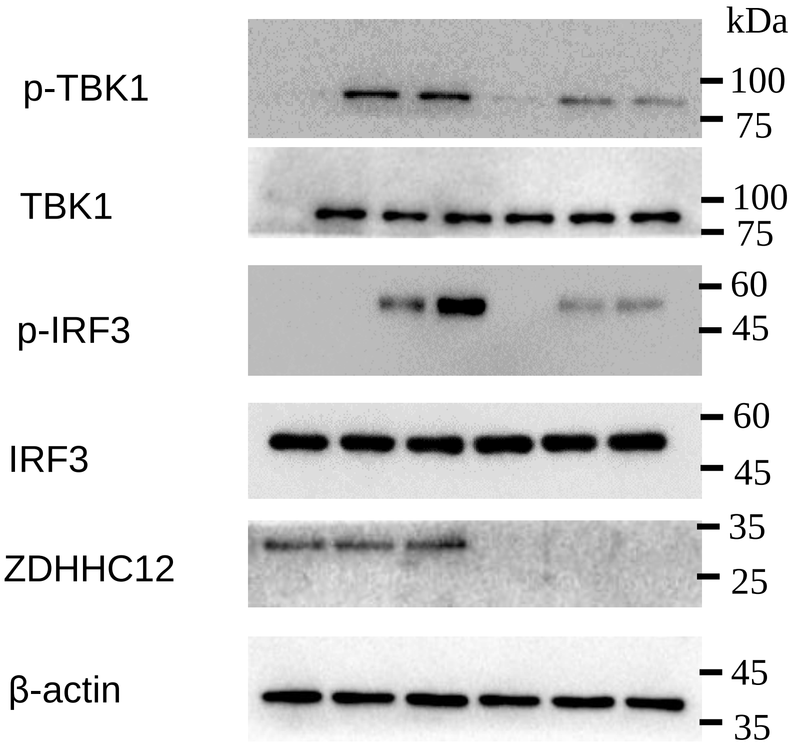

Full unedited blot for S6A

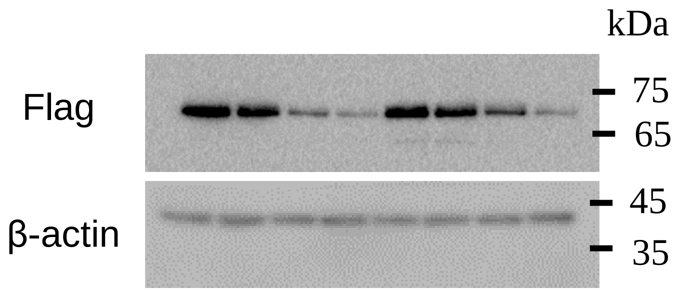

Full unedited blot for S6B

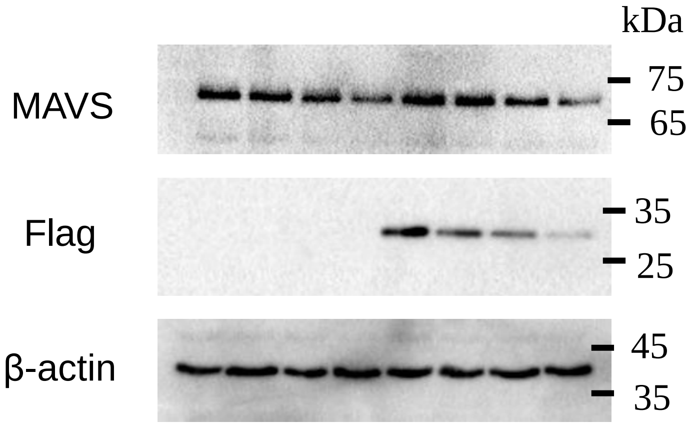

Full unedited blot for S6C

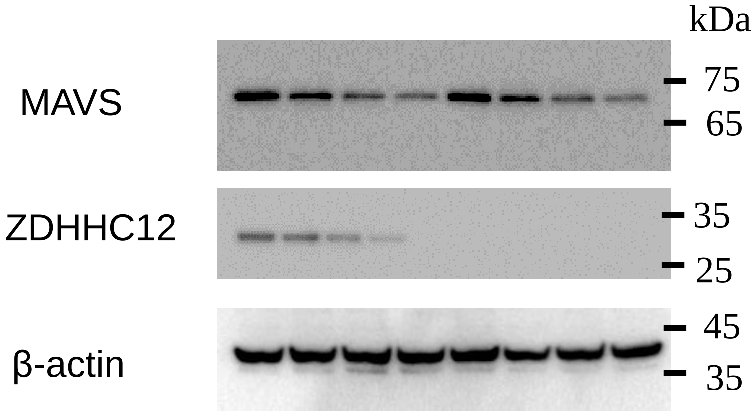

Full unedited blot for S6D

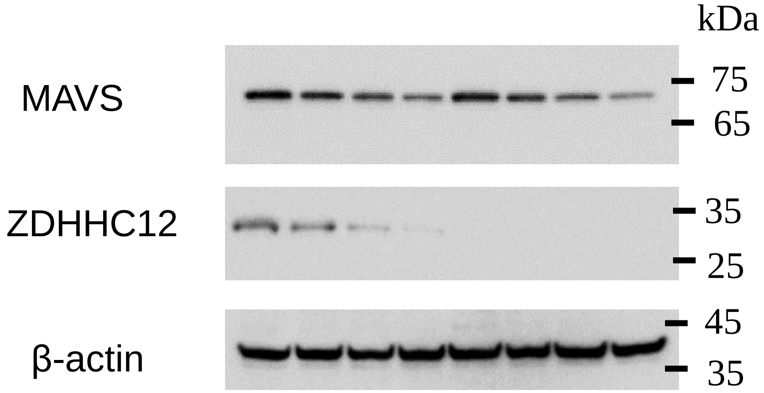

Full unedited blot for S6E

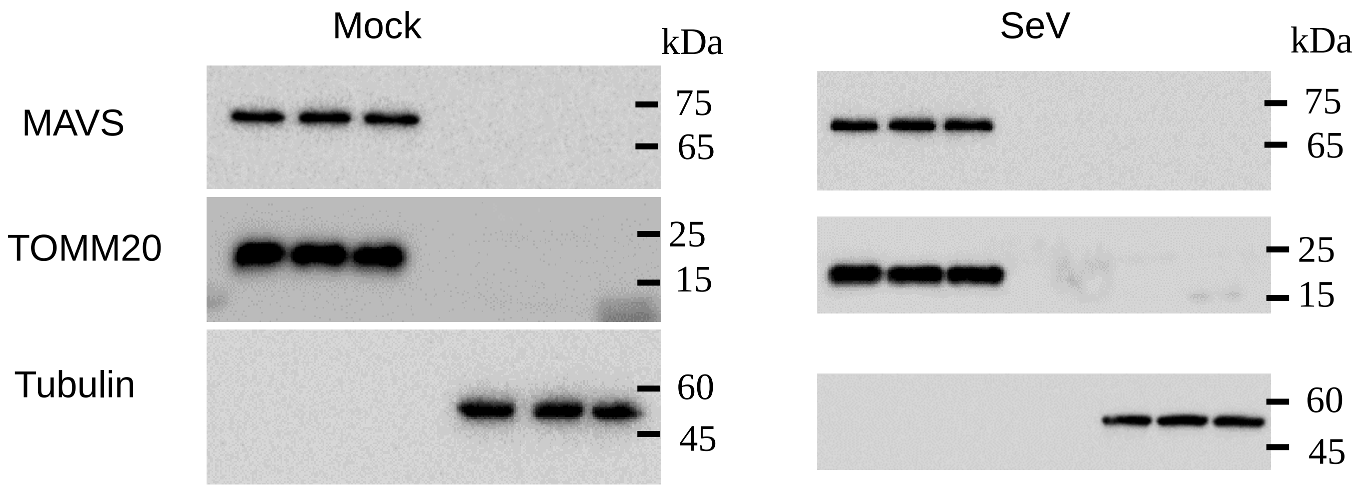

Full unedited blot for S6F

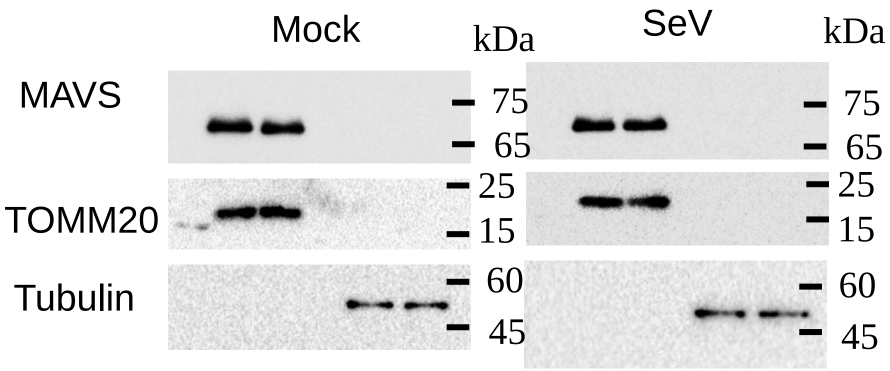

Full unedited blot for S6G

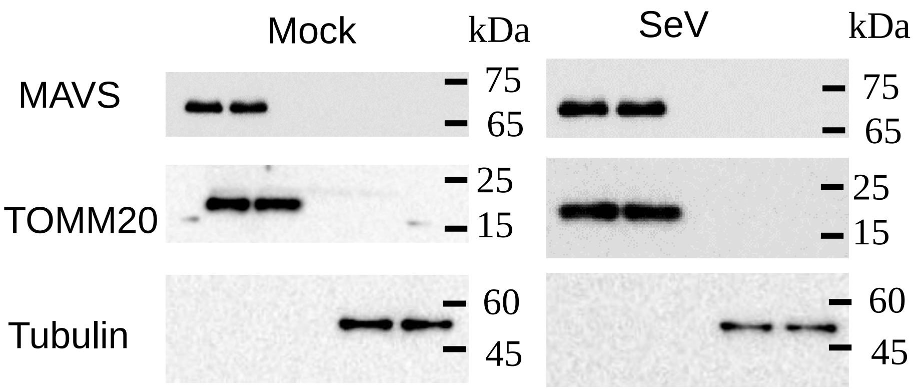

Full unedited blot for S7C

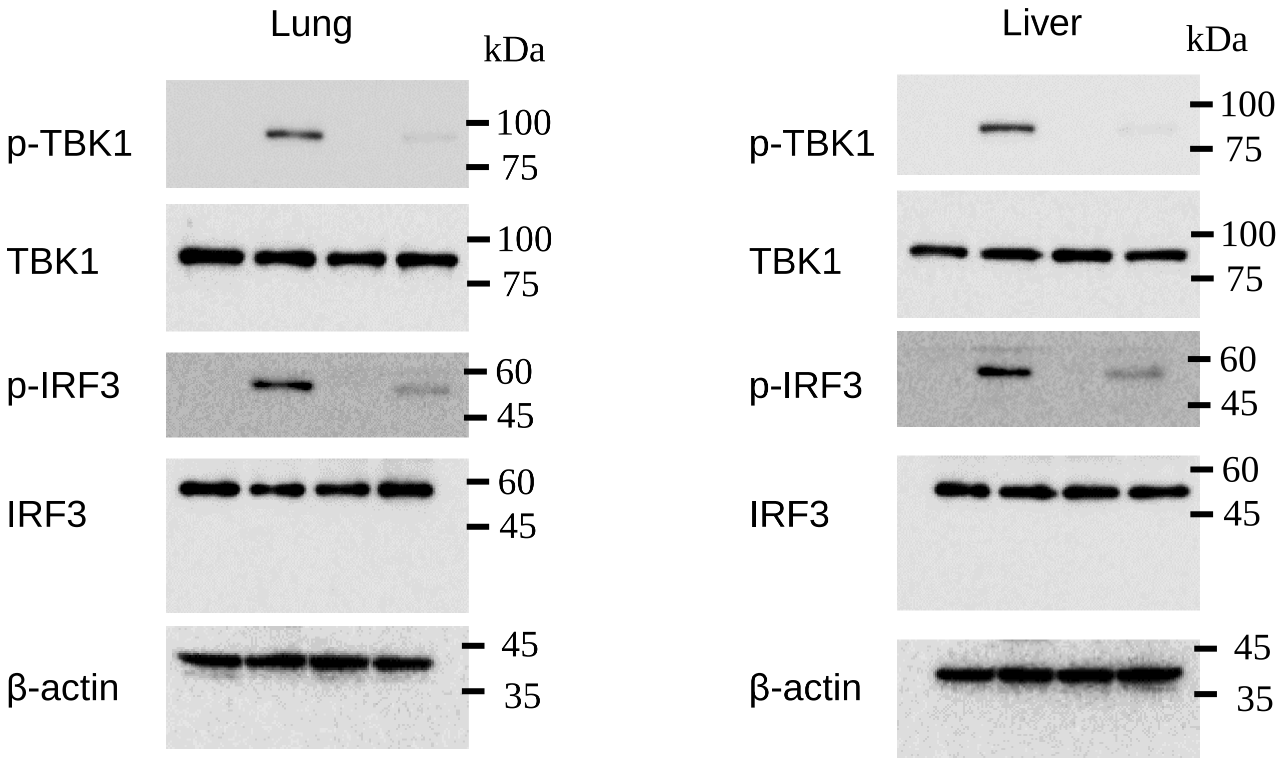

Supplement: Unedited blot and gel images [file jci-134-177924-s009.pdf]
